# Solution, Solid‐State, and Computational Analysis of Agostic Interactions in a Coherent Set of Low‐Coordinate Rhodium(III) and Iridium(III) Complexes

**DOI:** 10.1002/chem.201705990

**Published:** 2018-02-28

**Authors:** Richard C. Knighton, Jack Emerson‐King, Jonathan P. Rourke, C. André Ohlin, Adrian B. Chaplin

**Affiliations:** ^1^ Department of Chemistry University of Warwick Gibbet Hill Road Coventry CV4 7AL UK; ^2^ Department of Chemistry Umeå University Linneausvag 6 907 34 Umeå Sweden

**Keywords:** agostic interactions, crystal engineering, ligand effects, organometallic chemistry, X-ray diffraction

## Abstract

A homologous family of low‐coordinate complexes of the formulation *trans*‐[M(2,2′‐biphenyl)(PR_3_)_2_][BAr^F^_4_] (M=Rh, Ir; R=Ph, Cy, *i*Pr, *i*Bu) has been prepared and extensively structurally characterised. Enabled through a comprehensive set of solution phase (VT ^1^H and ^31^P NMR spectroscopy) and solid‐state (single crystal X‐ray diffraction) data, and analysis in silico (DFT‐based NBO and QTAIM analysis), the structural features of the constituent agostic interactions have been systematically interrogated. The combined data substantiates the adoption of stronger agostic interactions for the Ir^III^ compared to Rh^III^ complexes and, with respect to the phosphine ligands, in the order P*i*Bu_3_>PCy_3_>P*i*Pr_3_>PPh_3_. In addition to these structure–property relationships, the effect of crystal packing on the agostic interactions was investigated in the tricyclohexylphosphine complexes. Compression of the associated cations, through inclusion of a more bulky solvent molecule (1,2‐difluorobenzene vs. CH_2_Cl_2_) in the lattice or collection of data at very low temperature (25 vs. 150 K), lead to small but statistically significant shortening of the M−H−C distances.

## Introduction

The coordination chemistry of C−H bonds is an important facet of contemporary organometallic chemistry.^1^, ^2^, ^3^ Adoption of 3‐centre‐2‐electron M−H−C bonds can help stabilise otherwise reactive low‐coordinate metal complexes that are implicated in many catalytic reactions, and from a fundamental perspective represent an opportunity to gain insight into transition‐metal‐mediated C−H bond activation reactions.^4^ As a consequence of the weakly interacting nature of C−H bonds, well‐defined examples are almost exclusively limited to intramolecular systems that are promoted through the chelate effect. As first articulated by Brookhart and Green, the consistent interactions are termed “agostic” and typified by M−H−C contacts of <3 Å.^1^, ^5^ The characterisation of alkane complexes is significantly more experimentally demanding, but has been achieved in solution using time‐resolved spectroscopic methods under low temperature regimes,^3^, ^6^ and recently in the solid‐state by X‐ray crystallography through application of single‐crystal to single‐crystal transformations.^7^

**Figure 1:**
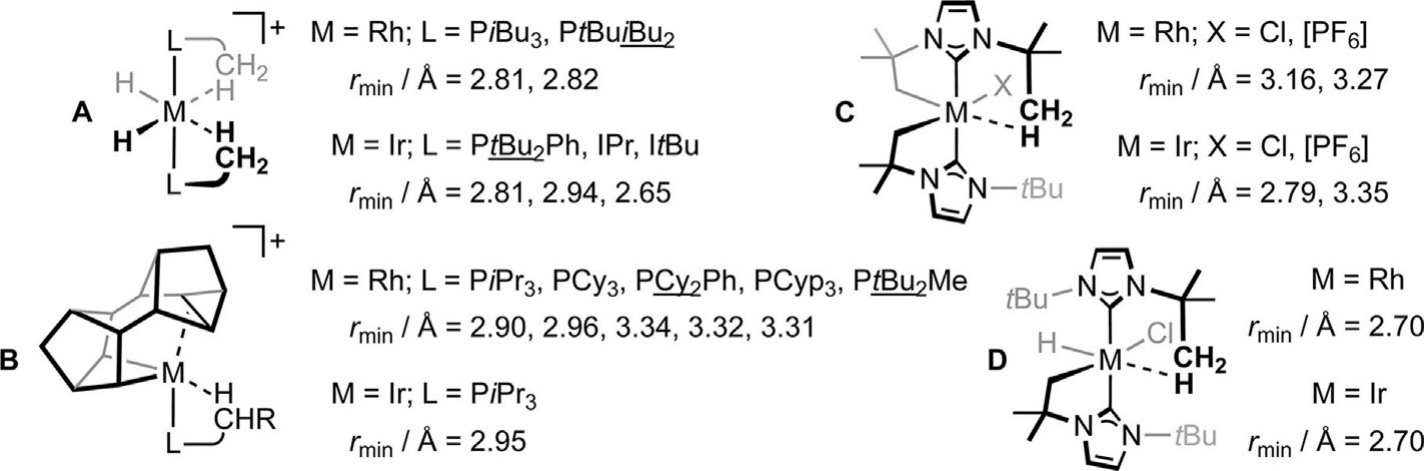
Structurally related sets of low‐coordinate Rh^III^ and Ir^III^ complexes featuring agostic interactions (*r*_min_=closest M−H−C contact) characterised in the solid‐state by X‐ray diffraction.

Given that the development of C−H bond activation chemistry has been closely connected with the organometallic chemistry of rhodium and iridium,^8^ it is perhaps unsurprising that a large number of well‐defined complexes of these group 9 metals featuring agostic interactions have been reported.^9^, ^10^, ^11^, ^12^, ^13^, ^14^, ^15^ Indeed amongst known examples a number of families can be identified, with M^III^ complexes of the formulation *trans*,*cis*‐[ML_2_H_2_]^+^ (**A**: M=Rh, Ir; L=phosphine or NHC),^9^ [M(Binor‐S)L]^+^ (**B**: Binor‐*S*=1,2,4,5,6,8‐dimetheno‐S‐indacene; L=phosphine),^10^ and bearing cyclometalated I*t*Bu (**C**, **D**) the most outstanding (Figure 1).^11^ Structurally related clusters of this nature are of interest to gauge an understanding of the effect of the metal alongside subtle variations of the ligand composition on the constituent agostic interactions. Unfortunately, as they currently stand, neither the size nor specific membership of these three families is well suited to an analysis of this nature.

**Figure 2:**
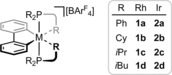
Low‐coordinate rhodium^III^ and iridium^III^ complexes studied.

Whilst it is conceivably possible to extend the membership of the aforementioned sets, the synthetic chemistry underlying the isolation of these highly reactive organometallics presents a number of practical challenges. Recognising a degree of commonality amongst **A**–**C** and others,^12^ namely sawhorse metal geometries with high *trans* influence ligands in the *cis*‐equatorial positions, and building upon the previous report of low‐coordinate Rh^III^ complex *trans*‐[Rh(2,2′‐biphenyl)(P*i*Pr_3_)_2_][BAr^F^_4_] (**1 c**; Ar^F^=3,5‐(CF_3_)_2_C_6_H_3_),^13^ we reasoned that utilising 2,2′‐biphenyl as an ancillary ligand would be a straightforward means to gather a set of solution and solid‐state data for agostic interactions between phosphine ligand substituents and Rh^III^ and Ir^III^ centres. To this end, and with a view to elucidating structure‐property relationships within such data, we report the synthesis and extensive characterisation of low‐coordinate complexes of the formulation *trans*‐[M(2,2′‐biphenyl)(PR_3_)_2_][BAr^F^_4_] (M=Rh, **1**; Ir, **2**; R=Ph, **a**; Cy, **b**; *i*Pr, **c**; *i*Bu, **d**; Figure 2). This series of complexes encompasses both aryl and alkyl phosphine ligands (i.e. Ph vs. Cy), cyclic and acyclic alkyl phosphine substituents (i.e. Cy vs. *i*Pr) and the possibility to adopt both γ‐ and δ‐agostic interactions (i.e. *i*Pr vs. *i*Bu). DFT calculations have also been carried out to aid structural interrogation.

## Results and Discussion

### Synthesis

**Scheme 1:**
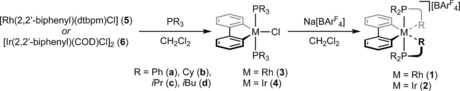
Preparation of **1** and **2**.

The preparation of **1 c** has previously been achieved via oxidative addition of biphenylene to the latent low coordinate complex [Rh(C_6_H_5_F)(P*i*Pr_3_)_2_][BAr^F^_4_].^13^ Guided by methodology developed by Jones and Crabtree for the preparation of *trans*‐[M(2,2′‐biphenyl)(PPh_3_)_2_Cl] (M=Rh, **3 a**; Ir, **4 a**),^16^, ^17^ we instead chose to employ more systematic and synthetically robust protocols that proceed via facile substitution reactions of M^III^ precursors [Rh(2,2′‐biphenyl)(dtbpm)Cl] (**5**; dtbpm=bis(di‐*tert*‐butylphosphino)methane) and [Ir(2,2′‐biphenyl)(COD)Cl]_2_ (**6**; COD=1,5‐cyclooctadiene) with the desired phosphine, followed by chloride abstraction to afford low coordinate derivatives **1** and **2**, respectively (Scheme 1). In this way, five‐coordinate intermediates **3** and **4** were readily obtained (37–83 % isolated yield) and subsequently treated with Na[BAr^F^_4_] in CH_2_Cl_2_ at RT (**3**, **4 b**–**d**) or 50 °C (**4 a**) to afford target complexes **1** and **2** that, following filtration to remove insoluble sodium salts, were isolated by slow crystallisation from CH_2_Cl_2_/pentane (liquid‐liquid diffusion at RT; 32–80 % isolated yields) and extensively characterised (vide infra). Notably, samples of **1** and **2** obtained in this way were all suitable for interrogation in the solid‐state using X‐ray diffraction.

### Solid‐state structures of 1 and 2

**Figure 3:**
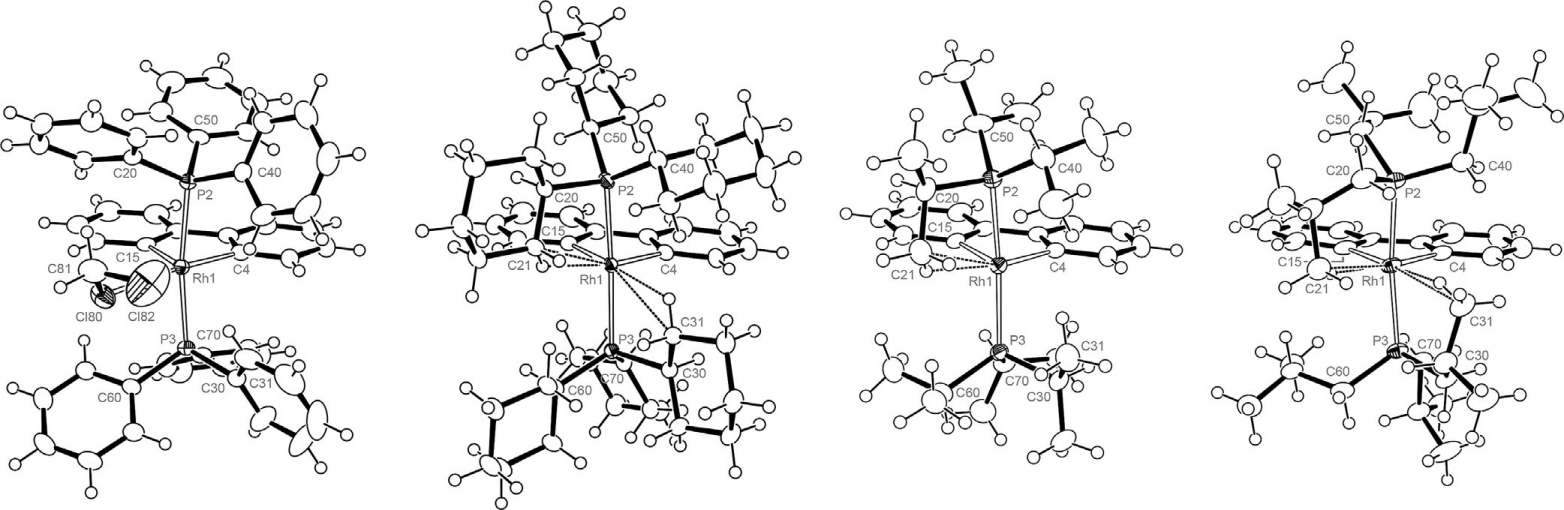
Solid‐state structures of **1 a⋅CH_2_Cl_2_**, **1 b**.CH_2_Cl_2_, **1 c**, and 1 d* (left to right). All data collected at 150 K, thermal ellipsoids at the 50 % probability level. Only one of the two unique cations shown for **1 c** (non‐disordered); anions, CH_2_Cl_2_ solvent molecules (**1 a**, **1 b**), and minor disordered components (C70/C70A Cy group in **1 b**) omitted for clarity. Selected bond lengths (Å) and angles (°): **1 a⋅CH_2_Cl_2_**, Rh1−Cl80, 2.6067(8); Cl82−H31, 3.375(2); Cl82−H41, 3.203(2); C4‐Rh1‐Cl80, 174.33(8); Equivalent metrics for **2 a⋅CH_2_Cl_2_**, Ir1−Cl80, 2.5567(12); Cl82−H31, 3.325(2); Cl82−H41, 3.134(3); C4‐Ir1‐Cl80, 175.25(11).

Single crystalline samples of **1** and **2**, grown as described above, were analysed in the solid‐state using X‐ray diffraction under typical experimental conditions (i.e. MoKα radiation, *T=*150 K).^18^ Agostic interactions are evident in the complexes bearing trialkylphosphines, while the triphenylphosphine derivatives are conspicuously obtained as adducts of solvent in the solid‐state, namely **1 a⋅CH_2_Cl_2_** and **2 a⋅CH_2_Cl_2_**.^19^ For a given phosphine, **1** and **2** are in general isomorphous; ^[20]^ those of **1** are depicted in Figure 3, with selected metrics for **1** and **2** compiled in Table 1. The salient features and experimental attempts to perturb crystal packing in **1 b** and **2 b**, are discussed below in turn.

**Table 1:** Solid‐state metrics for **1** and **2**.^[a]^

		**Selected distances [Å]**							
**Compd**.	***T*** **[K]**	**M1−P2**	**M1−P3**	**M1−C4**	**M1−C15**	**M1−C21**	**M1−C31**		
**1 a⋅CH_2_Cl_2_**	150	2.3648(7)	2.3437(7)	2.007(3)	2.000(3)	–	3.271(4)		
**2 a⋅CH_2_Cl_2_**	150	2.3513(9)	2.3421(9)	2.020(4)	2.012(4)	–	3.349(5)		
**1 b**.CH_2_Cl_2_	150	2.3755(7)	2.3636(7)	1.996(3)	1.994(3)	2.877(3)	2.899(3)		
**1 b**.CH_2_Cl_2_	25	2.3754(9)	2.3618(9)	1.999(4)	1.999(3)	2.854(4)	2.891(3)		
**1 b**.DFB	150	2.3758(6)	2.3617(5)	1.999(2)	1.993(2)	2.864(2)	2.877(2)		
**1 b**.DFB	25	2.3765(5)	2.3607(5)	2.0006(18)	1.9990(18)	2.8605(18)	2.8729(18)		
**2 b**.CH_2_Cl_2_	150	2.3614(7)	2.3608(7)	2.016(3)	2.010(3)	2.857(3)	2.875(3)		
**2 b**.CH_2_Cl_2_	25	2.3602(7)	2.3579(7)	2.014(3)	2.012(3)	2.837(3)	2.869(3)		
**2 b**.DFB	150	2.3651(6)	2.3581(6)	2.010(2)	2.016(2)	2.842(3)	2.856(2)		
**2 b**.DFB	25	2.3665(7)	2.3580(6)	2.015(3)	2.025(3)	2.844(3)	2.859(3)		
**1 c**	150	2.3593(7)	2.3542(7)	1.989(2)	1.995(2)	2.836(3)	3.185(3)		
**2 c**	150	2.352(2)	2.347(2)	2.021(7)	2.015(8)	2.810(8)	3.115(9)		
1 d*	150	2.3301(10)	2.3545(10)	1.992(4)	2.003(4)	2.863(5)	2.979(4)		
2 d*^[c]^	150	2.3301(15)	2.3501(15)	2.017(6)	2.024(6)	2.781(7)	2.956(6)		
		
		**Selected angles [°]**							
**Compd**.	***T*** **[K]**	**P2‐M1‐P3**	**C4‐M1‐C15**	**P2<npln^[b]^**	**C4‐M1‐P2**	**M1‐P2‐C20**	**P3<npln^[b]^**	**C15‐M1‐P3**	**M1‐P3‐C30**
**1 a⋅CH_2_Cl_2_**	150	172.05(2)	81.75(12)	–	–	–	4.24(7)	91.49(8)	104.26(10)
**2 a⋅CH_2_Cl_2_**	150	172.63(3)	81.34(16)	–	–	–	4.48(10)	92.45(10)	106.40(13)
**1 b**.CH_2_Cl_2_	150	170.82(2)	82.49(11)	8.04(6)	97.89(8)	97.05(9)	5.97(6)	95.89(8)	96.77(8)
**1 b**.CH_2_Cl_2_	25	170.80(3)	82.60(15)	8.07(8)	97.81(10)	96.82(11)	6.00(8)	95.91(10)	96.61(11)
**1 b**.DFB	150	170.68(2)	81.96(9)	9.74(5)	98.98(6)	96.00(7)	4.53(6)	94.82(6)	96.33(7)
**1 b**.DFB	25	170.419(17)	81.97(8)	10.31(4)	99.42(5)	95.94(6)	4.41(5)	94.61(5)	96.23(6)
**2 b**.CH_2_Cl_2_	150	169.91(2)	82.29(11)	8.88(6)	98.57(8)	97.22(9)	6.41(7)	96.59(8)	96.66(9)
**2 b**.CH_2_Cl_2_	25	169.92(2)	82.35(11)	8.88(6)	98.46(8)	96.99(8)	6.37(6)	96.64(8)	96.64(9)
**2 b**.DFB	150	169.87(2)	81.64(10)	10.55(5)	99.58(7)	96.15(8)	4.89(6)	95.33(7)	96.23(8)
**2 b**.DFB	25	169.71(3)	81.67(11)	10.93(5)	99.81(7)	96.37(8)	4.52(7)	94.86(7)	96.12(8)
**1 c**	150	172.64(2)	82.34(11)	9.00(6)	98.06(7)	96.35(9)	4.16(7)	94.06(7)	102.71(9)
**2 c**	150	171.10(7)	81.9(3)	10.35(17)	99.2(2)	96.3(3)	4.4(2)	94.5(2)	101.1(3)
1 d*	150	171.77(4)	82.03(18)	5.28(7)	92.69(11)	105.58(15)	6.61(11)	95.57(11)	104.93(14)
2 d*^[c]^	150	170.94(6)	81.7(3)	6.71(10)	93.47(17)	105.4(2)	6.28(16)	96.00(16)	106.3(2)

[a] Data for non‐disordered cations only. More extensive data provided in the Supporting Information. [b] Angle between the M1−P2/3 vector and the normal vector of the M1‐C4‐C9‐C10‐C15 (metallacycle) least squares plane. [c] Structure exhibits two independent and non‐disordered cations; data presented for the cation with equivalent conformation to that of 1 d*.

Dichloromethane is typically considered a weakly coordinating ligand and complexes of the platinum group metals are uncommon (<25 deposited in the Cambridge Structural Database v. 5.38). Indeed, **1 a⋅CH_2_Cl_2_** and **2 a⋅CH_2_Cl_2_** represent the first crystallographically characterised homologous metal series. Binding of the halocarbon in these complexes occurs with essentially linear Cl80‐M1‐C4 bond angles and M1−Cl80 bond lengths of 2.6067(8) and 2.5567(12) Å, for the rhodium and iridium variants, respectively. The latter are in line with Rh^III^ (2.488–2.763 Å)^21^ and Ir^III^ (2.533–2.612 Å)^22^ precedents and consistent with the stronger metal‐ligand bonding expected in the heavier congener. Although chelation of dichloromethane to platinum group metals is known,^23^ the remaining coordination site on the metal centre remains essentially vacant (M1⋅⋅⋅Cl82 >4 Å) without any significant stabilising (agostic or π) interaction with a phosphine substituent (M1⋅⋅⋅C31/C41 >3.2 Å). The phosphine ligands in closely related *trans*,*cis*‐[M(2,2′‐bipyridine)(PPh_3_)_2_H_2_]^+^ (M=Rh and Ir) are known to adopt a wide variety of conformations.^24^

Under the aforementioned experimental conditions the tricyclohexylphosphine complexes crystallise with one molecule of dichloromethane in the asymmetric unit, namely **1 b**.CH_2_Cl_2_ and **2 b**.CH_2_Cl_2_. In each case, the adoption of two significant γ‐agostic interactions with the metal centres is apparent in the solid‐state structures with M1−C21/C31 distances of 2.877(3)/2.899(3) and 2.857(3)/2.875(3) Å, for the rhodium and iridium congeners, respectively at 150 K. The close approaches of the C−H bonds to the metal centre are accompanied by significantly distorted phosphine geometries: C4‐M1‐P2/C15‐M1‐P3 angles greater than 90° (i.e. deviation from ideal metal coordination geometry) and compression of the M1‐P2‐C20/M1‐P3‐C30 angles compared to those of the other phosphine substituents (i.e. ligand yawing).

**Figure 4:**
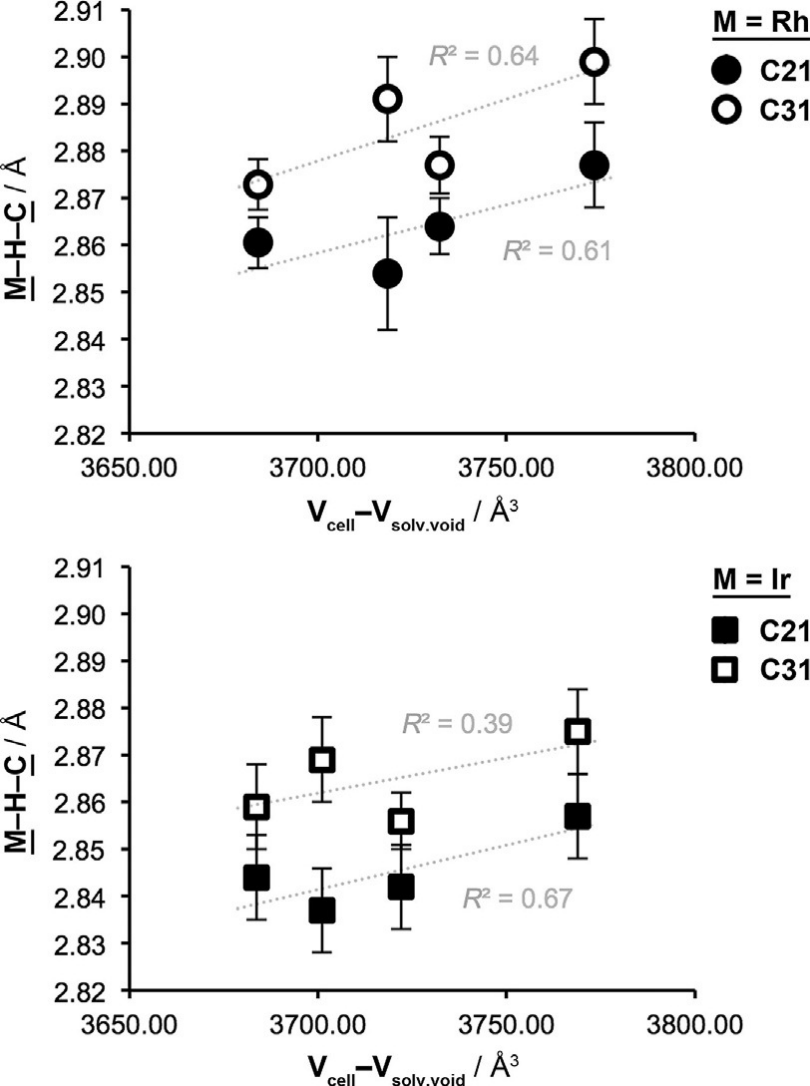
Bond length changes in **1 b** and **2 b** with molecular volume in the solid‐state.

The presence of a solvent molecule in the lattice presented an opportunity to explore the effect of crystal packing on the constituent agostic interactions. With this in mind, single crystals of **1 b** and **2 b** were also grown from weakly coordinating 1,2‐difluorobenzene (DFB)^25^ and pentane (liquid–liquid diffusion at RT) leading to inclusion of the fluoroarene into the lattice, namely **1 b**.DFB and **2 b**.DFB. The new crystals are isomorphic (*P*‐1), but, reflecting the larger solvent molecule, bear slightly larger unit cells (Δ*V*_cell_ ca. +2 %, Table 2). This enlargement does not directly parallel the associated increase solvent void volume (V_solv.void_), resulting in a small compression of the remaining unit cell contents at 150 K (Δ{V_cell_−V_solv.void_}=−1.1 %, **1 b**; −1.2 %, **2 b**) and, interestingly, shorter agostic interactions (Rh1−C21, −1.3±1.1 pm; Rh1−C31, −2.2±1.1 pm; Ir1−C21, −1.5±1.3 pm; Ir1−C31, −1.9±1.1 pm). Compression of **1 b** and **2 b** can also be achieved by cooling the crystalline samples from 150 to 25 K (Δ{V_cell_−V_solv.void_}=−1.5 %, **1 b**.CH_2_Cl_2_; −1.3 % **1 b**.DFB; −1.8 %, **2 b**.CH_2_Cl_2_; −1.0 %, **2 b**.DFB). Using this latter approach the most pronounced compression was achieved in the dichloromethane‐containing samples, where statistically significant contractions of the M1−C21 bond lengths are observed (−2.3±1.5 pm, **1 b**.CH_2_Cl_2_; −2.0±1.3 pm, **2 b**.CH_2_Cl_2_). Although the nature of the compression varies, analysis of the combined data reveals a noticeable correlation between molecular volume in the solid‐state and agostic bond length for the tricyclohexylphosphine complexes (Figure 4). Similar changes have been noted in a uranium complex using variable pressure X‐ray crystallography.^26^

**Table 2:** Selected cell properties and metrics for **1 b** and **2 b**.

Compd.	*T* [K]	V_cell_ [Å^3^]	ρ [g cm^−3^]	V_solv.void_ [Å^3^]^[a]^	V_solv.void_/V_cell_	M1−C21 [Å]	M1−C31 [Å]
**1 b**.CH_2_Cl_2_	150	4015.18(16)	1.459	241.84	6.0 %	2.877(3)	2.899(3)
**1 b**.CH_2_Cl_2_	25	3929.92(16)	1.491	211.50	5.4 %	2.854(4)	2.891(3)
**1 b**.DFB	150	4083.44(17)	1.458	351.21	8.6 %	2.864(2)	2.877(2)
**1 b**.DFB	25	4006.63(14)	1.486	322.48	8.0 %	2.8605(18)	2.8729(18)
**2 b**.CH_2_Cl_2_	150	4012.84(10)	1.534	244.00	6.1 %	2.857(3)	2.875(3)
**2 b**.CH_2_Cl_2_	25	3915.30(11)	1.572	214.31	5.5 %	2.837(3)	2.869(3)
**2 b**.DFB	150	4086.31(15)	1.530	364.19	8.9 %	2.842(3)	2.856(2)
**2 b**.DFB	25	4019.58(11)	1.555	335.86	8.4 %	2.844(3)	2.859(3)

[a] Solvent void calculated using the contact surface of the refined structure minus solvent (Mercury 3.9, probe radius of 1.2 Å and approximate grid spacing of 0.2 Å).

In contrast to the preceding structures, the X‐ray structures of **1 c** and **2 c** feature two crystallographically independent triisopropylphosphine ligated metal complexes, one of which is extensively disordered (see the Supporting Information for full details). The well‐ordered cations (Rh1/Ir1) are stabilised by one strong γ‐agostic interaction as evidenced through M1−C21 contacts <3 Å (2.836(3) Å, **1 c**; 2.810(8) Å, **2 c**), marked deviation of the associated phosphine (P2) from ideal coordination geometry (C4‐M1‐P2=98.06(7)°, **1 c**; 99.2(2)°, **2 c**), and the orientation of the associated substituent (M1‐P2‐C20=96.35(9)°, **1 c**; 96.3(3)°, **2 c**). A case can also be made for a weaker supplementary γ‐agostic from the other phosphine ligand (P3). The associated M1−C31 contacts are >3 Å (3.185(3) Å, **1 c**; 3.115(9) Å), **2 c**); however, distortion of the isopropyl groups towards the metal is discernable from the metrics (Table 1), albeit less pronounced than on P2 (and moreover the phosphines of **1 b** and **2 b**). The adoption of two agostic interactions of different magnitude is also evident in the disordered cations, although the nature of the disorder in these complexes precludes any meaningful analysis of the metrics: the remainder of the discussion is consequently focused only on the well‐ordered cations.

It is not immediately obvious why the agostic bonding pattern differs between complexes of PCy_3_ and P*i*Pr_3_. Close inspection of the phosphine ligands reveals equivalent conformations only for the ligands that adopt the strongest agostic interactions (P2); the non‐interacting substitutes of the other ligand (P3), however, differ by rotation about the P−C bonds.^27^ We speculate that the origin of these differences is ligand sterics: **1 c** and **2 c**, bearing the bulkier phosphine ligand (%V_bur_ @ 2.28 Å=32.3 vs. 31.8),^28^ are ultimately too congested to enable close approaches of two substituents. In the context of electronically stabilising the metal centre, this effect appears to be counterbalanced by shorter M1−C21 interactions in **1 c** and **2 c** (2.836(3) and 2.810(8) Å) compared to **1 b** and **2 b** (2.877(3) and 2.857(3) Å).

In the case of the triisobutylphosphine derivatives **1 d** and **2 d**, meaningful analysis in the solid‐state was impeded by extensive disorder of the phosphine ligands (see the Supporting Information for full details), necessitating alternative analysis of samples bearing instead the [Al{OC(CF_3_)_3_}_4_]^−^ counter anion; 1 d* and 2 d*. Although even in this case there are some subtle crystallographic differences between the rhodium and iridium congeners, these samples enable interrogation of nondisordered isostructural triisobutylphosphine complexes (1 d* shown in Figure 3, see the Supporting Information for full details). Contrasting the other trialkylphosphine variants, which feature γ‐agostic interactions, these complexes each show two δ‐agostic interactions, with M1−C21/C31 distances of 2.863(5)/2.979(4) and 2.781(7)/2.956(6) Å for the rhodium and iridium congeners, respectively. A difference easily reconciled when recognising the more flexible nature of the isobutyl substituent, which enables such interactions to be formed with significantly reduced distortion of the ligand. For instance, the triisobutylphosphine ligands in 1 d* and 2 d* are associated with distinctly more perpendicular C4‐M1‐P2/C15‐M1‐P3 and open M1‐P2‐C20/M1‐P3‐C30 angles than the other trialkylphosphine derivatives (Table 1).

Analysis of the metrics associated with the agostic interactions within the **1 b**–**d** and **2 b**–**d** homologous series enables an important general feature to be elucidated: more pronounced agostic interactions are formed in the iridium complexes, as evidenced through statistically shorter M1−C21/M1−C31 contacts of around 4 pm (Table 1). This assertion is reinforced through longer M1−C4/C15 distances (ca. 2 pm), associated with the *trans* disposed 2,2′‐biphenyl ligand (consistent with *trans* influence arguments), greater deviation of the C4‐M1‐P2/C15‐M1‐P3 angles from 90° (ca. 0.7°), and a less linear P2‐M1‐P3 bond angle (ca. 1.0°). Moreover, given that there are no statistically significant differences observed for the M1‐P2‐C20/M1‐P3‐C30 angles, it would appear that ligand yawing is a comparatively higher energy process than deviation from ideal metal‐phosphine coordination geometry, for iridium compared to rhodium. Within the data no meaningful correlation can be found between the M1−C21/C31 and M1−C4/C15 bond lengths, nor between the differences Δ(M1−C21, M1−C31) and Δ(M1−C4, M1−C15) calculated for each complex. This is perhaps not surprising when considering that variation of the M1−C4/C15 distances relative to the associated error is very low amongst the separate rhodium (1.989(2)–2.003(4) Å) and iridium (2.010(2)–2.024(6) Å) data sets collected at 150 K.

### Characterisation of 1 and 2 using NMR spectroscopy

**Figure 5:**
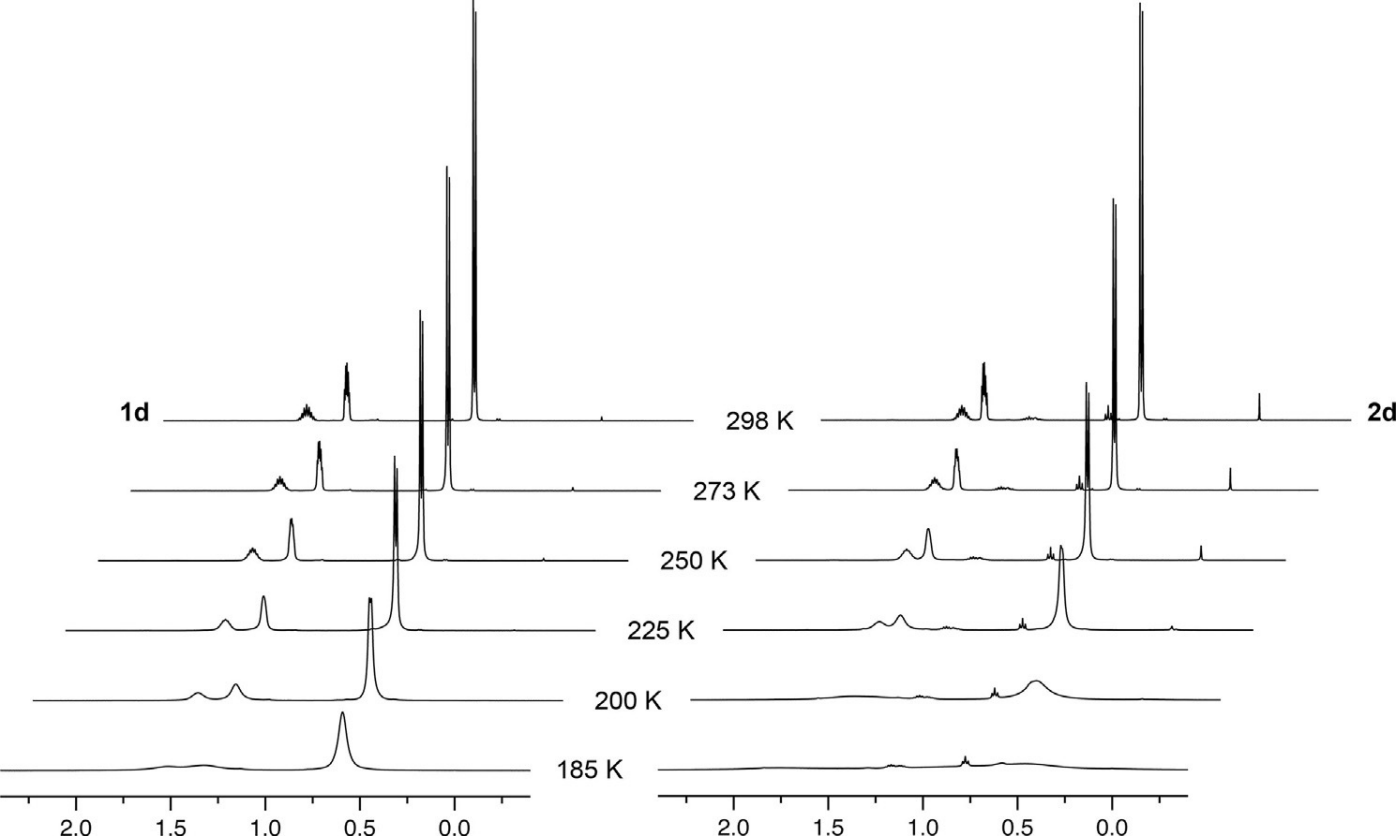
Variable temperature ^1^H NMR spectra of **1 d** and **2 d** (CD_2_Cl_2_, 500 MHz). Sample of **2 d** analysed contains trace quantities of pentane and grease.

The NMR spectra of **1 b**–**d** and **2 b**–**d** measured in CD_2_Cl_2_ solution at 298 K (500 MHz) are notable for the absence of low frequency ^1^H resonances^1^ and time averaged *C*_2*v*_ symmetry, indicating that persistent agostic interactions are not adopted under ambient conditions. This is perhaps not surprising given the inherently weak nature of 3‐centre‐2‐electron M−‐H−C bonds, associated distortion of the phosphine ligand from ideal coordination geometry, and capacity for fast exchange between substituents on the NMR time scale. In attempt to probe the latter, low temperature ^1^H (500 MHz) and ^31^P (202 MHz) NMR data were acquired in CD_2_Cl_2_, down to 185 K: the practical working limit for the solvent. Whilst in each case the onset of signal decoalescence was observed in the ^1^H spectra on cooling, in no instance was the slow exchange regime reached (see the Supporting Information). Consequently, conclusive interpretation and quantitative comparison of the variable temperature data was not possible. Nevertheless some general trends can be elucidated from qualitative inspection of the NMR data. For instance, as gauged though relative changes in the line broadening of the ^1^H signals, the onset of decoalescence occurs at noticeably higher temperatures for the iridium trialkylphosphine complexes **2 b**–**d** compared to the rhodium variants **1 b**–**d**. For the triisobutylphosphine derivatives, for example, appreciable line broadening is apparent in the ^1^H NMR spectrum of **2 d** on cooling from 298 to 225 K, whereas additional cooling to 200 K is required for similar changes in the spectrum of **1 d** (Figure 5). Reinforcing interpretation of the solid‐state data, this observation is consistent with stronger agostic interactions in the heavier group 9 congeners. In a similar manner analysis of the ^1^H NMR spectra indicates that more persistent M−H−C bonding is adopted in **1 d** and **2 d** than the other trialkylphosphine complexes and moreover in the relative order P*i*Bu_3_>PCy_3_>P*i*Pr. Although such a trend is not borne out in the observed M1−C21/C31 distances, negative correlations can be drawn out through the extent of phosphine distortion associated with forming a significant agostic interaction, that is, the P2<npln and C4‐M1‐P2 angles (Table 1).

As for the trialkylphosphine complexes, **1 a** and **2 a** display time averaged *C*_2*v*_ symmetry in CD_2_Cl_2_ solution at 298 K (500 MHz). For these complexes, however, partial decoalescence of the ^1^H signals of the phosphine substituents occurred on cooling to 185 K that we attribute to P−Ph restricted rotation of the phosphine ligands. The slow exchange regime is most advanced for **1 a** compared to **2 a** and at this temperature the phosphine ^31^P and the four 2,2′‐biphenyl ^1^H resonances remained sharp. The ^1^H NMR spectrum of **1 a** recorded at 185 K shows a significantly upfield shifted *ortho*‐phenyl 3H signal at *δ* 6.02 (fwhm=74 Hz) that exhibits a strong NOE interaction with the 6,6′‐biphenyl resonances indicating that they are pointing downwards towards the metal. Based on this data we suggest that coordination of the solvent is not significant under the range of temperatures we have studied, and instead there is a very weak bonding interaction between the phenyl ring of one of the phosphine ligands and the metal (time averaged across all the substituents). Such an interaction would explain why P−Ph restricted rotation is observed at low temperature by ^1^H NMR spectroscopy (large Δ*δ*_1H_), but not by ^31^P NMR spectroscopy (small Δ*δ*_31P_).

An alternative approach to gauge the degree of metal ligation in these homologous series could involve a chemical shift based‐scale employing the ^13^C resonances of the coordinated carbons of the 2,2′‐biphenyl ancillary ligand (*δ*_C_, Table 3) that are *trans* to the “free” coordination sites. Similar approaches employing the ^13^C resonances of *trans*‐disposed NHC ligands^29^ or metal‐carbides^30^ as ligand electronic parameters have been used to excellent effect. For **1** and **2**, absolute values of *δ*_C_ cannot be used due to non‐negligible contributions from the different *cis* phosphine ligands.^31^ We have attempted to deconvolute such contributions by using the chemical shift difference between **1** and **2** and their respective precursors **3** and **4**, however, it is not possible to draw a conclusive trend for all the phosphine ligands studied (Table 3). The smallest differences, however, are observed for the triisobutylphosphine derivatives, consistent with the adoption of the strongest agostic interactions.

**Table 3:** Selected NMR data for **1** and **2** (CD_2_Cl_2_, 298 K).

Compd.	Sym.	*δ*_P_ (^1^ *J*_RhP_)	Δ*δ*_P_ (Δ^1^ *J*_RhC_)^[a]^	*δ*_C_ (^1^ *J*_RhC_)	Δ*δ*_C_ (Δ^1^ *J*_RhC_)^[a]^
**1 a**	*C*_2*v*_	19.7 (118 Hz)	−9.0 (−1 Hz)	154.8 (39 Hz)	−8.9 (+6 Hz)
**2 a**	*C*_2*v*_	11.6	−10.1	127.0	−11.4
**1 b**	*C*_2*v*_	13.4 (109 Hz)	−0.4 (+1 Hz)	153.8 (44 Hz)	−8.6 (+7 Hz)
**2 b**	*C*_2*v*_	3.0	+8.4	125.8	−11.4
**1 c**	*C*_2*v*_	25.7 (112 Hz)	+2.7 (+3 Hz)	152.1 (44 Hz)	−8.6 (+9 Hz)
**2 c**	*C*_2*v*_	17.8	+12.0	123.4	−12.4
**1 d**	*C*_2*v*_	18.5 (110 Hz)	+5.6 (+1 Hz)	156.6 (43 Hz)	−6.7 (+7 Hz)
**2 d**	*C*_2*v*_	14.5	+13.9	130.3	−7.9

[a] Change in parameter relative to that measured in **3** (M=Rh) or **4** (M=Ir).

### Computational insights

**Figure 6:**
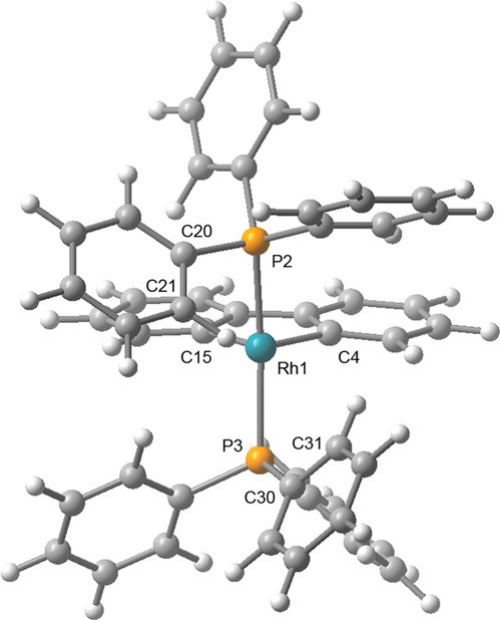
Optimised structure of **1 a′**. Selected bond lengths (Å) and angles (°) for **1 a′**: Rh1−P2, 2.3413; Rh1−P3, 2.3482; Rh1−C4, 1.977; Rh1−C15, 1.969; Rh1−C21, 3.183; Rh1−C31, 3.272; P2‐Rh1‐P3, 166.34; C4‐Rh1‐C15, 82.73; P2<npln, 5.89; C4‐Rh1‐P2, 97.00; Rh1‐P2‐C20, 94.85; P3<npln, 8.00; C15‐Rh1‐P3, 94.58; Rh1‐P3‐C30, 101.30. Equivalent metrics for **2 a′**: Ir1−P2, 2.3432; Ir1−P3, 2.3423; Ir1−C4, 1.993; Ir1−C15, 1.988; Ir1−C21, 3.243; Ir1−C31, 3.245; P2‐Ir1‐P3, 164.75; C4‐Ir1‐C15, 82.61; P2<npln, 7.20; C4‐Ir1‐P2, 98.45; Ir1‐P2‐C20, 96.08; P3<npln, 8.59; C15‐Ir1‐P3, 95.56; Ir1‐P3‐C30, 101.90.

Supplementing the experimental findings, the structures of low‐coordinate complexes **1** and **2** have been examined in silico using DFT‐based calculations at the pbe0/def2‐tzvp level of theory.^32^ In accord with the preceding analysis, structures of the associated cations were optimised starting from geometries of only the well‐ordered/major disordered components of cations observed in the solid‐state; **1′** and **2′**. In the case of the triphenylphosphine adducts, structures of both low‐coordinate **1 a′** and **2 a′** (**1 a′** depicted in Figure 6) and dichloromethane complexes **1 a′⋅CH_2_Cl_2_** and **2 a′⋅CH_2_Cl_2_** were interrogated. The binding of dichloromethane to low‐coordinate **1 a′** and **2 a′** is calculated to be weak (Δ*H*=−5.18/−5.96 kcal mol^−1^) and ultimately formation of **1 a′⋅CH_2_Cl_2_** and **2 a′⋅CH_2_Cl_2_** are predicted to be significantly endoergic at 298 K (Δ*G*_298K_=+9.20/+9.34 kcal mol^−1^). These data therefore imply retention of the halocarbon would be entropically disfavoured in solution,^33^ reconciling the experimental evidence.

**Figure 7:**
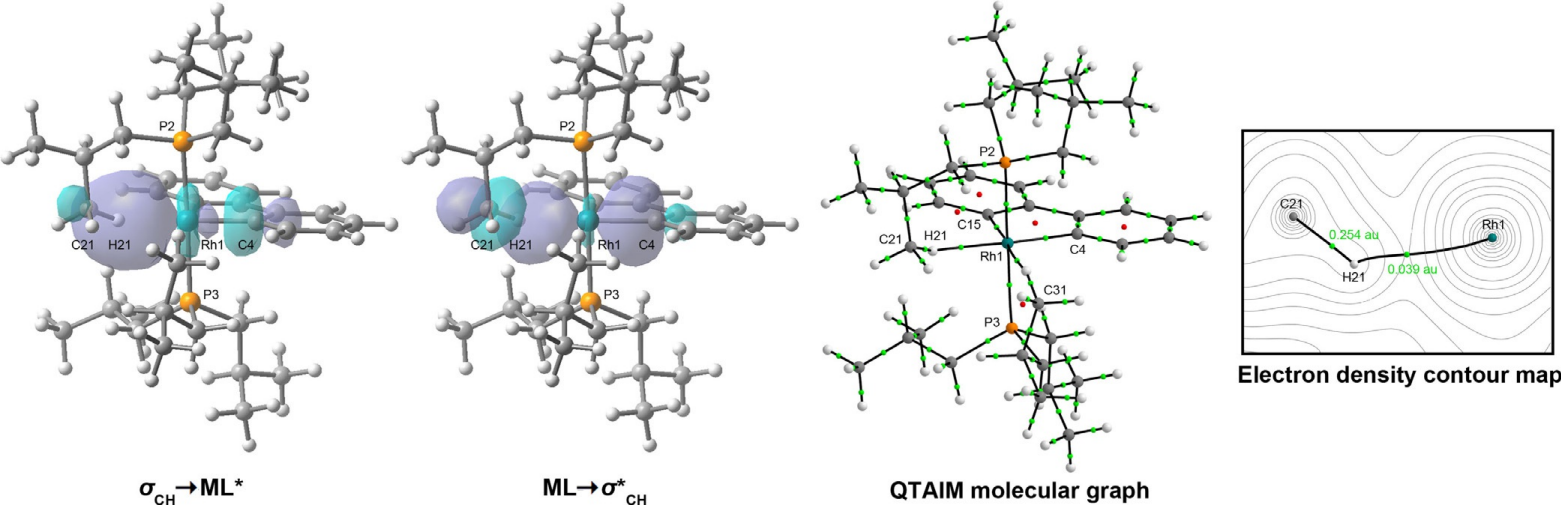
Key NBO orbital overlaps associated with the major agostic interaction in **1 d′**, QTAIM molecular graph of **1 d′** (showing bond paths, bond critical points and ring critical points), and calculated electron density topology associated with the major agostic interaction in **1 d′** (showing bond paths and electron density at bond critical points).

The presence of agostic interactions in the trialkylphosphine complexes was fully corroborated by analysis of **1 b**–**d′** and **2 b**–**d′** using both the Natural Bond Orbital (NBO) and Quantum Theory of Atoms in Molecules (QTAIM) approaches (Figure 7, Table 4).^34^ Using the former, adoption of 3‐centre‐2‐electron M−H−C bonds is evidenced through significant perturbation energies associated with σ_CH_→ML* and ML→σ*_CH_ interactions (21.81–63.64 kcal mol^−1^), while examination of the electron density using the latter reveals characteristic curved bond paths between the metal centre and hydrogen atom and associated critical point properties (*ρ*_MH_=0.017–0.051; ∇^2^ *ρ*_MH_=+0.049–+0.167).^35^ Moreover, using the more intuitive bond delocalisation parameter, significant M−H and correspondingly reduced C−H “bond orders” are apparent from the QTAIM analysis.

**Table 4:** Selected NBO and QTAIM data for **1′** and **2′**.

	**NBO perturbation energy^[a]^ [kcal mol^−1^]**							
**Cmpd**.	**Major agostic^[b]^**		**Minor agostic^[b]^**		**Sum**			
	σ_CH_→ML*	ML→σ*_CH_	σ_CH_→ML*	ML→σ*_CH_				
**1 a′**	1.07	0.50	1.16	<0.05	2.73			
**2 a′**	1.65	0.64	1.64	1.72	5.65			
**1 b′**	8.10	3.61	6.12	3.98	21.81			
**2 b′**	10.65	6.09	10.05	6.78	33.57			
**1 c′**	8.94	2.93	1.95	1.90	15.72			
**2 c′**	11.75	3.38	3.30	3.56	21.99			
**1 d′**	14.91	7.18	11.57	9.47	43.13			
**2 d′**	19.19	10.81	16.94	16.70	63.64			
								
	**QTAIM bond critical point properties**							
**Cmpd**.	**Major agostic^[b]^**				**Minor agostic^[b]^**			
	***ρ*_MH_**	**∇^2^*ρ*_MH_**	***ρ*_CH_**	**∇^2^*ρ*_CH_**	***ρ*_MH_**	**∇^2^*ρ*_MH_**	***ρ*_CH_**	**∇^2^*ρ*_CH_**
**1 a′**	–^[d]^	–^[d]^	0.282	−0.994	–^[d]^	–^[d]^	0.282	−0.991
**2 a′**	–^[d]^	–^[d]^	0.283	−0.998	–^[d]^	–^[d]^	0.280	−0.978
**1 b′**	0.027	+0.102	0.261	−0.847	0.024	+0.081	0.263	−0.856
**2 b′**	0.034	+0.118	0.254	−0.802	0.033	+0.113	0.254	−0.800
**1 c′**	0.027	+0.107	0.261	−0.846	–^[d]^	–^[d]^	0.270	−0.898
**2 c′**	0.035	+0.130	0.255	−0.805	0.017	+0.049	0.267	−0.878
**1 d′**	0.039	+0.158	0.254	−0.795	0.032	+0.124	0.256	−0.807
**2 d′**	0.051	+0.167	0.246	−0.749	0.041	+0.139	0.249	−0.766
								
	**QTAIM delocalisation index**							
**Cmpd**.	**Major agostic^[b]^**		**Minor agostic^[b]^**		**Ref.^[c]^**	**Sum**		
	**M−H**	**C−H**	**M−H**	**C−H**	**C−H**	**@M**		
**1 a′**	–	0.925	–	0.920	0.923(4)	3.664		
**2 a′**	–	0.924	–	0.913	0.924(4)	3.997		
**1 b′**	0.117	0.858	0.105	0.866	0.914(5)	3.838		
**2 b′**	0.160	0.832	0.157	0.837	0.914(5)	4.236		
**1 c′**	0.114	0.876	–	0.920	0.937(7)	3.767		
**2 c′**	0.156	0.850	0.069	0.906	0.937(7)	4.186		
**1 d′**	0.181	0.842	0.160	0.861	0.948(5)	3.957		
**2 d′**	0.234	0.807	0.207	0.833	0.948(5)	4.349		

[a] Resulting from interactions of σ_CH_ and σ*_CH_ orbitals with the metal 2,2′‐biphenyl σ_MC_ and σ*_MC_ orbitals. [b] Assignment based on M−C bond length, for example, Rh1−HC21 (major) and Rh1−HC31 (minor) in Figures 3 and 6. [c] Average values of equivalent non‐agostic C−H bonds (standard deviation). [d] Bond critical point not located.

The associated metrics help quantify previous trends elucidated from the experimental work: significantly stronger agostic interactions are adopted in the iridium congeners, with around 40 % larger NBO donor‐acceptor energies and QTAIM M−H delocalisation indices, and the degree of agostic bonding decreases in the order, P*i*Bu_3_≫PCy_3_>P*i*Pr_3_ (notably for **1 c′** only one agostic interaction is detected in the QTAIM analysis).

Although the optimised structures of **1 a′** and **2 a′** show significant distortion of the phosphine substituents towards the metal, only very weak agostic interactions are inferred from the NBO analysis with the perturbation energies associated with σ_CH_→ML* and ML→σ*_CH_ interactions <6 kcal mol^−1^ (cf. >15 kcal mol^−1^ for the alkyl phosphine complexes). Moreover, inspection of the donor‐acceptor NBO interactions associated with the phosphine substituents proximate to the metal centre show no significant π‐interactions. No bond paths between the metal centre and associated hydrogen atoms were detected in the QTAIM analysis. The data are therefore consistent with very low‐coordinate complexes. Indeed, the metal centres in these complexes have the lowest sum of delocalisation indices for each respective metal series (3.664, **1 a′**; 3.997, **2 a′**).

## Conclusion

A homologous family of low‐coordinate complexes of the formulation *trans*‐[M(2,2′‐biphenyl)(PR_3_)_2_][BAr^F^_4_] (M=Rh, **1**; Ir, **2**; R=Ph, **a**; Cy, **b**; *i*Pr, **c**; *i*Bu, **d**) has been prepared and extensively structurally characterised. The formation of these sawhorse complexes is promoted through incorporation of the high *trans* influence 2,2′‐biphenyl ancillary ligand and stabilised through the adoption of weak agostic interactions, at the opposing open coordination sites, between the phosphine ligand substituents and the metal centres.

Enabled through a comprehensive set of solution phase (VT ^1^H and ^31^P NMR spectroscopy) and solid‐state (single crystal X‐ray diffraction) experimental data, and analysis in silico (DFT‐based NBO and QTAIM analysis), the structural features of the constituent agostic interactions have been systematically interrogated. The combined data substantiates the adoption of stronger agostic interactions for the Ir^III^ compared to Rh^III^ complexes and, with respect to the phosphine ligands, in the order P*i*Bu_3_>PCy_3_>P*i*Pr_3_>PPh_3_.

In contrast to the trialkylphosphine complexes which feature notable M−H−C bonds, the triphenylphosphine variants are instead only obtained in the solid‐state as adducts of the weakly coordinating solvent dichloromethane employed; **1 a⋅CH_2_Cl_2_** and **2 a⋅CH_2_Cl_2_**. The entropically unstable nature of these adducts was, however, evidenced in solution by ^1^H and ^31^P NMR spectroscopy and is supported by DFT calculations. Moreover, NBO and QTAIM analysis of optimised structures of **1 a** and **2 a** highlight the insubstantial nature of M−H−C bonds in these low‐coordinate complexes. The formation of the strongest agostic interactions observed in triisobutylphosphine derivatives is attributed to the flexible nature of the isobutyl substituents, and associated with Rh−C and Ir−C distances of 2.863(5)/2.979(4) and 2.781(7)/2.956(6) Å, respectively, in the solid‐state and reduced structural dynamics in solution. For these complexes, extensive σ_CH_→ML* and ML→σ*_CH_ interactions are apparent in the NBO perturbation analysis (**1 d′**, 43.13; **2 d′**, 63.64 kcal mol^−1^) and significant QTAIM M−H bond delocalication indices are calculated (**1 d′**, 0.181/0.160; **2 d′**, 0.234/0.207).

In addition to the above structure–property relationships, the effect of crystal packing on agostic interactions was investigated in **1 b** and **2 b**. Compression of the associated cations, through inclusion of a more bulky solvent molecule (1,2‐difluorobenzene vs. CH_2_Cl_2_) in the lattice or collection of data at very low temperature (25 K vs. 150 K), lead to small but statistically significant shortening of the M−H−C distances.

## Experimental Section

### General synthetic methods

All manipulations were performed under an atmosphere of argon using Schlenk and glove box techniques. Glassware was oven‐dried at 150 °C overnight and flamed under vacuum prior to use. CH_2_Cl_2_, CD_2_Cl_2_ and 1,2‐difluorobenzene were dried over CaH_2_, vacuum distilled, and then stored over thoroughly vacuum‐dried 3 Å molecular sieves. Pentane was dried over Na/K alloy, vacuum distilled, and then stored over thoroughly vacuum‐dried 3 Å molecular sieves. [Rh(2,2′‐biphenyl)(dtbpm)Cl] **5**,^16^ [Ir(2,2′‐biphenyl)(COD)Cl]_2_ **6**,^17^ and Na[BAr^F^_4_]^36^ were synthesised using literature protocols. *trans*‐[Rh(2,2′‐biphenyl)(PPh_3_)_2_Cl] **3 a** 16 and *trans*‐[Ir(2,2′‐biphenyl)(PPh_3_)_2_Cl] **4 a** 17 were prepared using slightly adapted literature procedures that are described below for completeness. All other solvents and reagents are commercial products and were used as received. NMR spectra were recorded on Bruker DPX, AV and HD spectrometers at 298 K unless otherwise stated. Variable temperature data was collected on a Bruker AV 500 MHz spectrometer. Low‐resolution electrospray ionisation mass spectra (LR ESI‐MS) were recorded on an Agilent 6130B single Quad spectrometer. High‐resolution electrospray ionisation mass spectra (HR ESI‐MS) were recorded on a Bruker MaXis II spectrometer. Microanalyses were performed by Stephen Boyer at London Metropolitan University.

### Synthesis of *trans*‐[M(2,2′‐biphenyl)(PR_3_)_2_Cl] (M=Rh, 3; Ir, 4)

**3 a**: A solution of **5** (30.0 mg, 50.4 μmol) and PPh_3_ (28.4 mg, 108 μmol) in CH_2_Cl_2_ (5 mL) was stirred at RT for 3 hours. The product was precipitated by addition of excess Et_2_O (ca. 20 mL) and isolated by filtration. Yield: 25.1 mg (83 %, microcrystalline yellow solid). Spectroscopic data is fully consistent with previously reported values.^16^

^**1**^ **H NMR** (500 MHz, CD_2_Cl_2_): *δ*=7.42 (d, ^3^ *J*_HH_=7.8 Hz, 2 H, biph), 7.32 (t, ^3^ *J*_HH_=7.4 Hz, 6 H, Ph), 7.27 (br, fwhm=30 Hz, 12 H, Ph), 7.17 (t, ^3^ *J*_HH_=7.6 Hz, 12 H, Ph), 6.57 (t, ^3^ *J*_HH_=7.3 Hz, 2 H, biph), 6.45 (td, ^3^ *J*_HH_=7.5 Hz, ^4^ *J*_HH_=1.6 Hz, 2 H, biph), 6.34 (dd, ^3^ *J*_HH_=7.5 Hz, ^4^ *J*_HH_=1.6 Hz, 2 H, biph). ^**13**^ **C{^1^H} NMR** (126 MHz, CD_2_Cl_2_): *δ*=163.7 (dt, ^1^ *J*_RhC_=33 Hz, ^2^ *J*_PC_=10 Hz), 153.9 (s, biph), 135.0 (t, *J*_PC_=5 Hz, Ph), 133.1 (s, biph), 130.7 (t, *J*_PC_=23 Hz, Ph), 130.4 (s, Ph), 128.2 (t, *J*_PC_=5 Hz, Ph), 123.8 (s, biph), 122.9 (s, biph), 122.1 (s, biph). ^**31**^ **P{^1^H} NMR** (162 MHz, CD_2_Cl_2_): *δ*=28.7 (d, ^1^ *J*_RhP_=119 Hz). **LR ESI‐MS** (positive ion): 779.1 ([*M*−Cl]^+^, calcd 779.1) *m*/*z*.

**3 b**: A solution of **5** (50.0 mg, 84.0 μmol) and PCy_3_ (47.4 mg, 169 μmol) in CH_2_Cl_2_ (5 mL) was stirred at RT for 16 hours. The resulting precipitate was filtered and washed with cold CH_2_Cl_2_ (3×5 mL). Yield: 59.3 mg (83 %, yellow solid).

^**1**^ **H NMR** (500 MHz, CD_2_Cl_2_): *δ*=7.70 (d, ^3^ *J*_HH_=8.0 Hz, 2 H, biph), 7.32 (dd, ^3^ *J*_HH_=7.6 Hz, ^4^ *J*_HH_=1.6 Hz, 2 H, biph), 6.92 (t, ^3^ *J*_HH_=7.3 Hz, 2 H, biph), 6.76 (dt, ^3^ *J*_HH_=7.5 Hz, ^4^ *J*_HH_=1.6 Hz, 2 H, biph), 2.03 (app. t, *J=*12 Hz, 6 H, Cy), 1.53–1.66 (m, 30 H, Cy), 1.26 (app. q, *J=*12 Hz, 14 H, Cy), 0.99–1.18 (m, 16 H, Cy). ^**13**^ **C{^1^H} NMR** (126 MHz, CD_2_Cl_2_): *δ*=162.4 (dt, ^1^ *J*_RhC_=37 Hz, ^2^ *J*_PC_=9 Hz, biph), 153.2 (s, biph), 137.2 (s, biph), 124.6 (s, biph), 122.6 (s, biph), 120.2 (s, biph), 35.3 (t, *J*_PC_=9 Hz, Cy), 30.6 (Cy), 28.4 (t, *J*_PC_=5 Hz, Cy), 26.9 (Cy). ^**31**^ **P{^1^H} NMR** (121 MHz, CD_2_Cl_2_): *δ*=13.8 (d, ^1^ *J*_RhP_=108 Hz). **HR ESI‐MS** (positive ion): 815.4309 ([*M*−Cl]^+^, calcd 815.4315) *m*/*z*. Despite repeated attempts we have been unable to obtain satisfactory microanalytical data for this compound.

**3 c**: To a solution of **5** (17.8 mg, 30.0 μmol) in CH_2_Cl_2_ (5 mL) was added P*i*Pr_3_ (0.84 m in pentane, 71.8 μL, 60.3 μmol) and the resulting solution stirred at RT for 3 hours. The volatiles were removed in vacuo and the residue extracted with CH_2_Cl_2_ (5 mL) through a short plug of neutral Al_2_O_3_. The solvent was then removed in vacuo to afford the pure product. Yield: 6.8 mg (37 %, yellow solid).

^**1**^ **H NMR** (500 MHz, CD_2_Cl_2_): *δ*=7.77 (d, ^3^ *J*_HH_=7.9 Hz, 2 H, biph), 7.30 (dd, ^3^ *J*_HH_=7.5 Hz, ^4^ *J*_HH_=1.6 Hz, 2 H, biph), 6.94 (t, ^3^ *J*_HH_=7.3 Hz, 2 H, biph), 6.76 (td, ^3^ *J*_HH_=7.6 Hz, ^4^ *J*_HH_=1.6 Hz, 2 H, biph), 2.36–2.44 (m, 6 H, CH(CH_3_)_2_), 0.98 (app. q, *J=*7 Hz, 36 H, CH_3_). ^**13**^ **C{^1^H} NMR** (126 MHz, CD_2_Cl_2_): *δ*=160.7 (dt, ^1^ *J*_RhC_=35 Hz, ^2^ *J*_PC_=9 Hz, biph), 154.4 (s, biph), 136.8 (s, biph), 124.7 (s, biph), 123.1 (s, biph), 120.3 (s, biph), 24.1 (t, *J*_PC_=10 Hz, CH(CH_3_)_2_), 20.3 (s, CH_3_). ^**31**^ **P{^1^H} NMR** (121 MHz, CD_2_Cl_2_): *δ*=23.0 (d, ^1^ *J*_RhP_=109 Hz). **HR ESI‐MS** (positive ion): 575.2436 ([*M*−Cl]^+^, calcd 575.2437) *m*/*z*. **Anal**. Calcd for C_30_H_50_ClP_2_Rh (611.03 g mol^−1^): C, 58.97; H, 8.25; N, 0.00. Found: C, 58.82; H, 8.09; N, 0.00.

**3 d**: To a solution of **5** (17.8 mg, 30.0 μmol) in CH_2_Cl_2_ (5 mL) was added P*i*Bu_3_ (15.1 μL, 60.3 μmol) and the resulting solution stirred at RT for 3 hours. The volatiles were removed in vacuo and the residue extracted with CH_2_Cl_2_ (5 mL) through a short plug of neutral Al_2_O_3_. The solvent was then removed in vacuo to afford the pure product. Yield: 11.6 mg (56 %, yellow solid).

^**1**^ **H NMR** (500 MHz, CD_2_Cl_2_): *δ*=7.53 (d, ^3^ *J*_HH_=7.8 Hz, 2 H, biph), 7.34 (dd, ^3^ *J*_HH_=7.5 Hz, ^4^ *J*_HH_=1.6 Hz, 2 H, biph), 6.96 (t, ^3^ *J*_HH_=7.3 Hz, 2 H, biph), 6.79 (td, ^3^ *J*_HH_=7.5 Hz, ^4^ *J*_HH_=1.6 Hz, 2 H, biph), 1.75–1.87 (m, 6 H, CH_2_CH), 1.43 (app. dt, *J=*6 Hz, *J=*3 Hz, 12 H, CH_2_), 0.78 (d, ^3^ *J*_HH_=6.7 Hz, 36 H, CH_3_). ^**13**^ **C{^1^H} NMR** (126 MHz, CD_2_Cl_2_): *δ*=163.3 (dt, ^1^ *J*_RhC_=36 Hz, ^2^ *J*_PC_=10 Hz, biph), 152.6 (s, biph), 134.5 (s, biph), 125.4 (s, biph), 122.9 (s, biph), 120.7 (s, biph), 32.6 (t, *J*_PC_=11 Hz, CH_2_), 26.1 (t, *J*_PC_=3 Hz, CH_2_ CH), 25.1 (s, CH_3_). ^**31**^ **P{^1^H} NMR** (162 MHz, CD_2_Cl_2_): *δ=*12.9 (d, ^1^ *J*_RhP_=109 Hz). **HR ESI‐MS** (positive ion): 659.3378 ([*M*−Cl]^+^, calc. 659.3376) *m*/*z*. **Anal**. Calcd for C_36_H_62_ClP_2_Rh (695.20 g mol^−1^): C, 62.20; H, 8.99; N, 0.00. Found: C, 61.89; H, 8.84; N, 0.00.

**4 a**: A solution of **6** (50.0 mg, 51.2 μmol) and PPh_3_ (54.0 mg, 206 μmol) in CH_2_Cl_2_ (5 mL) was stirred at RT for 18 hours. The product was precipitated by addition of excess Et_2_O (ca. 20 mL) and isolated by filtration. Yield: 64.1 mg (83 %, microcrystalline yellow solid). Spectroscopic data is fully consistent with previously reported values.^17^

^**1**^ **H NMR** (500 MHz, CD_2_Cl_2_): *δ*=7.34 (d, ^3^ *J*_HH_=8 Hz, 2 H, biph), 7.32 (t, ^3^ *J*_HH_=7.5 Hz, 6 H, Ph), 7.25 (br, fwhm=40 Hz, 12 H, Ph), 7.18 (t, ^3^ *J*_HH_=7.5 Hz, 12 H, Ph), 6.47 (t, ^3^ *J*_HH_=7.3 Hz, 2 H, biph), 6.28 (td, ^3^ *J*_HH_=7.6 Hz, ^4^ *J*_HH_=1.6 Hz, 2 H, biph), 6.26 (dd, ^3^ *J*_HH_=7.5 Hz, ^4^ *J*_HH_=1.6 Hz, 2 H, biph). ^**13**^ **C{^1^H} NMR** (126 MHz, CD_2_Cl_2_): *δ=*155.6 (s, biph), 138.4 (t, ^2^ *J*_PC_=7 Hz, biph), 135.1 (t, *J*_PC_=5 Hz, Ph), 132.8 (t, ^3^ *J*_PC_=2 Hz, biph), 130.4 (s, Ph), 130.1 (t, *J*_PC_=27 Hz, Ph), 128.2 (t, *J*_PC_=5 Hz, Ph), 123.8 (s, biph), 122.3 (s, biph), 121.4 (s, biph). ^**31**^ **P{^1^H} NMR** (162 MHz, CD_2_Cl_2_): *δ*=21.7 (s). **LR ESI‐MS** (positive ion): 869.2 ([*M*−Cl]^+^, calcd 869.2) *m*/*z*.

**4 b**: A solution of **6** (60.0 mg, 61.5 μmol) and PCy_3_ (69.3 mg, 247 μmol) in CH_2_Cl_2_ (5 mL) was stirred at RT for 16 hours. The resulting precipitate was filtered and washed with CH_2_Cl_2_ (3×5 mL). Yield: 93.0 mg (80 %, orange solid).

^**1**^ **H NMR** (500 MHz, CD_2_Cl_2_): *δ*=7.53 (d, ^3^ *J*_HH_=7.8 Hz, 2 H, biph), 7.25 (dd, ^3^ *J*_HH_=7.6 Hz, ^4^ *J*_HH_=1.5 Hz, 2 H, biph), 6.83 (t, ^3^ *J*_HH_=7.3 Hz, 2 H, biph), 6.64 (td, ^3^ *J*_HH_=7.3 Hz, ^4^ *J*_HH_=1.5 Hz, 2 H, biph), 2.15 (app. t, *J=*12 Hz, 6 H, Cy), 1.56–1.69 (m, 18 H, Cy), 1.56–1.42 (m, 12 H, Cy), 1.27 (app. q, *J=*12 Hz, 12 H, Cy), 1.18–0.97 (m, 18 H, Cy). ^**13**^ **C{^1^H} NMR** (126 MHz, CD_2_Cl_2_): *δ=*154.4 (s, biph), 137.2 (t, ^2^ *J*_PC_=7 Hz, biph), 135.9 (s, biph), 124.8 (s, biph), 121.9 (s, biph), 119.8 (s, biph), 35.0 (t, *J*_PC_=12 Hz, Cy), 30.6 (s, Cy), 28.4 (t, *J*_PC_=5 Hz, Cy), 26.9 (s, Cy). ^**31**^ **P{^1^H} NMR** (162 MHz, CD_2_Cl_2_): *δ=*−5.4 (s). **HR ESI‐MS** (positive ion): 905.4904 ([*M*−Cl]^+^, calcd 905.4893) *m*/*z*. **Anal**. Calcd for C_48_H_74_ClIrP_2_ (940.73 g mol^−1^): C, 61.28; H, 7.93; N, 0.00. Found: C, 61.17; H, 8.01; N, 0.00.

**4 c**: To a solution of **6** (60.0 mg, 61.5 μmol) in CH_2_Cl_2_ (5 mL) was added P*i*Pr_3_ (0.84 m in pentane, 293 μL, 247 μmol) and the resulting solution stirred at RT for 16 hours. The solution was concentrated to ca. 2 mL, diluted with pentane (5 mL), and then filtered. The filtrate was dried in vacuo and the residues washed with pentane (2 mL) at −78 °C to afford the pure product. Yield: 59.0 mg (69 %, orange solid).

^**1**^ **H NMR** (500 MHz, CD_2_Cl_2_): *δ*=7.62 (dd, ^3^ *J*_HH_=7.9 Hz, 2 H, biph), 7.23 (dd, ^3^ *J*_HH_=7.5 Hz, ^4^ *J*_HH_=1.6 Hz, 2 H, biph), 6.84 (t, ^3^ *J*_HH_=7.3 Hz, 2 H, biph), 6.61 (td, ^3^ *J*_HH_=7.5 Hz, ^4^ *J*_HH_=1.6 Hz, biph), 2.48–2.56 (m, 6 H, CH(CH_3_)_2_), 0.98 (app. q, *J=*7 Hz, 36 H, CH_3_). ^**13**^ **C{^1^H} NMR** (126 MHz, CD_2_Cl_2_): *δ*=155.8 (s, biph), 135.8 (t, ^2^ *J*_PC_=7 Hz, biph), 135.7 (s, biph), 124.7 (s, biph), 122.5 (s, biph), 120.0 (s, biph), 23.7 (t, *J*_PC_=13 Hz, CH(CH_3_)_2_), 20.3 (s, CH_3_). ^**31**^ **P{^1^H} NMR** (121 MHz, CD_2_Cl_2_): *δ*=5.8 (s). **HR ESI‐MS** (positive ion): 665.3012 ([*M*−Cl]^+^, calcd 665.3013) *m*/*z*. **Anal**. Calcd for C_30_H_50_ClIrP_2_ (700.34 g mol^−1^): C, 51.45; H, 7.20; N, 0.00. Found: C, 51.45; H, 7.39; N, 0.00.

**4 d**: To a solution of **6** (60.0 mg, 61.5 μmol) in CH_2_Cl_2_ (5 mL) was added P*i*Bu_3_ (61.6 μL, 247 μmol) and the resulting solution stirred at RT for 16 hours. The solution was concentrated to ca. 2 mL, diluted with pentane (5 mL), and then filtered. The solvent was then removed in vacuo to afford the pure product. Yield=78.0 mg (81 %, orange solid).

^**1**^ **H NMR** (500 MHz, CD_2_Cl_2_): *δ*=7.43 (d, ^3^ *J*_HH_=7.7 Hz, 2 H, biph), 7.27 (dd, ^3^ *J*_HH_=7.5 Hz, ^4^ *J*_HH_=1.5 Hz, 2 H, biph), 6.88 (t, ^3^ *J*_HH_=7.3 Hz, 2 H, biph), 6.66 (td, ^3^ *J*_HH_=7.6 Hz, ^4^ *J*_HH_=1.5 Hz, 2 H, biph), 1.75–1.85 (m, 6 H CH_2_CH), 1.52 (app. dt, *J=*6 Hz, *J=*3, 12 H, CH_2_), 0.76 (d, ^3^ *J*_HH_=6.7 Hz, 36 H, CH_3_). ^**13**^ **C{^1^H} NMR** (126 MHz, CD_2_Cl_2_): *δ*=154.1 (s, biph), 138.2 (t, ^2^ *J*_PC_=7 Hz, biph), 134.1 (s, biph), 125.5 (s, biph), 122.3 (s, biph), 120.2 (s, biph), 32.1 (t, *J*_PC_=14 Hz, CH_2_), 26.1 (t, *J*_PC_=4 Hz, CH_2_ CH), 25.0 (s, CH_3_). ^**31**^ **P{^1^H} NMR** (202 MHz, CD_2_Cl_2_): *δ*=0.6 (s). **HR ESI‐MS** (positive ion): 749.3954 ([*M*−Cl]^+^, calcd 749.3952) *m*/*z*. **Anal**. Calcd for C_36_H_62_ClIrP_2_ (784.51 g mol^−1^): C, 55.12; H, 7.97; N, 0.00. Found: C, 55.26; H, 8.05; N, 0.00.

### Synthesis of *trans*‐[M(2,2′‐biphenyl)(PR_3_)_2_][BAr^F^_4_] (M=Rh, 1; Ir, 2)

*General procedure*: Suspensions of **3**/**4** (1.0 eqv., 10 mm) and Na[BAr^F^_4_]/Li[Al{OC(CF_3_)_3_}_4_] (1.1 eqv.) in CH_2_Cl_2_ (ca. 5 mL) were stirred at RT for 18 hours, diluted with small quantity of pentane and filtered. Crystalline products were obtained upon layering the filtrate with pentane. The supernatant was decanted away and the crystalline materials washed with pentane and dried in vacuo.

**1 a⋅CH_2_Cl_2_**: Prepared from **3 a** (20.0 mg, 24.5 μmol) and Na[BAr^F^_4_] (23.9 mg, 27.0 μmol). Yield: 33.9 mg (80 %, orange crystals).

^**1**^ **H NMR** (500 MHz, CD_2_Cl_2_): *δ=*7.71–7.76 (m, 8 H, Ar^F^), 7.56 (br, 4 H, Ar^F^), 7.50 (t, ^3^ *J*_HH_=7.5 Hz, 6 H, Ph), 7.32 (t, ^3^ *J*_HH_=7.7 Hz, 12 H, Ph), 6.99–7.05 (m, 14 H, Ph+biph), 6.87 (t, ^3^ *J*_HH_=7.3 Hz, 2 H, biph), 6.76 (td, ^3^ *J*_HH_=7.7 Hz, ^3^ *J*_HH_=1.6 Hz, 2 H, biph), 6.65 (dd, ^3^ *J*_HH_=7.5 Hz, ^3^ *J*_RhH_=1.6 Hz, 2 H, biph). ^**13**^ **C{^1^H} NMR** (126 MHz, CD_2_Cl_2_): *δ=*162.3 (q, ^1^ *J*_CB_=50 Hz, Ar^F^), 154.8 (dt, ^1^ *J*_RhC_=39 Hz, ^2^ *J*_PC_=10 Hz, biph), 150.0 (s, biph), 135.4 (s, Ar^F^), 134.0 (t, *J*_PC_=6 Hz, Ph), 132.4 (s, Ph), 131.7 (s, biph), 129.7 (t, *J*_PC_=5 Hz, Ph), 129.4 (qq, ^2^ *J*_FC_=32 Hz, ^3^ *J*_BC_=3 Hz, Ar^F^), 126.9 (t, *J*_PC_=24 Hz, Ph), 126.5 (s, biph), 125.3 (s, biph), 125.2 (q, ^1^ *J*_FC_=272 Hz, Ar^F^), 123.6 (s, biph), 118.0 (sept, ^3^ *J*_FC_=4 Hz, Ar^F^). ^**31**^ **P{^1^H} NMR** (202 MHz, CD_2_Cl_2_): *δ*=19.7 (d, ^1^ *J*_RhP_=118 Hz). **HR ESI‐MS** (positive ion): 779.1507 ([*M*−CH_2_Cl_2_]^+^, calcd 779.1498) *m*/*z*. **Anal**. Calcd for C_81_H_52_BCl_2_F_24_P_2_Rh (1727.83 g mol^−1^): C, 56.31; H, 3.03; N, 0.00. Found: C, 56.42; H, 3.03; N, 0.00.

**1 b**: Prepared from **3 b** (30.0 mg, 35.2 μmol) and Na[BAr^F^_4_] (34.3 mg, 38.8 μmol). Yield: 42.4 mg (71 %, yellow crystals). Additional single crystals for analysis by X‐ray diffraction were grown by recrystallisation of this material from 1,2‐difluorobenzene/pentane.

^**1**^ **H NMR** (500 MHz, CD_2_Cl_2_): *δ*=7.71–7.75 (m, 8 H, Ar^F^), 7.56 (br, 4 H, Ar^F^), 7.48 (dd, ^3^ *J*_HH_=7.5 Hz, ^4^ *J*_HH_=1.6 Hz, 2 H, biph), 7.16 (t, ^3^ *J*_HH_=7.3 Hz, 2 H, biph), 7.11 (d, ^3^ *J*_HH_=8.1 Hz, 2 H, biph), 7.01 (td, ^3^ *J*_HH_=7.7 Hz, ^4^ *J*_HH_=1.6 Hz, 2 H, biph), 1.95–2.05 (m, 6 H, Cy), 1.67–1.77 (m, 18 H, Cy), 1.24–1.38 (m, 24 H, Cy), 1.13–1.24 (m, 18 H, Cy). ^**13**^ **C{^1^H} NMR** (126 MHz, CD_2_Cl_2_): *δ=*162.3 (q, ^1^ *J*_CB_=50 Hz, Ar^F^), 153.8 (dt, ^1^ *J*_RhC_=44 Hz, ^2^ *J*_PC_=8 Hz, biph), 148.8 (d, ^2^ *J*_RhC_=4 Hz, biph), 135.4 (s, Ar^F^), 129.8 (s, biph), 129.4 (qq, ^2^ *J*_FC_=32 Hz, ^3^ *J*_BC_=3 Hz, Ar^F^), 128.0 (s, biph), 125.5 (s, biph), 125.2 (q, ^1^ *J*_FC_=272 Hz, Ar^F^), 122.1 (d, ^2^ *J*_RhC_=2 Hz, biph), 118.0 (sept, ^3^ *J*_FC_=4 Hz, Ar^F^), 35.1 (t, *J*_PC_=10 Hz, Cy), 30.4 (s, Cy), 27.9 (t, *J*_PC_=5 Hz, Cy), 26.3 (s, Cy). ^**31**^ **P{^1^H} NMR** (202 MHz, CD_2_Cl_2_): *δ*=13.4 (d, ^1^ *J*_RhP_=109 Hz). **HR ESI‐MS** (positive ion): 815.4325 ([*M*]^+^, calcd 815.4315) *m*/*z*. **Anal**. Calcd for C_80_H_86_BF_24_P_2_Rh (1679.19 g mol^−1^): C, 57.22; H, 5.16; N, 0.00. Found: C, 57.38; H, 5.26; N, 0.00.

**1 c**: Prepared from **3 c** (20.0 mg, 32.7 μmol) and Na[BAr^F^_4_] (31.9 mg, 36.0 μmol). Yield: 27.1 mg (58 %, orange crystals). Spectroscopic data is fully consistent with previously reported values.^13^

^**1**^ **H NMR** (500 MHz, CD_2_Cl_2_): *δ*=7.70–7.75 (m, 8 H, Ar^F^), 7.56 (br, 4 H, Ar^F^), 7.46 (dd, ^3^ *J*_HH_=7.6 Hz, ^4^ *J*_HH_=1.6 Hz, 2 H, biph), 7.24 (d, ^3^ *J*_HH_=8.1 Hz, 2 H, biph), 7.15 (t, ^3^ *J*_HH_=7.4 Hz, 2 H, biph), 6.98 (td, ^3^ *J*_HH_=7.8 Hz, ^4^ *J*_HH_=1.6, 2 H, biph), 2.24–2.36 (m, 6 H, CH(CH_3_)_2_), 1.02 (app. q, *J=*7 Hz, 36 H, CH_3_). ^**13**^ **C{^1^H} NMR** (126 MHz, CD_2_Cl_2_): *δ=*162.3 (q, ^1^ *J*_CB_=50 Hz, Ar^F^), 152.1 (dt, ^1^ *J*_RhC_=44 Hz, ^2^ *J*_PC_=8 Hz, biph), 148.6 (d, ^2^ *J*_RhC_=5 Hz, biph), 135.4 (s, Ar^F^), 129.6 (s, biph), 129.4 (qq, ^2^ *J*_FC_=32 Hz, ^3^ *J*_CB_=3 Hz, Ar^F^), 128.0 (s, biph), 125.8 (s, biph), 125.2 (q, ^1^ *J*_FC_=272 Hz, Ar^F^), 122.5 (s, biph), 118.0 (sept, ^3^ *J*_FC_=4 Hz, Ar^F^), 24.3 (t, *J*_PC_=11 Hz, CH(CH_3_)_2_), 19.7 (s, CH_3_). ^**31**^ **P{^1^H} NMR** (202 MHz, CD_2_Cl_2_): *δ=*25.7 (d, ^1^ *J*_RhP_=112). **LR ESI‐MS** (positive ion): 575.2 ([*M*]^+^, calcd 575.2) *m*/*z*.

**1 d**: Prepared from **3 d** (25.1 mg, 36.1 μmol) and Na[BAr^F^_4_] (35.2 mg, 39.7 μmol). Yield: 39.3 mg (71 %, orange crystals).

^**1**^ **H NMR** (500 MHz, CD_2_Cl_2_): *δ=*7.71–7.75 (m, 8 H, Ar^F^), 7.56 (br, 4 H, Ar^F^), 7.52 (dd, ^3^ *J*_HH_=7.5 Hz, ^4^ *J*_HH_=1.6 Hz, 2 H, biph), 7.17 (t, ^3^ *J*_HH_=7.4 Hz, 2 H, biph), 7.08 (d, ^3^ *J*_HH_=8.0 Hz, 2 H, biph), 6.98 (td, ^3^ *J*_HH_=7.7 Hz, ^4^ *J*_HH_=1.6 Hz, 2 H, biph), 1.57–1.71 (m, 6 H, CH_2_CH), 1.43 (app. dt, *J=*6 Hz, *J=*3 Hz, 12 H, CH_2_), 0.75 (d, ^3^ *J*_HH_=6.6 Hz, 36 H, CH_3_). ^**13**^ **C{^1^H} NMR** (126 MHz, CD_2_Cl_2_): *δ=*162.3 (q, ^1^ *J*_CB_=50 Hz, Ar^F^), 156.6 (dt, ^1^ *J*_RhC_=43 Hz, ^2^ *J*_PC_=9 Hz, biph), 148.7 (d, ^2^ *J*_RhC_=4 Hz, biph), 135.4 (Ar^F^), 130.2 (app. t, *J=*2 Hz, biph), 129.4 (qq, ^2^ *J*_FC_=31 Hz, ^3^ *J*_CB_=3 Hz, Ar^F^), 128.0 (s, biph), 125.6 (biph), 125.2 (q, ^1^ *J*_FC_=272 Hz, Ar^F^), 123.1 (s, biph), 118.0 (sept, ^3^ *J*_FC_=4 Hz, Ar^F^), 33.2 (t, *J*_PC_=12 Hz, CH_2_), 25.9 (t, *J*_PC_=3 Hz, CH_2_ CH), 25.6 (s, CH_3_). ^**31**^ **P{^1^H} NMR** (202 MHz, CD_2_Cl_2_): *δ=*18.5 (d, ^1^ *J*_RhP_=110 Hz). **HR ESI‐MS** (positive ion): 659.3383 ([*M*]^+^, calcd 659.3376) *m*/*z*. **Anal**. Calcd for C_68_H_74_BF_24_P_2_Rh (1522.96 g mol^−1^): C, 53.63; H, 4.90; N, 0.00. Found: C, 53.76; H, 4.81; N, 0.00.

1 d*: Prepared from **3 d** (5.0 mg, 7.2 μmol) and Li[Al{OC(CF_3_)_3_}_4_] (7.7 mg, 7.9 μmol). Yield: 4.4 mg (38 %, orange crystals).

^**1**^ **H NMR** (300 MHz, CD_2_Cl_2_): *δ=*7.52 (d, ^3^ *J*_HH_=7.6 Hz, 2 H, biph), 7.18 (t, ^3^ *J*_HH_=7.6 Hz, 2 H, biph), 7.08 (d, ^3^ *J*_HH_=8.1 Hz, 2 H, biph), 6.99 (t, ^3^ *J*_HH_=7.6 Hz, 2 H, biph), 1.56–1.74 (m, 6 H, CH_2_CH), 1.44 (br, fwhm=12 Hz, 12 H, CH_2_), 0.77 (d, ^3^ *J*_HH_=6.3 Hz, 36 H, CH_3_). ^**31**^ **P{^1^H} NMR** (121 MHz, CD_2_Cl_2_): *δ=*18.5 (d, ^1^ *J*_RhP_=110 Hz). **LR ESI‐MS** (positive ion): 659.3 ([M]^+^, calcd 659.3) *m*/*z*.

**2 a⋅CH_2_Cl_2_**: Prepared from **4 a** (20.0 mg, 22.1 μmol) and Na[BAr^F^_4_] (21.6 mg, 24.3 μmol) according to a modification of the general procedure: the suspension was heating at 50 °C for 72 hours. Yield: 12.7 mg (32 %, burgundy crystals).

^**1**^ **H NMR** (500 MHz, CD_2_Cl_2_): *δ=*7.71–7.76 (m, 8 H, Ar^F^), 7.56 (br, 4 H, Ar^F^), 7.50 (t, ^3^ *J*_HH_=7.5 Hz, 6 H, Ph), 7.33 (t, ^3^ *J*_HH_=7.7 Hz, 12 H, Ph), 7.04 (app q, *J=*6 Hz, 12 H, Ph), 6.85 (d, ^3^ *J*_HH_=7.9 Hz, 2 H, biph), 6.77 (t, ^3^ *J*_HH_=7.4 Hz, 2 H, biph), 6.61 (td, ^3^ *J*_HH_=7.7 Hz, ^4^ *J*_HH_=1.6 Hz, 2 H, biph), 6.57 (dd, ^3^ *J*_HH_=7.6 Hz, ^4^ *J*_HH_=1.6 Hz, 2 H, biph). ^1**3**^ **C{^1^H} NMR** (126 MHz, CD_2_Cl_2_): *δ=*162.3 (q, ^1^ *J*_CB_=50 Hz, Ar^F^), 150.8 (s, biph), 135.4 (s, Ar^F^), 134.1 (t, *J*_PC_=6 Hz, Ph), 132.4 (s, Ph), 130.5 (s, biph), 129.7 (t, *J*_PC_=5 Hz, Ph), 129.4 (q, ^2^ *J*_FC_=31 Hz, Ar^F^), 127.0 (t, ^2^ *J*_PC_=7 Hz, biph), 126.5 (t, *J*_PC_=28 Hz, Ph), 126.1 (s, biph), 125.2 (s, biph), 125.2 (q, ^1^ *J*_FC_=272 Hz, Ar^F^), 122.6 (s, biph), 118.0 (sept, Ar^F^). ^**31**^ **P{^1^H} NMR** (202 MHz, CD_2_Cl_2_): *δ=*11.6 (s). **HR ESI‐MS** (positive ion): 869.2088 ([*M*−CH_2_Cl_2_]^+^, calcd 869.2076) *m*/*z*. **Anal**. Calcd for C_81_H_52_BCl_2_F_24_P_2_Ir (1817.14 g mol^−1^): C, 53.54; H, 2.88; N, 0.00. Found: C, 53.67; H, 3.01; N, 0.00.

**2 b**: Prepared from **4 b** (30.0 mg, 30.3 μmol) and Na[BAr^F^_4_] (28.2 mg, 38.8 μmol). Yield: 42.0 mg (75 %, orange crystals). Additional single crystals for analysis by X‐ray diffraction were grown by recrystallisation of this material from 1,2‐C_6_H_4_F_2_/pentane.

^**1**^ **H NMR** (500 MHz, CD_2_Cl_2_): *δ=*7.70–7.75 (m, 8 H, Ar^F^), 7.56 (br, 4 H, Ar^F^), 7.43 (dd, ^3^ *J*_HH_=7.6 Hz, ^4^ *J*_HH_=1.6 Hz, 2 H, biph), 7.07 (t, ^3^ *J*_HH_=7.4 Hz, 2 H, biph), 6.92 (d, ^3^ *J*_HH_=7.9 Hz, 2 H, biph), 6.83 (td, ^3^ *J*_HH_=7.6 Hz, ^4^ *J*_HH_=1.6 Hz, 2 H, biph), 2.06–2.16 (m, 6 H, Cy), 1.68–1.81 (m, 18 H, Cy), 1.34–1.46 (m, 12 H, Cy), 1.10–1.29 (m, 30 H, Cy). ^**13**^ **C{^1^H} NMR** (126 MHz, CD_2_Cl_2_): *δ=*162.3 (q, ^1^ *J*_CB_=50 Hz, Ar^F^), 149.5 (s, biph), 135.4 (s, Ar^F^), 129.4 (qq, ^2^ *J*_FC_=31 Hz, ^3^ *J*_CB_=3 Hz, Ar^F^), 129.0 (s, biph), 127.5 (s, biph), 125.8 (t, ^2^ *J*_PC_=6 Hz, biph), 125.2 (q, ^1^ *J*_FC_=272 Hz, Ar^F^), 125.1 (s, biph), 121.6 (s, biph), 118.0 (s, Ar^F^), 36.3 (t, ^1^ *J*_PC_=12 Hz, Cy), 30.4 (s, Cy), 27.8 (t, *J*_PC_=5 Hz, Cy), 26.3 (s, Cy). ^**31**^ **P{^1^H} NMR** (202 MHz, CD_2_Cl_2_): *δ=*3.0 (s). **HR ESI‐MS** (positive ion): 905.4912 ([*M*]^+^, calcd 905.4893) *m*/*z*. **Anal**. Calcd for C_80_H_86_BF_24_IrP_2_ (1768.50 g mol^−1^): C, 54.33; H, 4.90; N, 0.00. Found: C, 54.34; H, 5.01; N, 0.00.

**2 c**: Prepared from **4 c** (30.0 mg, 42.8 μmol) and Na[BAr^F^_4_] (41.8 mg, 47.2 μmol). Yield: 27.8 mg (64 %, red crystals).

^**1**^ **H NMR** (600 MHz, CD_2_Cl_2_): *δ=*7.71–7.75 (m, 8 H, Ar^F^), 7.56 (br, 4 H, Ar^F^), 7.41 (dd, ^3^ *J*_HH_=7.6 Hz, ^4^ *J*_HH_=1.6 Hz, 2 H, biph), 7.05 (t, ^3^ *J*_HH_=7.4 Hz, 2 H, biph), 6.99 (d, ^3^ *J*_HH_=8.0 Hz, 2 H, biph), 6.79 (dt, ^3^ *J*_HH_=7.7 Hz, ^4^ *J*_HH_=1.6 Hz, 2 H, biph), 2.41–2.51 (m, 6 H, CH(CH_3_)_2_), 1.00 (app. q, *J=*7 Hz, 36 H, CH_3_). ^**13**^ **C{^1^H} NMR** (151 MHz, CD_2_Cl_2_): *δ=*162.3 (q, ^1^ *J*_CB_=50 Hz, Ar^F^), 149.3 (s, biph), 135.4 (s, Ar^F^), 129.4 (qq, ^2^ *J*_FC_=31 Hz, ^3^ *J*_CB_=3 Hz, Ar^F^), 128.3 (s, biph), 127.3 (s, biph), 125.6 (s, biph), 125.2 (q, ^1^ *J*_FC_=272 Hz, Ar^F^), 123.4 (t, ^2^ *J*_PC_=6 Hz, biph), 122.0 (s, biph), 118.0 (s, Ar^F^), 25.3 (t, *J*_PC_=13 Hz, CH(CH_3_)_2_), 19.7 (s, CH_3_). ^**31**^ **P{^1^H} NMR** (202 MHz, CD_2_Cl_2_): *δ=*17.8 (s). **HR ESI‐MS** (positive ion): 665.3021 ([*M*]^+^, calcd 665.3013) *m*/*z*. **Anal**. Calcd for C_62_H_62_BF_24_IrP_2_ (1528.11 g mol^−1^): C, 48.73; H, 4.09; N, 0.00. Found: C, 48.81; H, 4.09; N, 0.00.

**2 d**: Prepared from **4 d** (22.7 mg, 28.9 μmol) and Na[BAr^F^_4_] (28.2 mg, 31.8 μmol). Yield: 27.0 mg (59 %, yellow crystals).

^**1**^ **H NMR** (500 MHz, CD_2_Cl_2_): *δ=*7.70–7.75 (m, 8 H, Ar^F^), 7.56 (br, 4 H, Ar^F^), 7.46 (dd, ^3^ *J*_HH_=7.7 Hz, ^4^ *J*_HH_=1.6 Hz, 2 H, biph), 7.10 (t, ^3^ *J*_HH_=7.4 Hz, 2 H, biph), 6.97 (d, ^3^ *J*_HH_=7.8 Hz, 2 H, biph), 6.84 (td, ^3^ *J*_HH_=7.6 Hz, ^4^ *J*_HH_=1.6 Hz, 2 H, biph), 1.59–1.72 (m, 6 H, CH_2_CH), 1.54 (app. dt, *J=*7 Hz, *J=*3 Hz, 12 H, CH_2_), 0.71 (d, ^3^ *J*_HH_=6.5 Hz, 36 H, CH_3_). ^**13**^ **C{^1^H} NMR** (126 MHz, CD_2_Cl_2_): *δ=*162.3 (q, ^1^ *J*_CB_=50 Hz, Ar^F^), 149.4 (s, biph), 135.4 (s, Ar^F^), 130.3 (t, ^2^ *J*_PC_=7 Hz, biph), 129.6 (s, biph), 129.4 (qq, ^2^ *J*_FC_=32 Hz, ^3^ *J*_CB_=3 Hz, Ar^F^), 127.7 (s, biph), 125.3 (s, biph), 125.2 (q, ^1^ *J*_FC_=273 Hz, Ar^F^), 122.4 (s, biph), 118.0 (sept, ^3^ *J*_FC_=4 Hz, Ar^F^), 34.0 (t, *J*_PC_=14 Hz, CH_2_), 25.8–26.0 (m, CH_2_ CH + CH_3_). ^**31**^ **P{^1^H} NMR** (202 MHz, CD_2_Cl_2_): *δ=*14.5 (s). **HR ESI‐MS** (positive ion): 749.3952 ([*M*]^+^, calcd 749.3952) *m*/*z*. **Anal**. Calcd for C_68_H_74_BF_24_IrP_2_ (1612.28 g mol^−1^): C, 50.66; H, 4.63; N, 0.00. Found: C, 50.80; H, 4.72; N, 0.00.

2 d*: Prepared from **4 d** (5.0 mg, 7.2 μmol) and Li[Al{OC(CF_3_)_3_}_4_] (7.7 mg, 7.9 μmol). Yield: 4.0 mg (34 %, yellow crystals).

^**1**^ **H NMR** (400 MHz, CD_2_Cl_2_): *δ=*7.46 (dd, ^3^ *J*_HH_=7.6 Hz, ^4^ *J*_HH_=1.6 Hz, 2 H, biph), 7.11 (t, ^3^ *J*_HH_=7.3 Hz, 2 H, biph), 6.98 (d, ^3^ *J*_HH_=7.8 Hz, 2 H, biph), 6.84 (td, ^3^ *J*_HH_=7.6 Hz, ^4^ *J*_HH_=1.6 Hz, 2 H, biph), 1.59–1.74 (m, 6 H, CH_2_CH), 1.54 (app. dt, *J=*7 Hz, *J=*3 Hz, 12 H, CH_2_), 0.72 (d, ^3^ *J*_HH_=6.5 Hz, 36 H, CH_3_). ^**31**^ **P{^1^H} NMR** (162 MHz, CD_2_Cl_2_): *δ=*13.8 (s). **LR ESI‐MS** (positive ion): 749.5 ([*M*]^+^, calcd 749.4) *m*/*z*.

### Crystallography

https://summary.ccdc.cam.ac.uk/structure-summary?doi=10.1002/chem.201705990 1590085–1590103 contain the supplementary crystallographic data for this paper, including full details about the collection, solution and refinement. These data are provided free of charge by http://www.ccdc.cam.ac.uk/.

### Variable temperature NMR spectroscopy

Variable temperature measurements were performed using 9.0 mm of complex in CD_2_Cl_2_ solution (0.5 mL). Data for **1 a** and **2 a** were collected in the presence of powdered 3 Å molecular sieves. Spectra were recorded on a Bruker AV‐500 spectrometer at 298, 273, 250, 225, 200 and 185 K; samples were held for ten minutes at the desired temperature before acquisition.

### Computational methods

All molecular geometries were optimised using Gaussian 09,^37^ at the pbe0/def2‐tzvp level of theory.^32^ NBO analyses were carried out using NBO 6.0, and QTAIM analyses using AIMAll.^34^

### Supporting information

^1^H, ^13^C{^1^H} and ^31^P{^1^H} NMR spectra, and HR ESI‐MS of **1**, **2**, **3** and **4**. Additional discussion centred on crystallographic disorder observed in the solid‐state structures of **1** and **2**. Optimised structures of **1′**, **2′** and isomers **1 c′′** and **2 c′′** in .xyz format. Selected output from NBO and QTAIM analysis of **1′** and **2′**. CCDC 1590085‐1590103.

## Conflict of interest

The authors declare no conflict of interest.

## Supporting information

As a service to our authors and readers, this journal provides supporting information supplied by the authors. Such materials are peer reviewed and may be re‐organized for online delivery, but are not copy‐edited or typeset. Technical support issues arising from supporting information (other than missing files) should be addressed to the authors.

Supplementary
