# Supplementary material for: Solution, Solid‐State, and Computational Analysis of Agostic Interactions in a Coherent Set of Low‐Coordinate Rhodium(III) and Iridium(III) Complexes
Source: Chemistry. 2018 Feb 28;24(19):4927–38. doi: 10.1002/chem.201705990 (PMC5901041; doi:10.1002/chem.201705990)
Supplement: Supplementary file 1 — Supplementary [file CHEM-24-4927-s001.pdf]

CHEMISTRY
A European Journal

Supporting Information

Solution, Solid-State, and Computational Analysis of Agostic Interactions in a Coherent Set of Low-Coordinate Rhodium(III) and Iridium(III) Complexes

Richard C. Knighton,[a] Jack Emerson-King,[a] Jonathan P. Rourke,[a] C. André Ohlin,[b] and Adrian B. Chaplin*[a]

chem_201705990_sm_miscellaneous_information.pdf

Contents

1. Selected NMR spectra 2
1.1. [Rh(2,2’-biphenyl)(PPh_3)$_2$Cl] **3a** 2
1.2. [Rh(2,2’-biphenyl)(PCy_3)$_2$Cl] **3b** 3
1.3. [Rh(2,2’-biphenyl)(P*i*Pr_3)$_2$Cl] **3c** 4
1.4. [Rh(2,2’-biphenyl)(P*i*Bu_3)$_2$Cl] **3d** 5
1.5. [Ir(2,2’-biphenyl)(PPh_3)$_2$Cl] **4a** 6
1.6. [Ir(2,2’-biphenyl)(PCy_3)$_2$Cl] **4b** 7
1.7. [Ir(2,2’-biphenyl)(P*i*Pr_3)$_2$Cl] **4c** 8
1.8. [Ir(2,2’-biphenyl)(P*i*Bu_3)$_2$Cl] **4d** 9
1.9. [Rh(2,2’-biphenyl)(PPh_3)$_2$][BAr^F_4] **1a** 10
1.10. [Rh(2,2’-biphenyl)(PCy_3)$_2$][BAr^F_4] **1b** 12
1.11. [Rh(2,2’-biphenyl)(P*i*Pr_3)$_2$][BAr^F_4] **1c** 13
1.12. [Rh(2,2’-biphenyl)(P*i*Bu_3)$_2$][BAr^F_4] **1d** 14
1.13. [Rh(2,2’-biphenyl)(P*i*Bu_3)$_2$][Al{OC(CF_3)}$_4$] **1d*** 15
1.14. [Ir(2,2’-biphenyl)(PPh_3)$_2$][BAr^F_4] **2a** 16
1.15. [Ir(2,2’-biphenyl)(PCy_3)$_2$][BAr^F_4] **2b** 17
1.16. [Ir(2,2’-biphenyl)(P*i*Pr_3)$_2$][BAr^F_4] **2c** 18
1.17. [Ir(2,2’-biphenyl)(P*i*Bu_3)$_2$][BAr^F_4] **2d** 19
1.18. [Ir(2,2’-biphenyl)(P*i*Bu_3)$_2$][Al{OC(CF_3)}$_4$] **2d*** 20
2. Variable temperature ^1H and $^{31}P\{^1H\}$ NMR spectra 21
3. Selected HR ESI-MS 30
4. Crystallography 37
4.1. Disorder in **1b** and **2b** 38
4.2. Disorder in **1c** and **2c** 39
4.3. Disorder in **1d** and **2d**, and differences between the unit cells of **1d**/**2d** and **1d***/**2d*** 40
4.4. More extensive metrics associated with the solid-state structures of **1** and **2**. 43
4.5. Structures of **1a·OH$_2$** and **2a·OH$_2$** 45
5. Computational analysis 46
5.1. Selected NBO orbital overlaps associated with perturbation analysis 46
5.2. QTAIM molecular graphs 54

1. Selected NMR spectra

1.1. [Rh(2,2'-biphenyl)(PPh$_3$)$_2$Cl] ***3a***

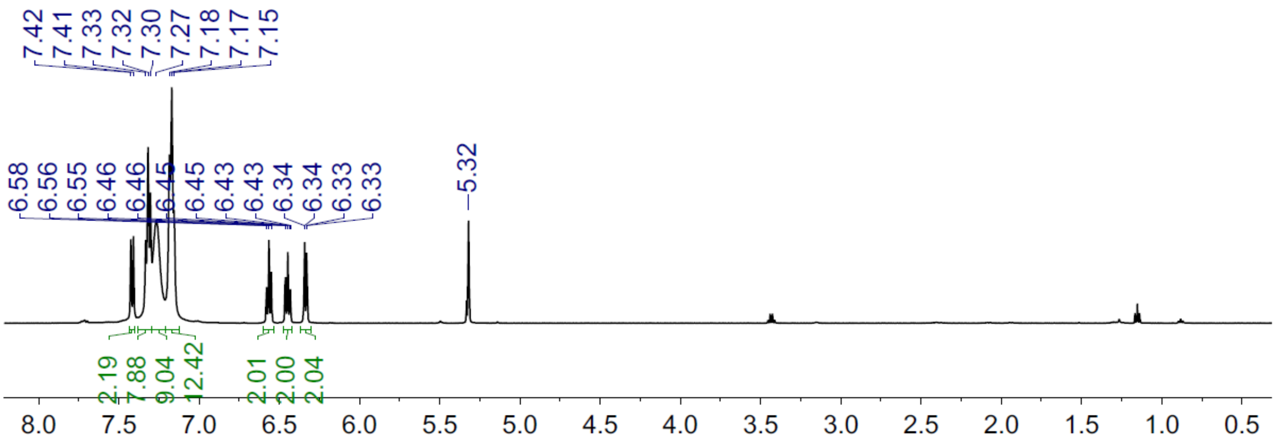

Figure S1: ^{1}H NMR spectrum of **3a** (500 MHz, CD_2Cl_2)

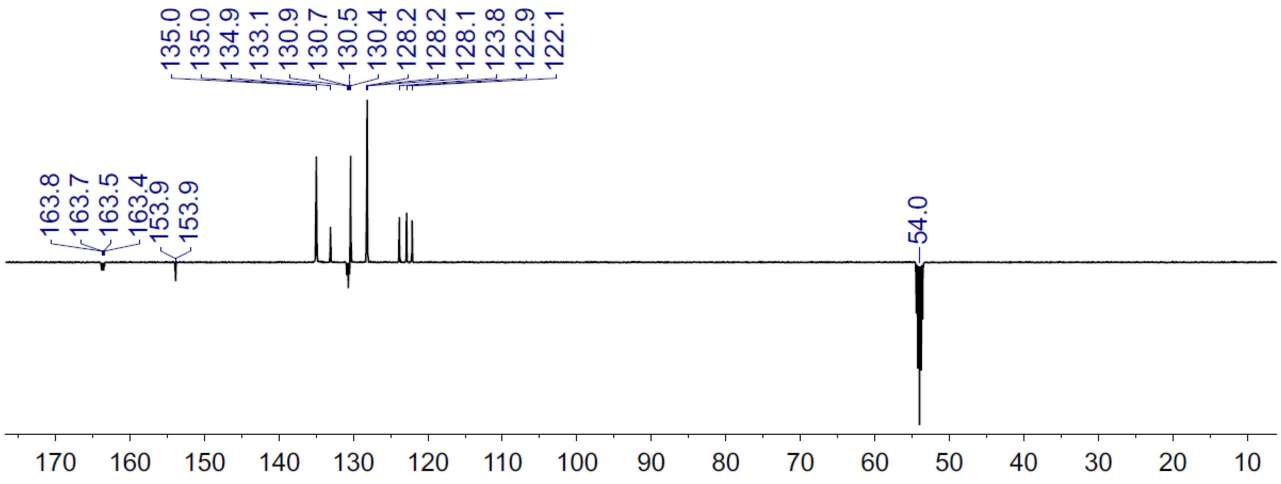

Figure S2: $^{13}C\{^{1}H\}$ APT NMR spectrum of **3a** (126 MHz, CD_2Cl_2)

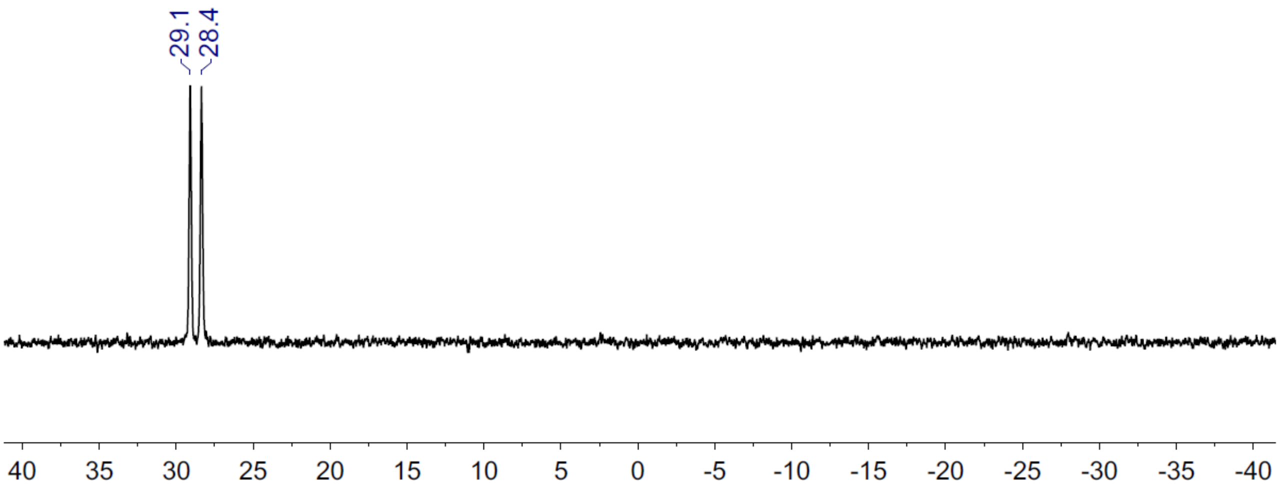

Figure S3: $^{31}P\{^{1}H\}$ NMR spectrum of **3a** (162 MHz, CD_2Cl_2)

1.2. [Rh(2,2'-biphenyl)(PCy$_3$)$_2$Cl] ***3b***

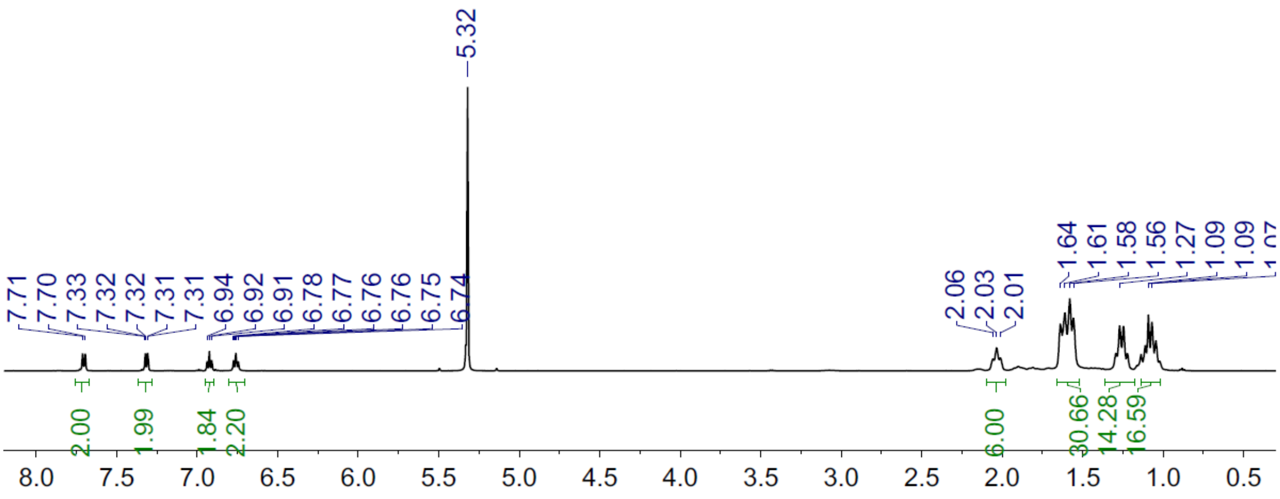

Figure S4: ^{1}H NMR spectrum of **3b** (500 MHz, CD_2Cl_2)

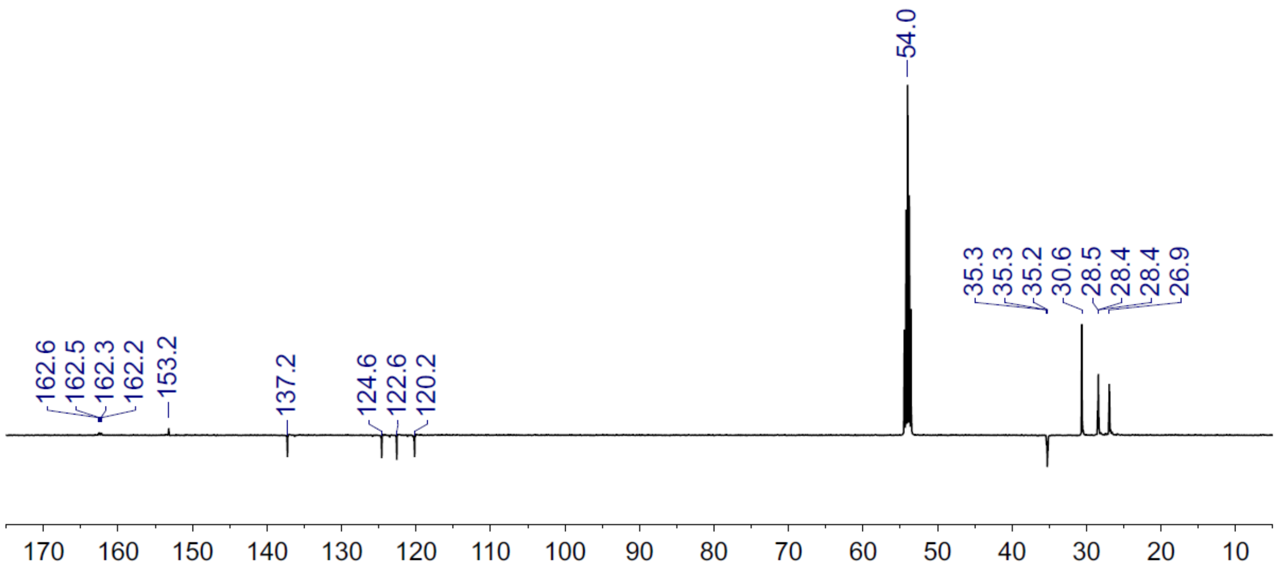

Figure S5: ^{13}C{^{1}H} APT NMR spectrum of **3b** (126 MHz, CD_2Cl_2)

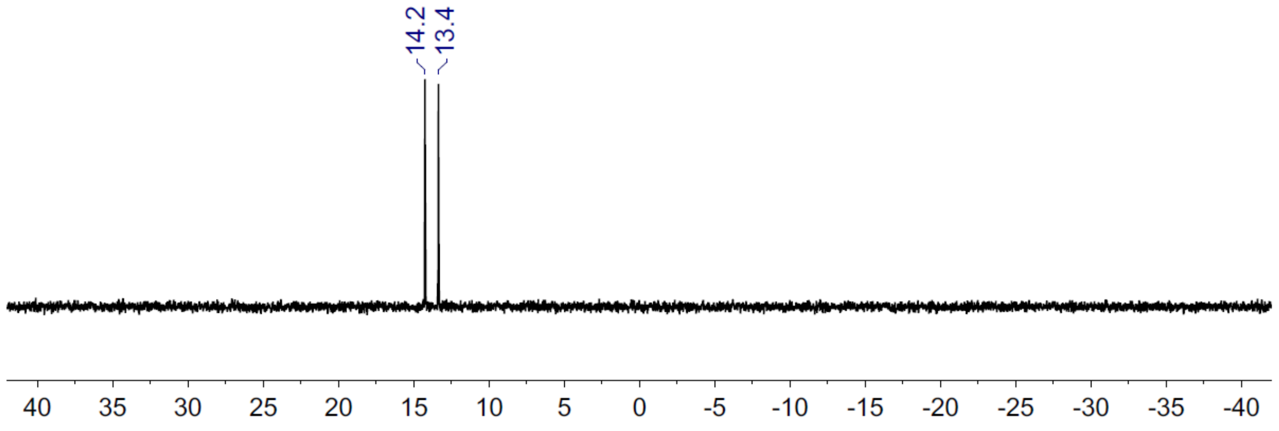

Figure S6: ^{31}P{^{1}H} NMR spectrum of **3b** (121 MHz, CD_2Cl_2)

1.3. *[Rh(2,2'-biphenyl)(PiPr$_3$)$_2$Cl]* ***3c***

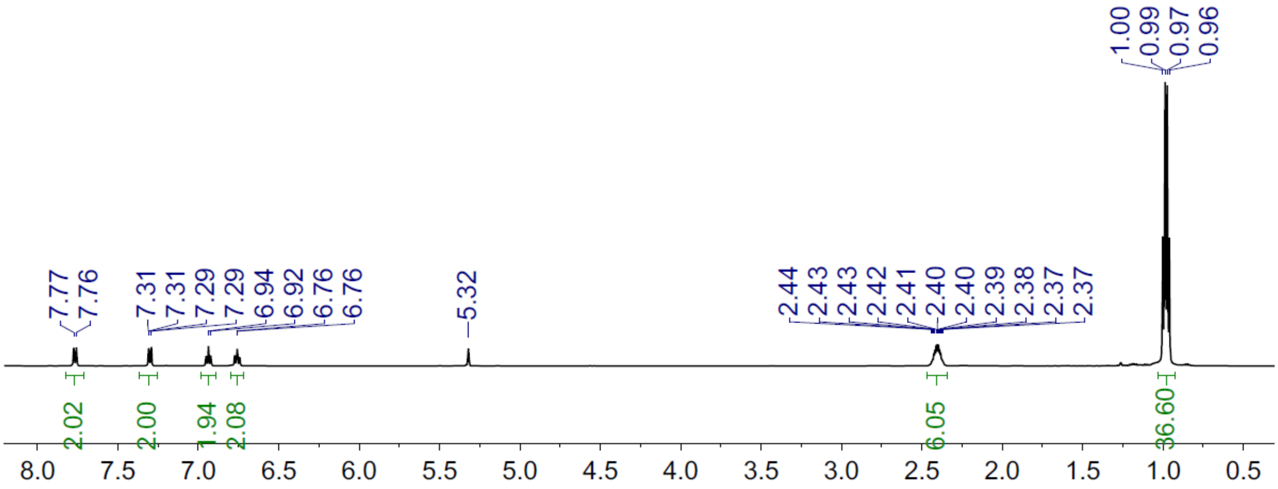

Figure S7: ^{1}H NMR spectrum of **3c** (500 MHz, CD_2Cl_2)

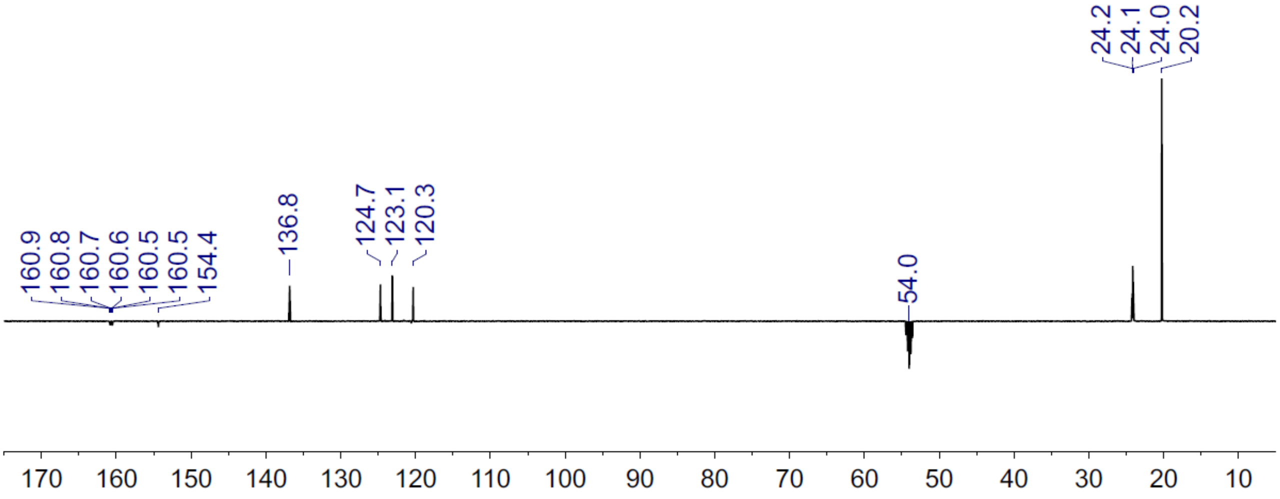

Figure S8: $^{13}C\{^{1}H\}$ APT NMR spectrum of **3c** (126 MHz, CD_2Cl_2)

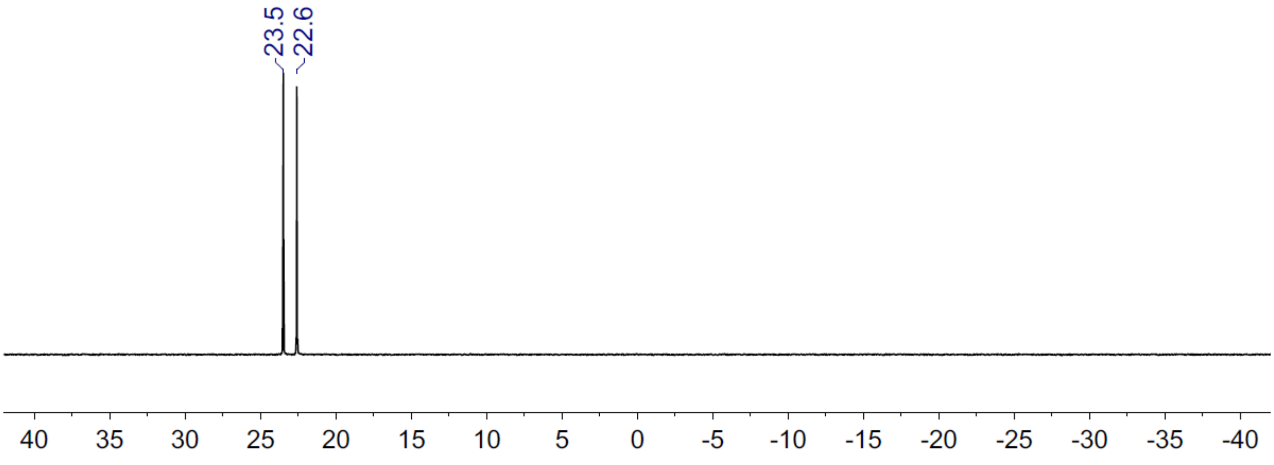

Figure S9: $^{31}P\{^{1}H\}$ NMR spectrum of **3c** (121 MHz, CD_2Cl_2)

1.4. [Rh(2,2′-biphenyl)(PiBu$_3$)$_2$Cl] ***3d***

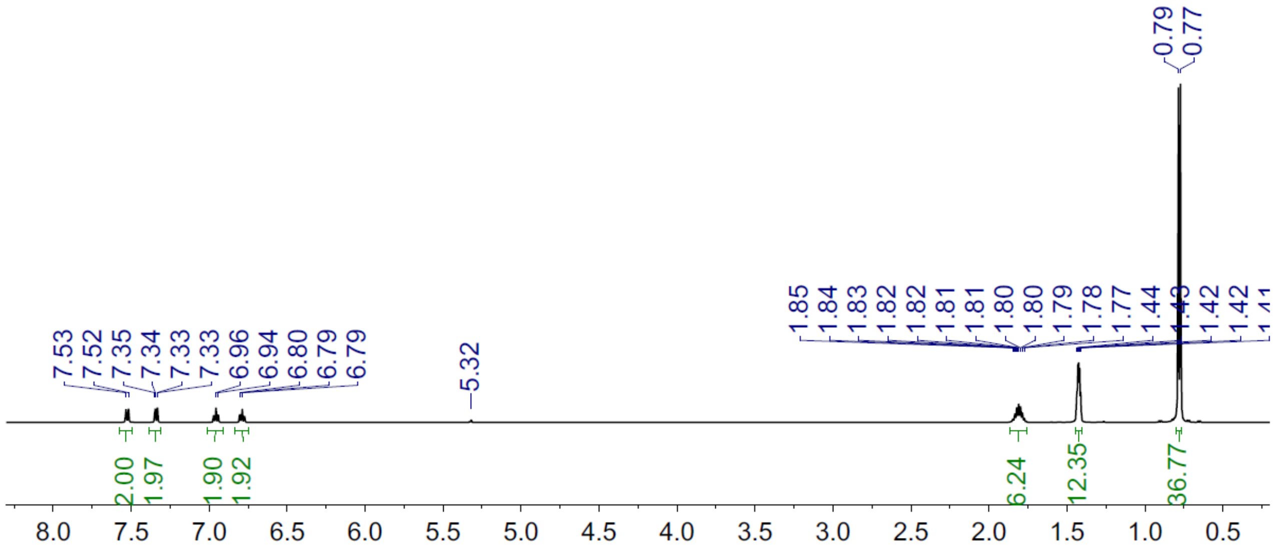

Figure S10: ^{1}H NMR spectrum of **3d** (500 MHz, CD_2Cl_2)

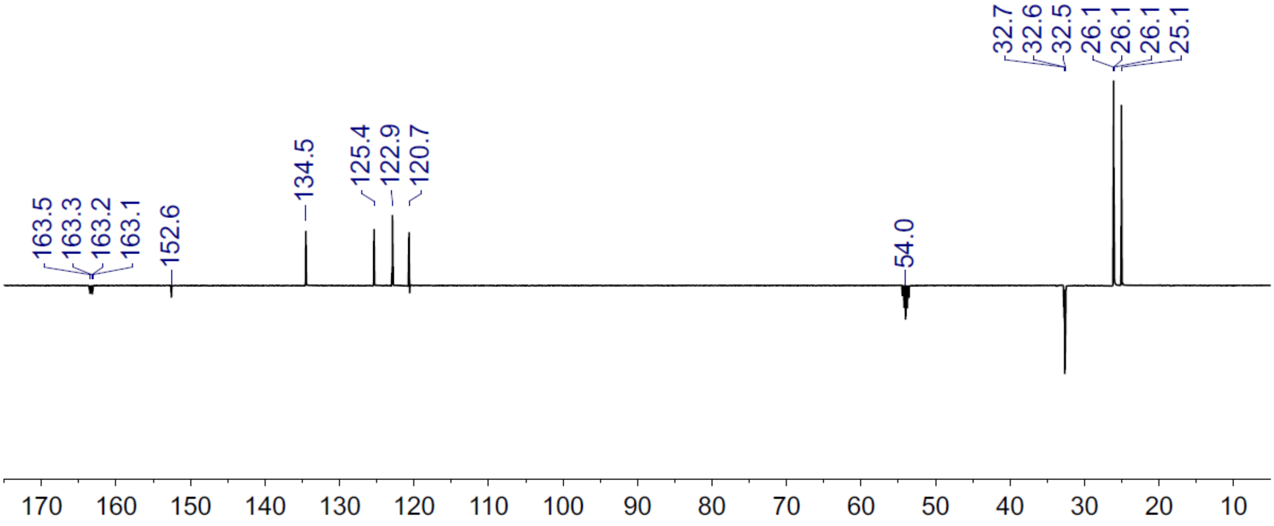

Figure S11: $^{13}C\{^{1}H\}$ APT NMR spectrum of **3d** (126 MHz, CD_2Cl_2)

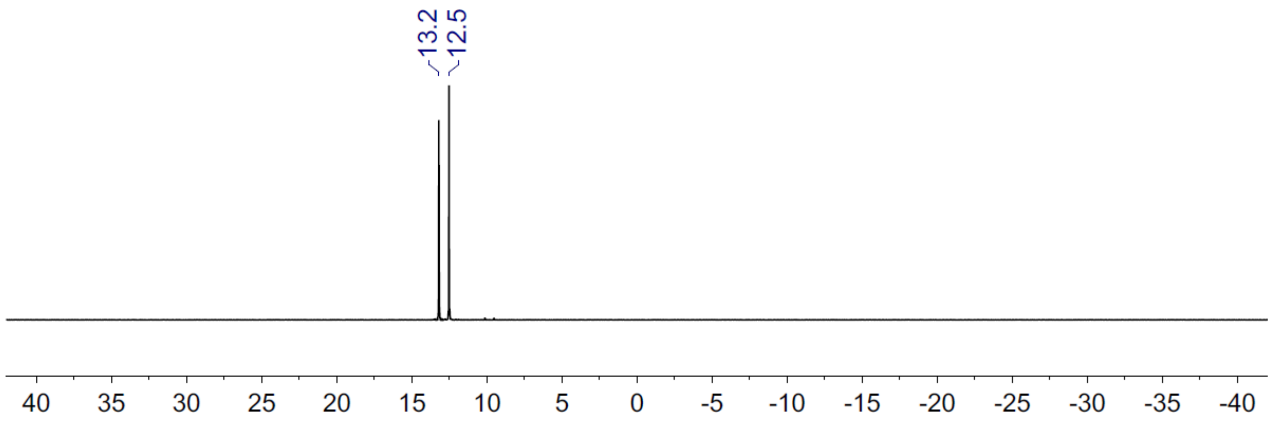

Figure S12: $^{31}P\{^{1}H\}$ NMR spectrum of **3d** (162 MHz, CD_2Cl_2)

1.5. [Ir(2,2'-biphenyl)(PPh_3)$_2$Cl] ***4a***

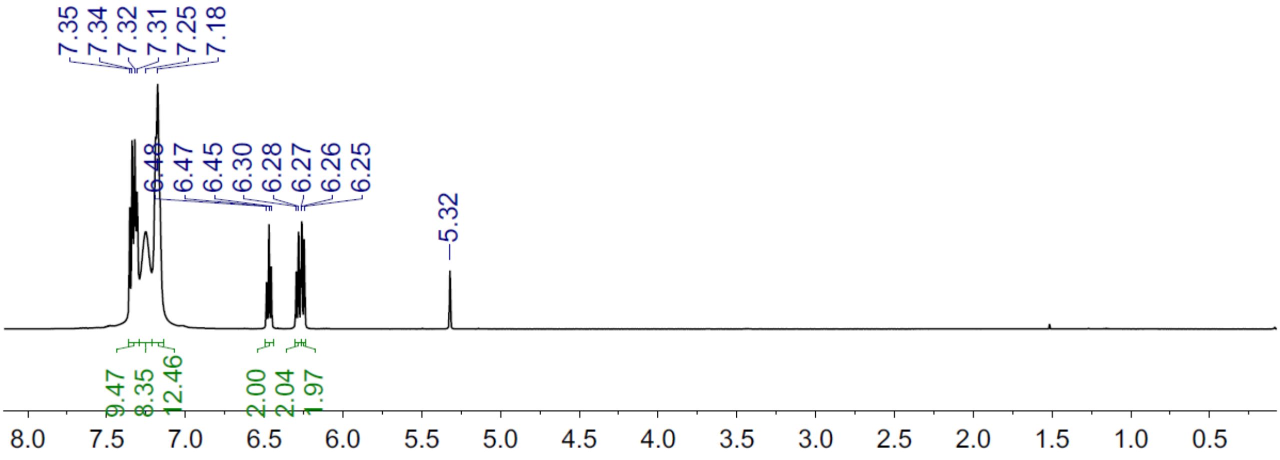

Figure S13: ^{1}H NMR spectrum of **4a** (500 MHz, CD_2Cl_2)

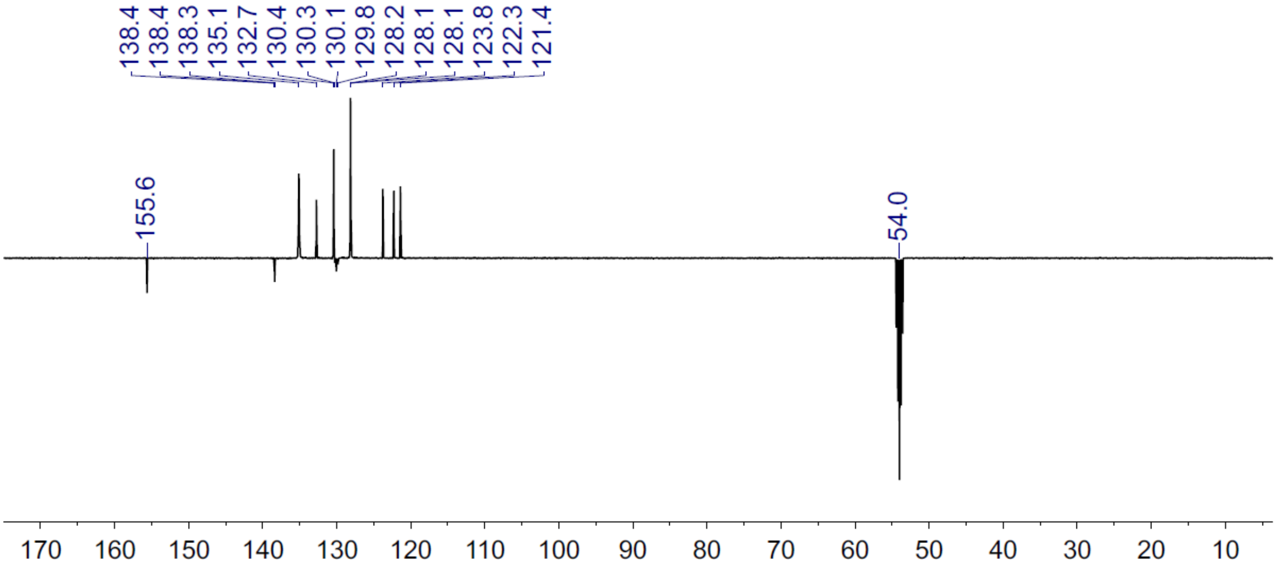

Figure S14: ^{13}C{^{1}H} APT NMR spectrum of **4a** (126 MHz, CD_2Cl_2)

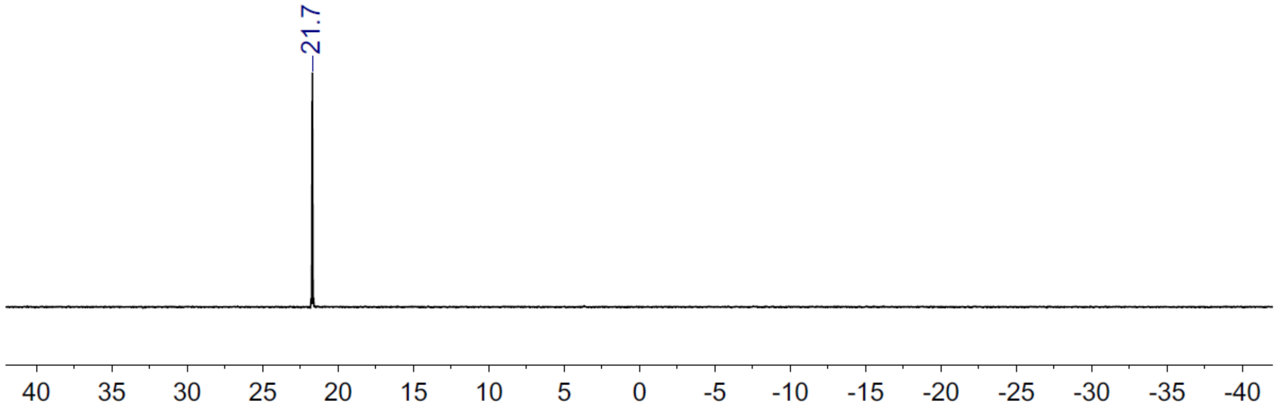

Figure S15: ^{31}P{^{1}H} NMR spectrum of **4a** (162 MHz, CD_2Cl_2)

1.6. [Ir(2,2'-biphenyl)(PCy_3)$_2$Cl] ***4b***

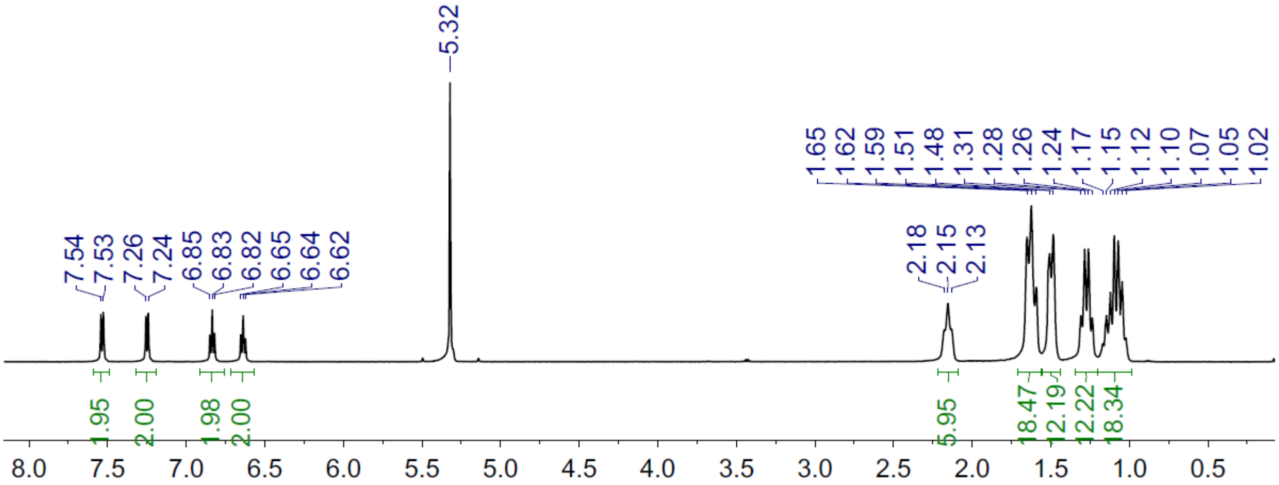

Figure S16: ^{1}H NMR spectrum of **4b** (500 MHz, CD_2Cl_2)

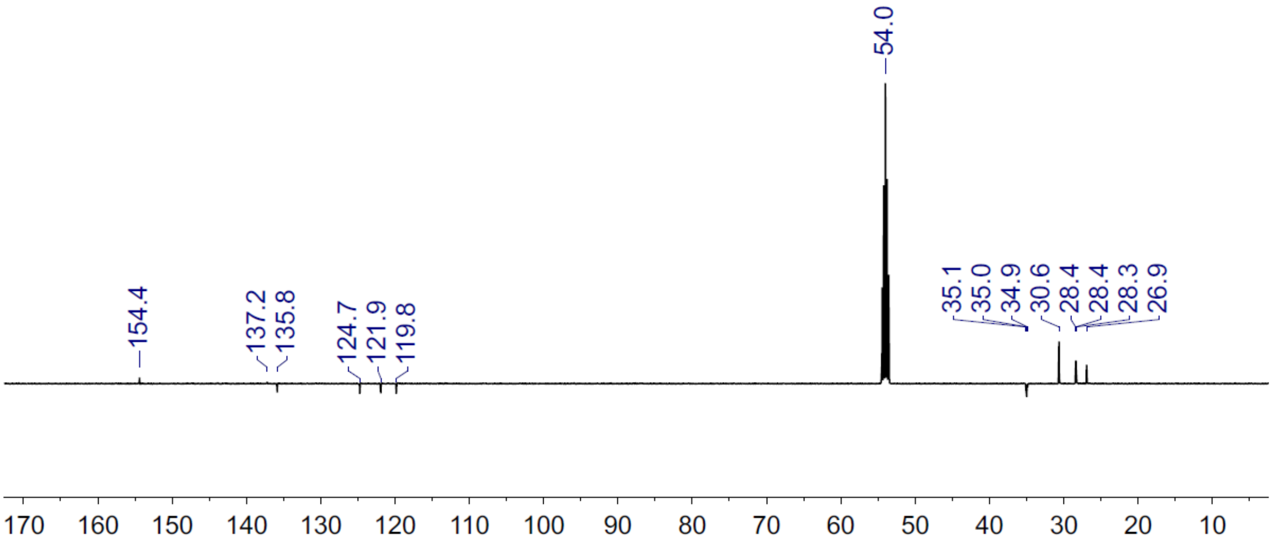

Figure S17: ^{13}C{^{1}H} APT NMR spectrum of **4b** (126 MHz, CD_2Cl_2)

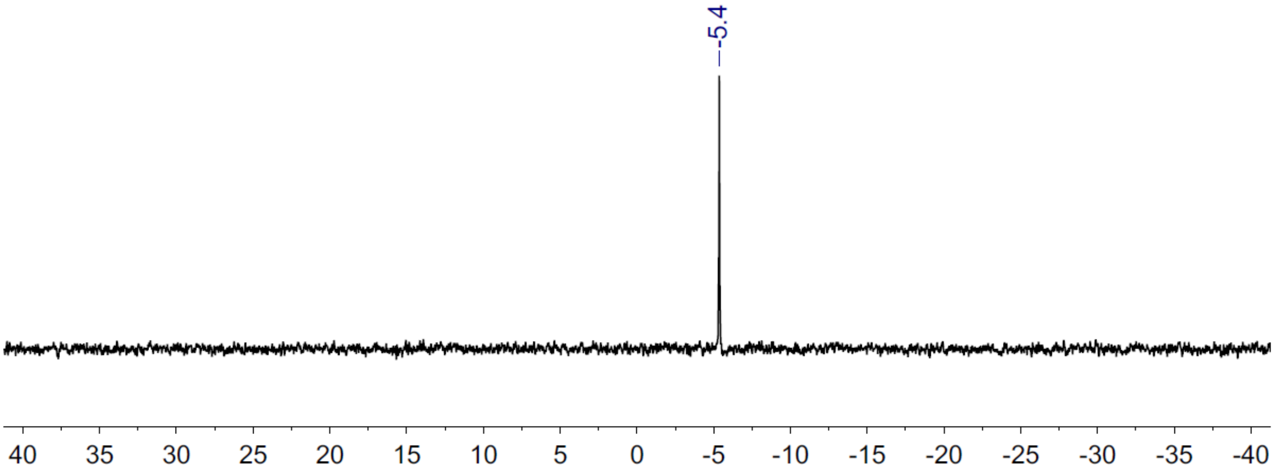

Figure S18: ^{31}P{^{1}H} NMR spectrum of **4b** (162 MHz, CD_2Cl_2)

1.7. [Ir(2,2'-biphenyl)(PiPr$_3$)$_2$Cl] ***4c***

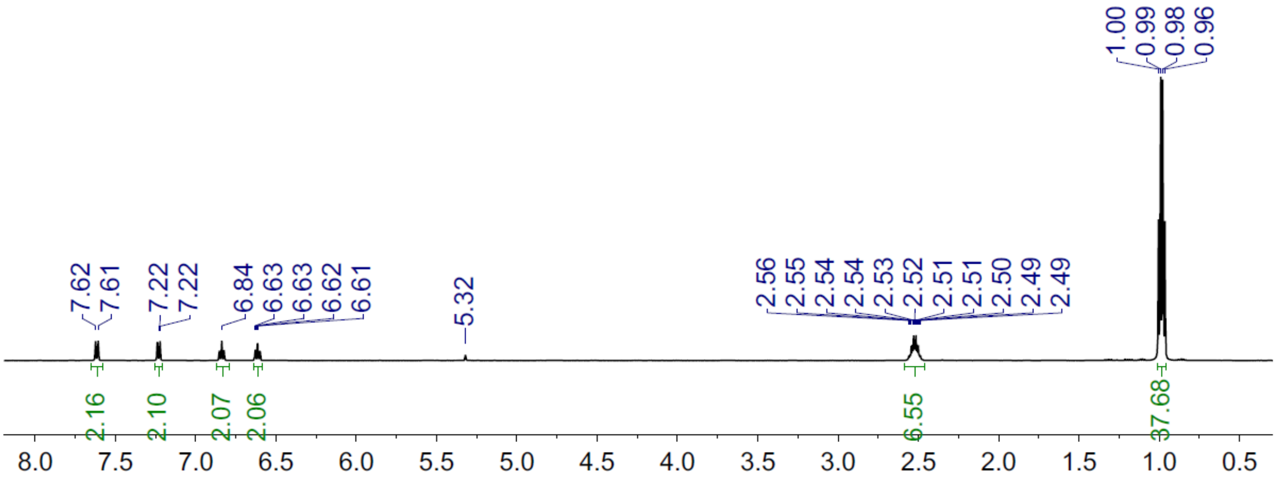

Figure S19: ^{1}H NMR spectrum of **4c** (500 MHz, CD_2Cl_2)

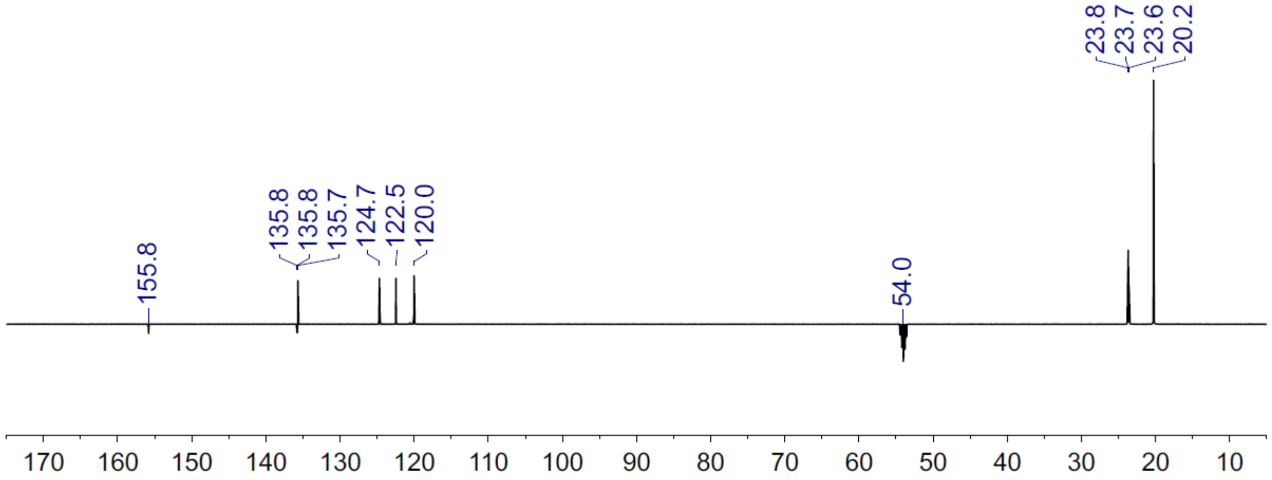

Figure S20: $^{13}C\{^1H\}$ APT NMR spectrum of **4c** (126 MHz, CD_2Cl_2)

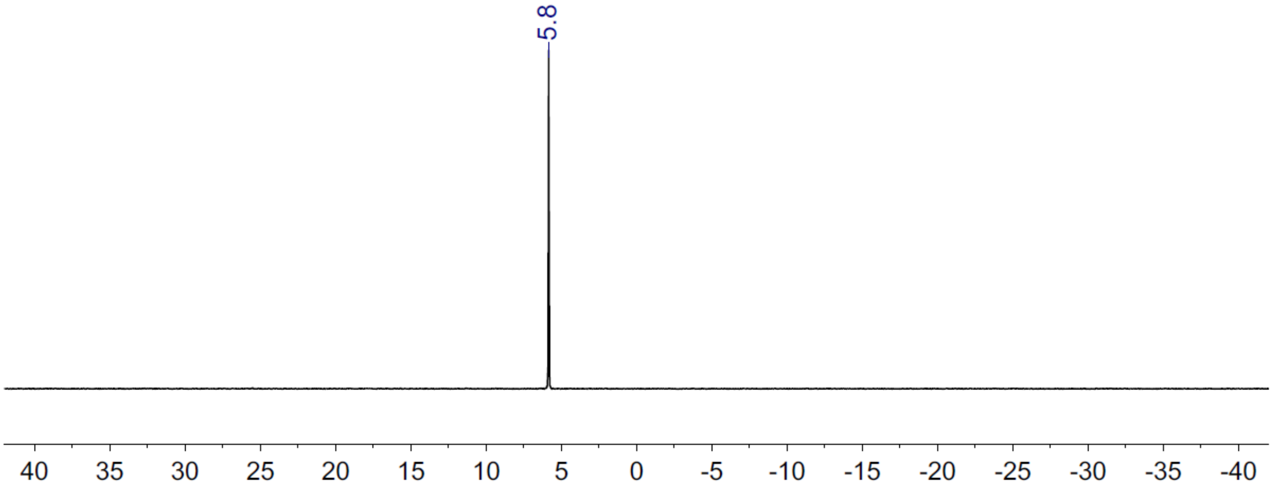

Figure S21: $^{31}P\{^1H\}$ NMR spectrum of **4c** (121 MHz, CD_2Cl_2)

1.8. [Ir(2,2'-biphenyl)(PiBu$_3$)$_2$Cl] ***4d***

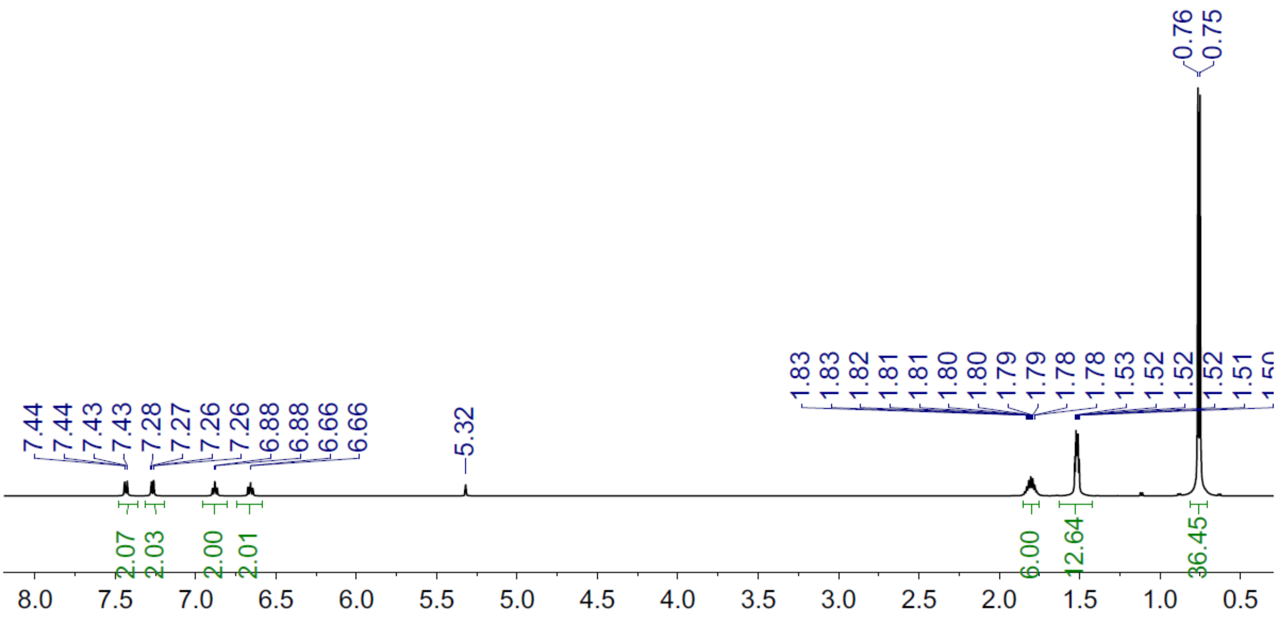

Figure S22: ^{1}H NMR spectrum of **4d** (500 MHz, CD_2Cl_2)

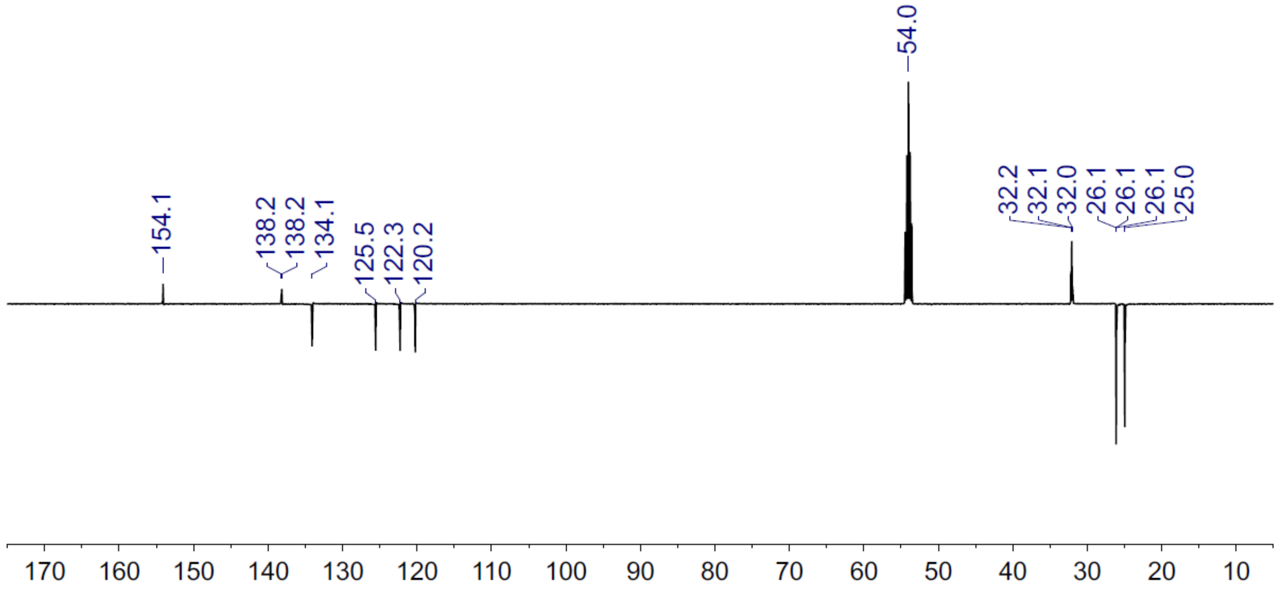

Figure S23: $^{13}C\{^{1}H\}$ APT NMR spectrum of **4d** (126 MHz, CD_2Cl_2)

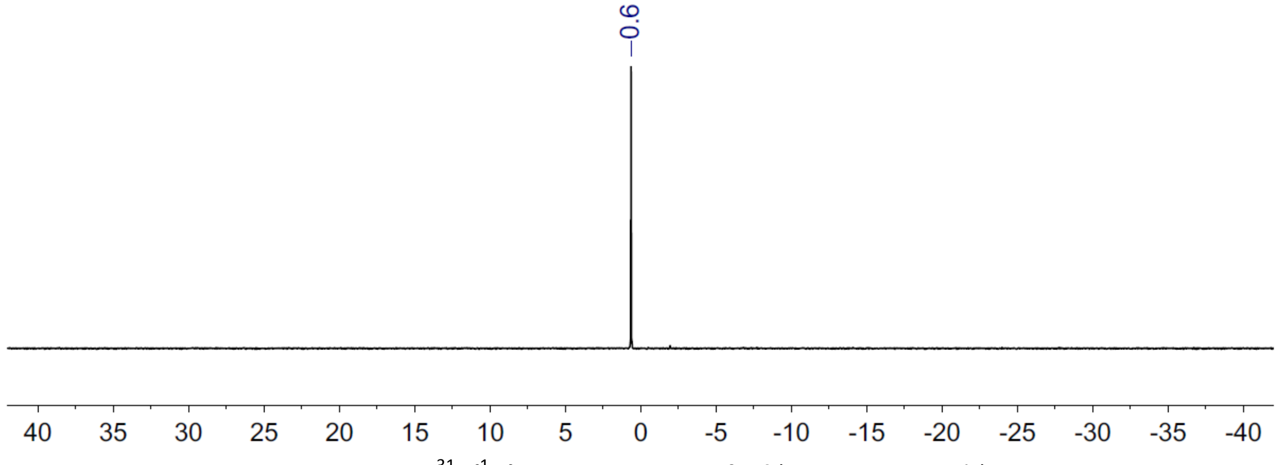

Figure S24: $^{31}P\{^{1}H\}$ NMR spectrum of **4d** (202 MHz, CD_2Cl_2)

1.9. *[Rh(2,2′-biphenyl)(PPh$_3$)$_2$][BAr$^F{}_4$]* ***1a***

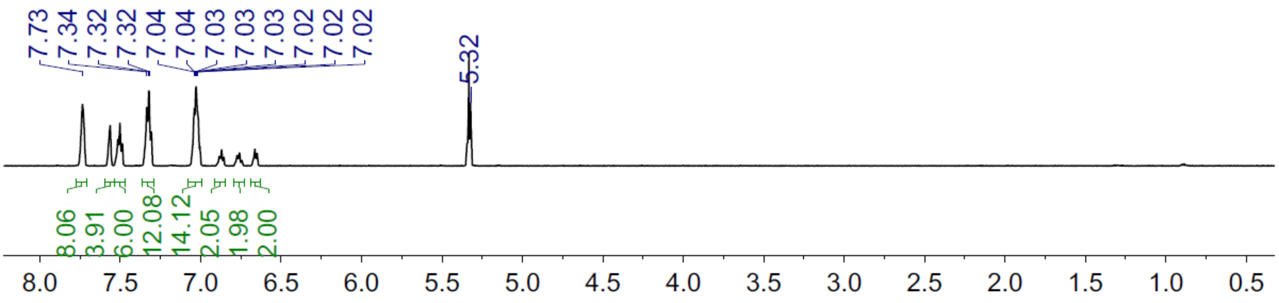

Figure S25: ^{1}H NMR spectrum of **1a** (500 MHz, CD_2Cl_2)

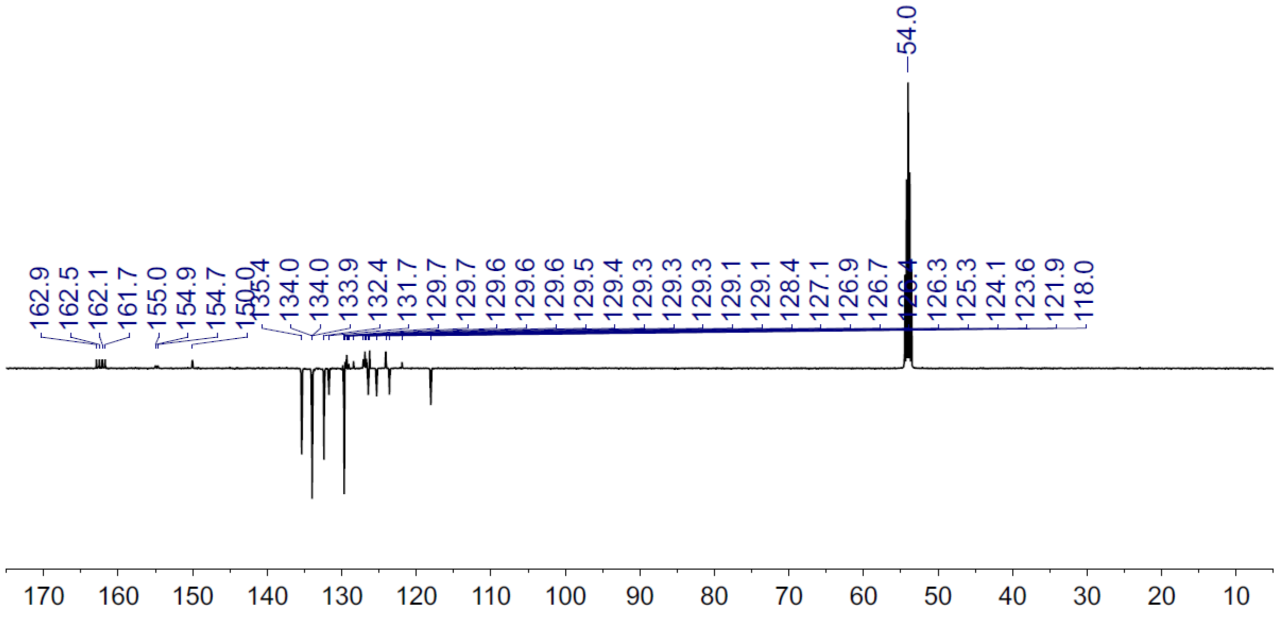

Figure S26: $^{13}C\{^1H\}$ APT NMR spectrum of **1a** (126 MHz, CD_2Cl_2)

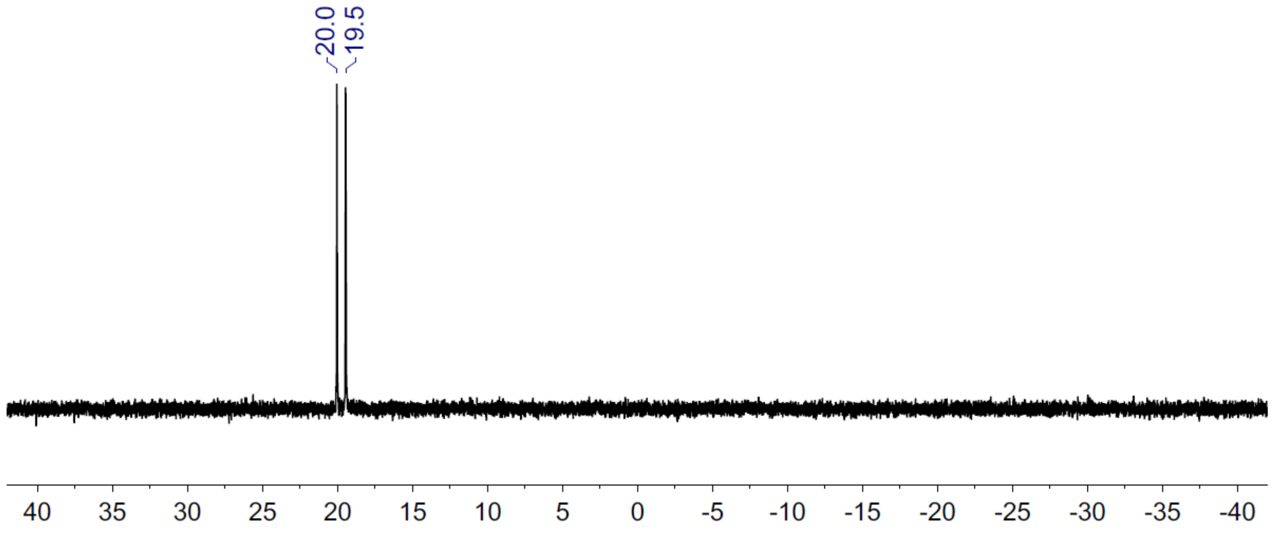

Figure S27: $^{31}P\{^1H\}$ NMR spectrum of **1a** (202 MHz, CD_2Cl_2)

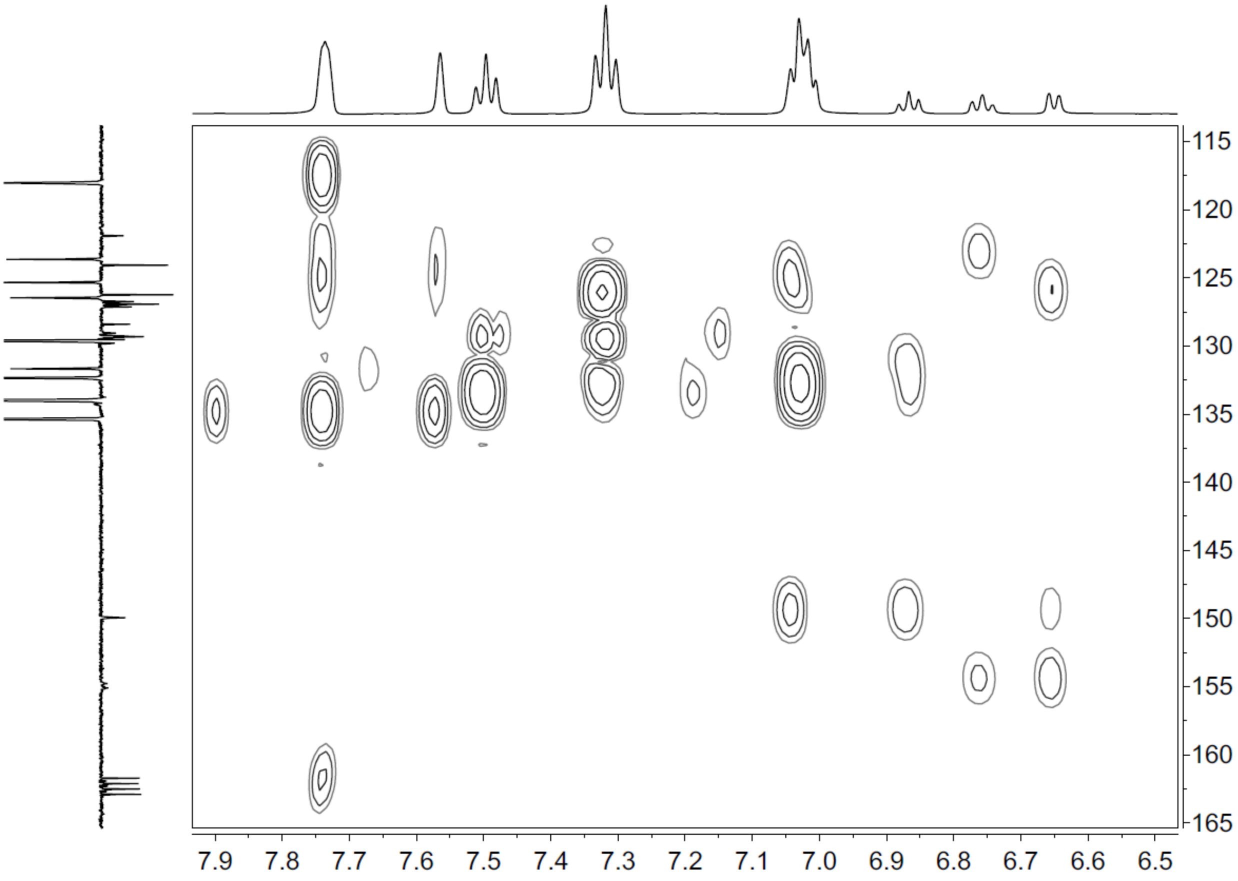

Figure S28: Section of the HMBC spectrum of **1a** (500 MHz, CD_2Cl_2)

1.10. [Rh(2,2′-biphenyl)(PCy_3)$_2$][BAr^F_4] ***1b***

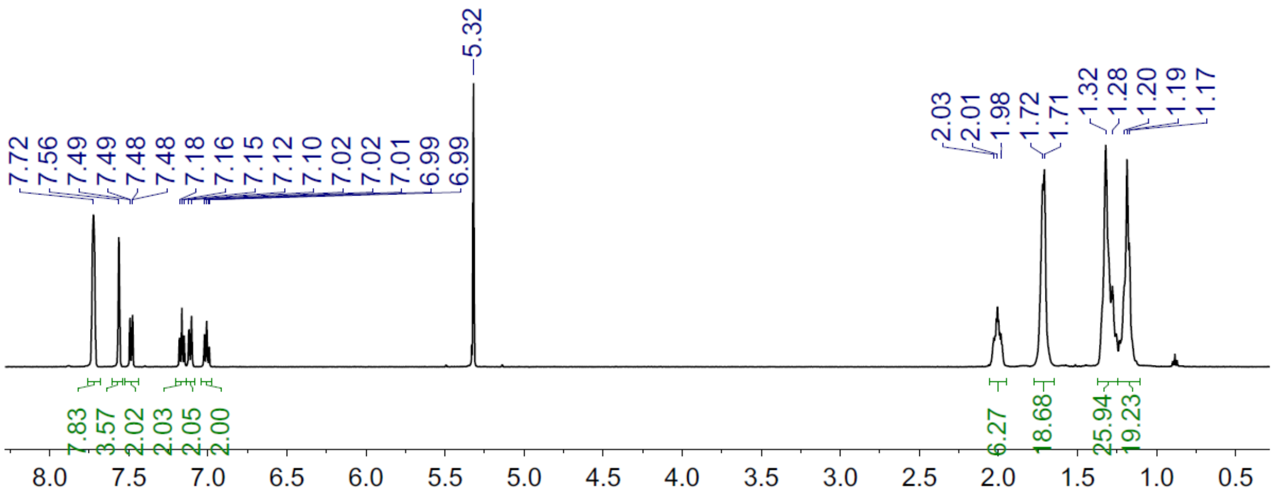

Figure S29: ^{1}H NMR spectrum of **1b** (500 MHz, CD_2Cl_2)

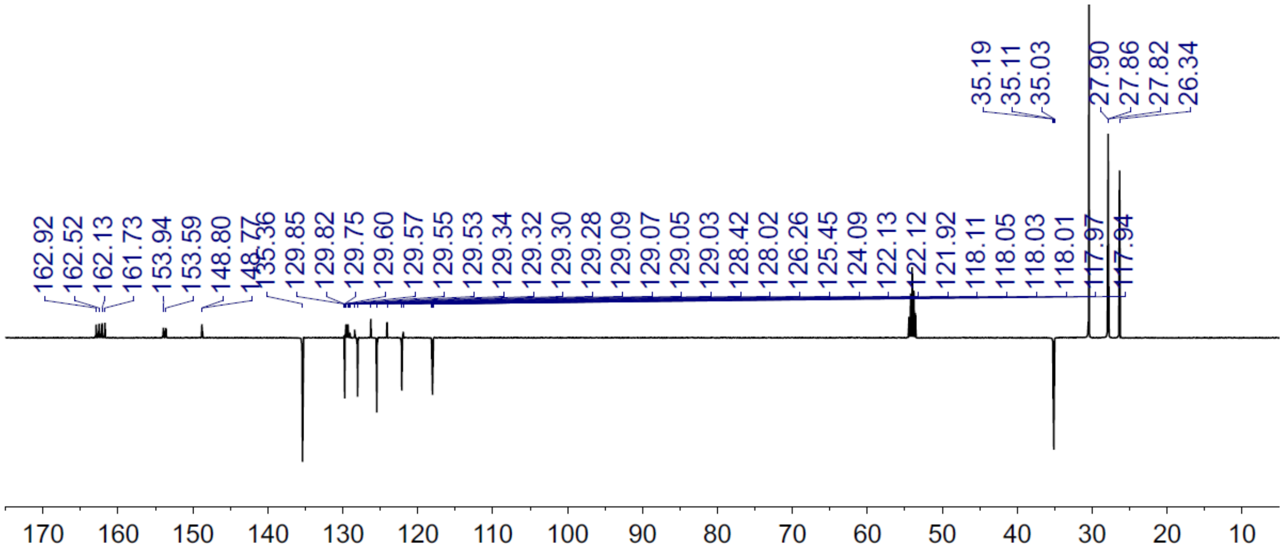

Figure S30: $^{13}C\{^{1}H\}$ APT NMR spectrum of **1b** (126 MHz, CD_2Cl_2)

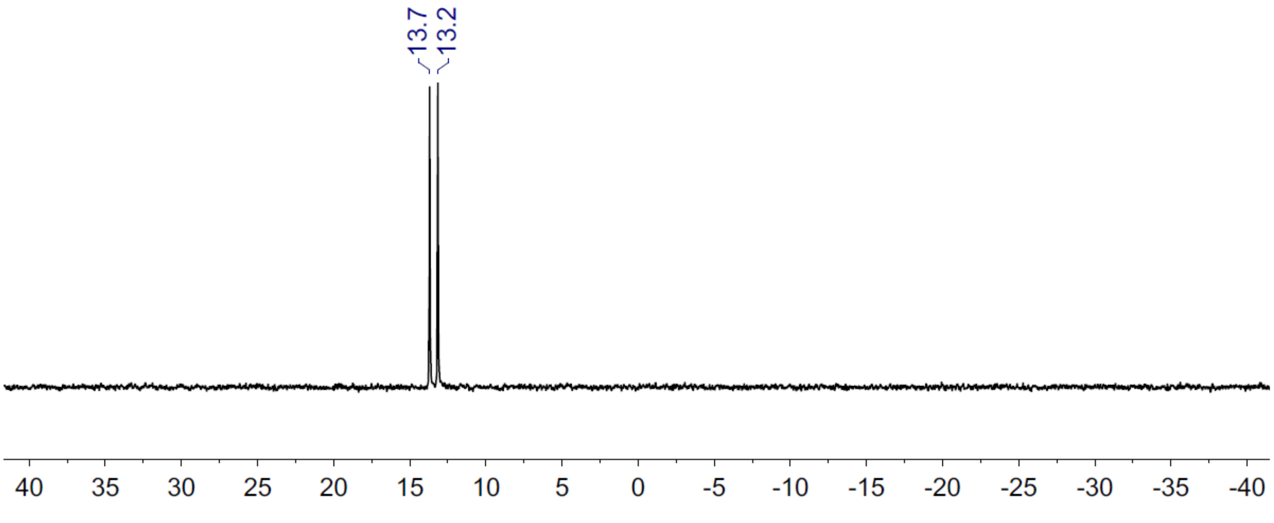

Figure S31: $^{31}P\{^{1}H\}$ NMR spectrum of **1b** (202 MHz, CD_2Cl_2)

1.11. [Rh(2,2'-biphenyl)(PiPr$_3$)$_2$][BAr^F$_4$] ***1c***

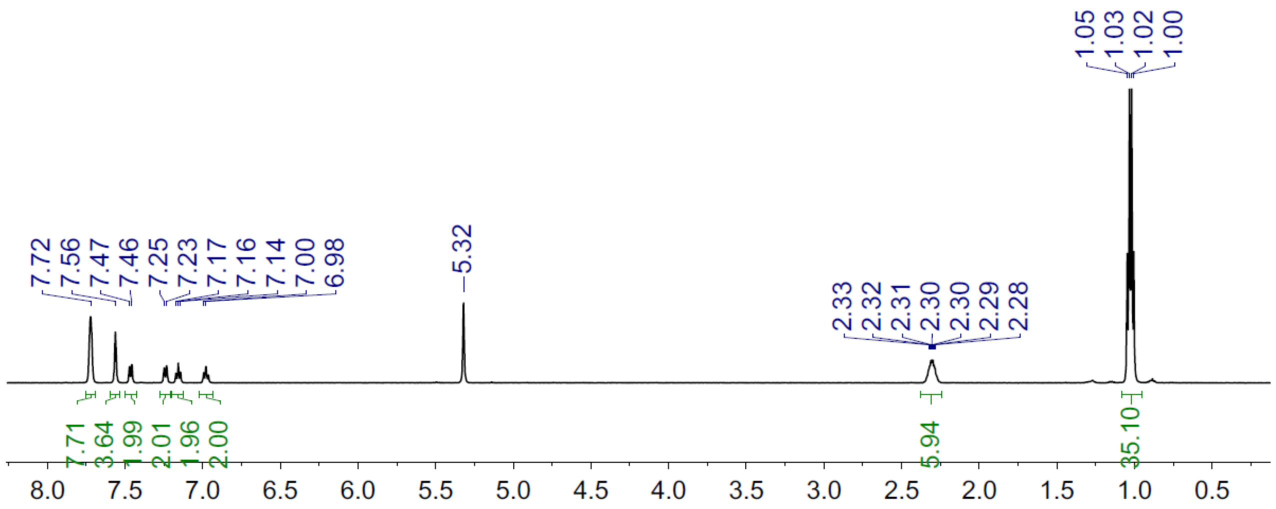

Figure S32: ^{1}H NMR spectrum of **1c** (500 MHz, CD_2Cl_2)

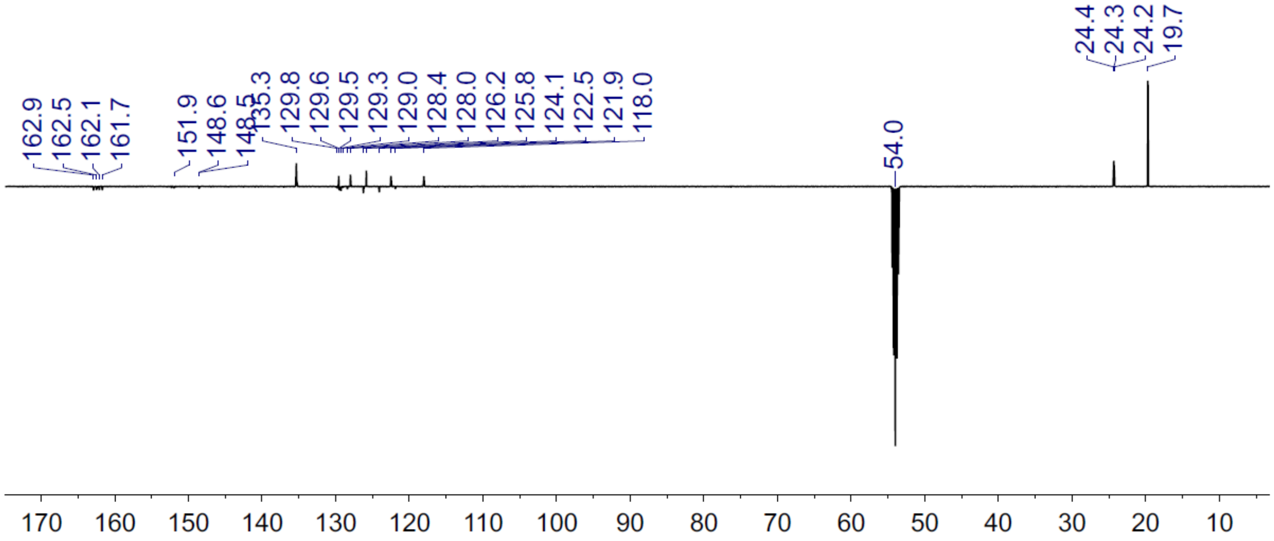

Figure S33: ^{13}C{^{1}H} APT NMR spectrum of **1c** (126 MHz, CD_2Cl_2)

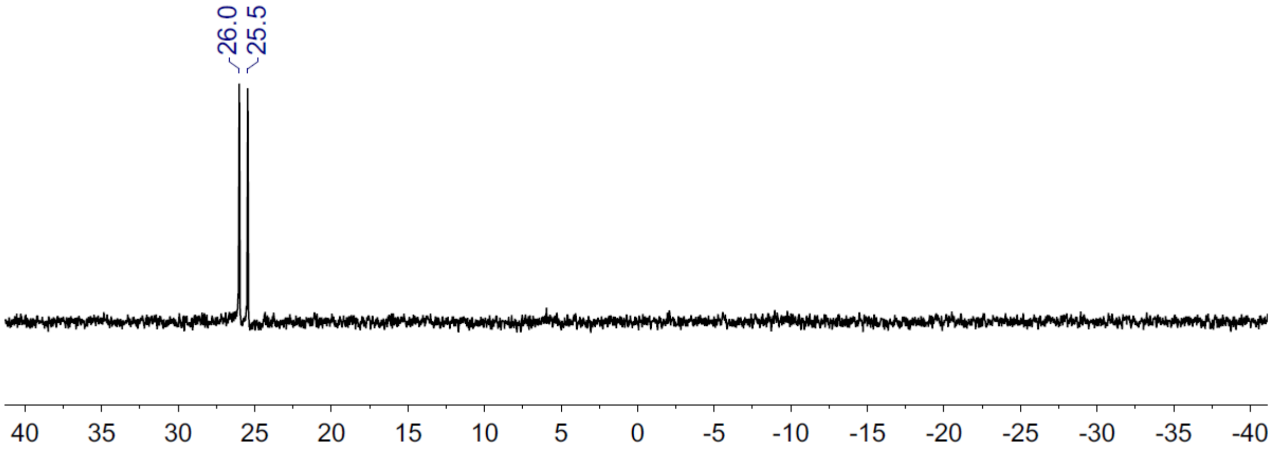

Figure S34: ^{31}P{^{1}H} NMR spectrum of **1c** (202 MHz, CD_2Cl_2)

1.12. [Rh(2,2′-biphenyl)(PiBu$_3$)$_2$][BAr$^F{}_4$] ***1d***

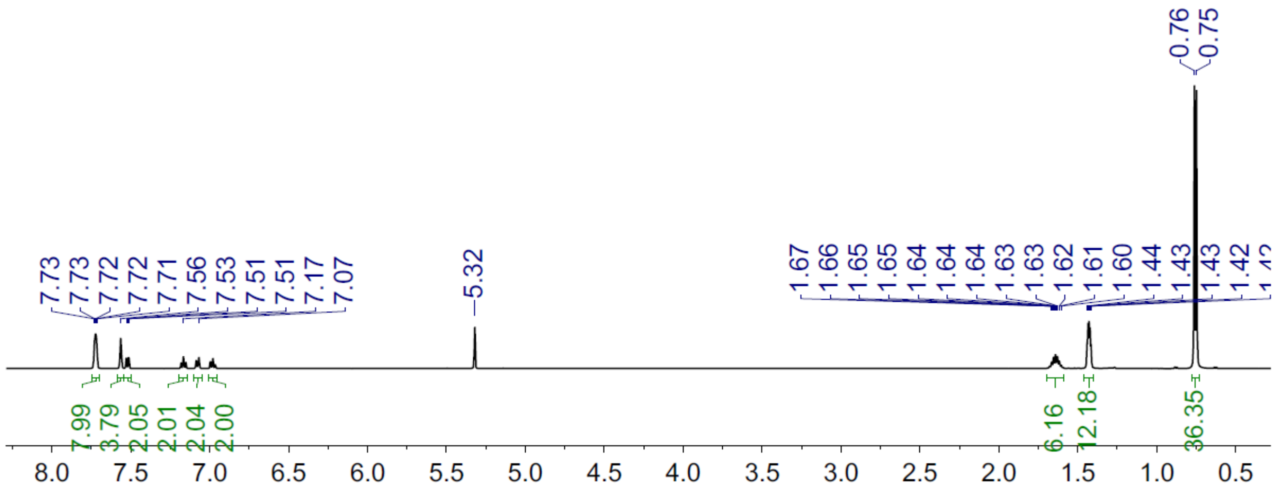

Figure S35: ^{1}H NMR spectrum of **1d** (500 MHz, CD_2Cl_2)

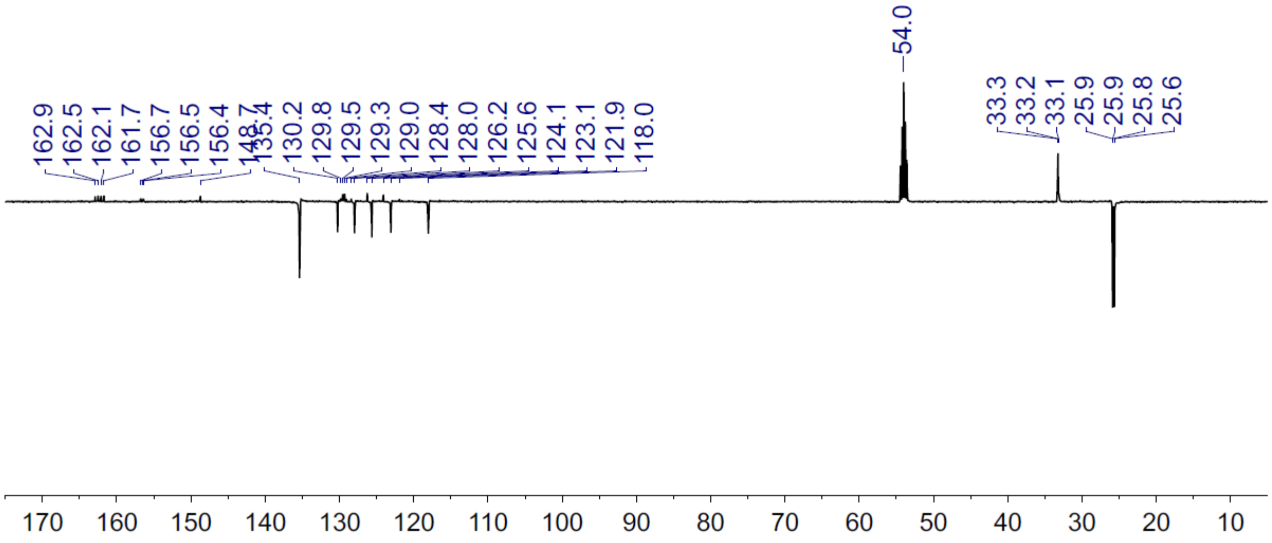

Figure S36: ^{13}C{^{1}H} APT NMR spectrum of **1d** (126 MHz, CD_2Cl_2)

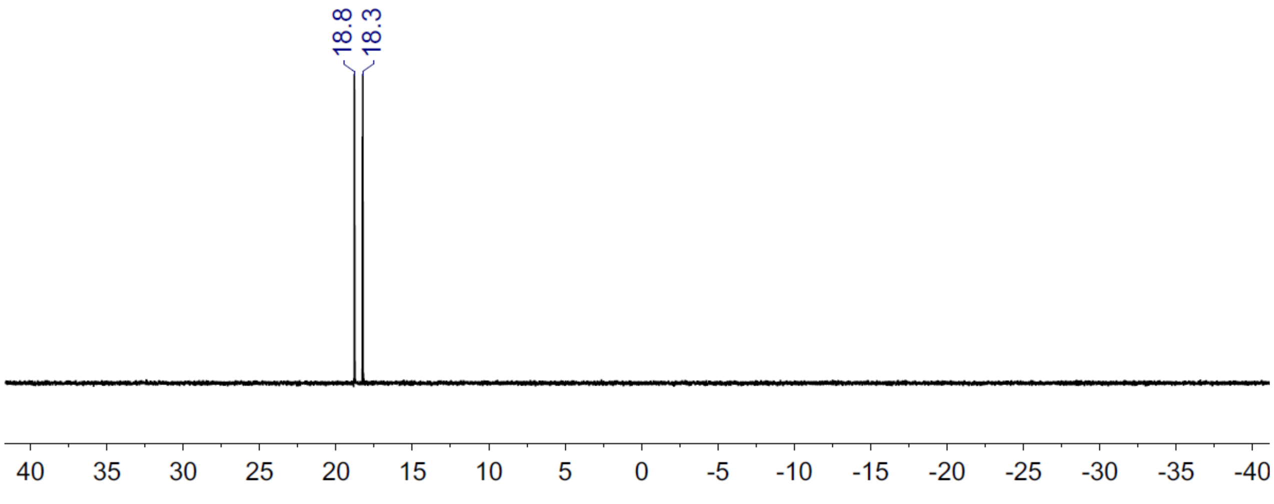

Figure S37: ^{31}P{^{1}H} NMR spectrum of **1d** (202 MHz, CD_2Cl_2)

1.13. [Rh(2,2′-biphenyl)(PiBu$_3$)$_2$][Al{OC(CF$_3$)}$_4$] ***1d****

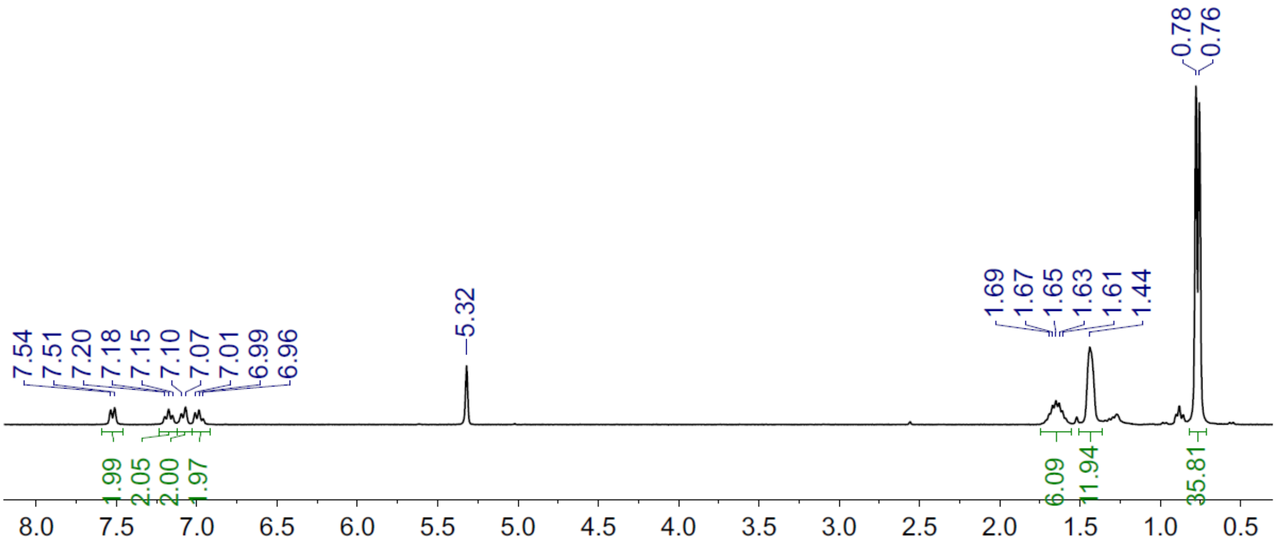

Figure S38: ^{1}H NMR spectrum of **1d*** (300 MHz, CD_2Cl_2)

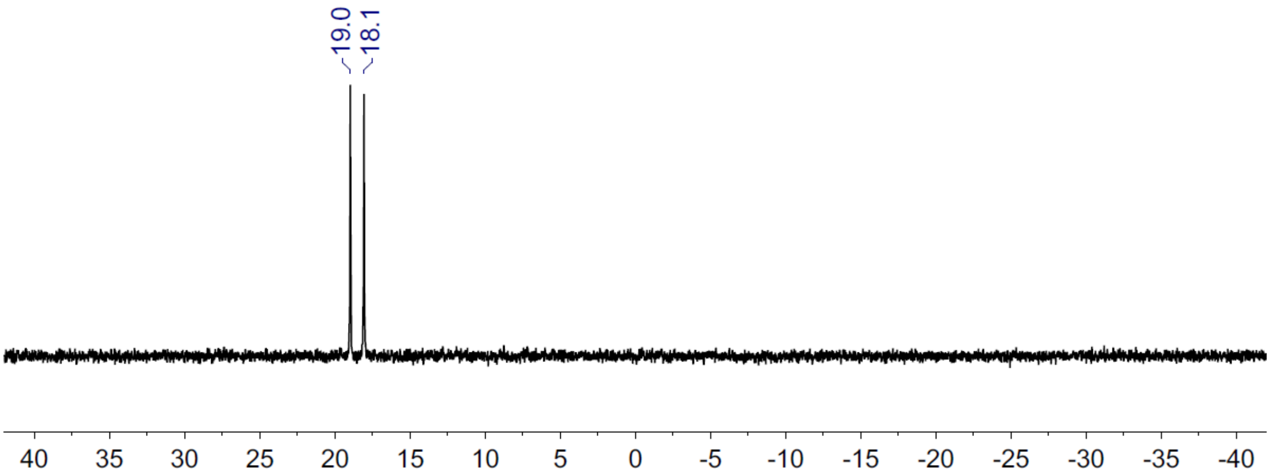

Figure S39: ^{31}P{^{1}H} NMR spectrum of **1d*** (121 MHz, CD_2Cl_2)

1.14. [Ir(2,2'-biphenyl)(PPh_3)$_2$][BAr$^F{}_4$] ***2a***

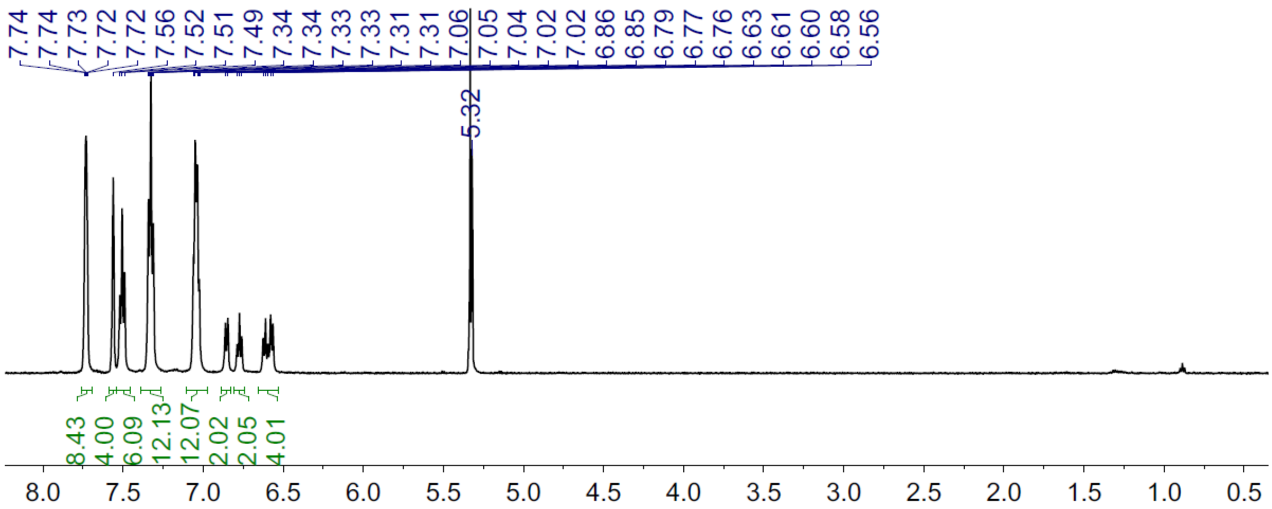

Figure S40: ^{1}H NMR spectrum of **2a** (500 MHz, CD_2Cl_2)

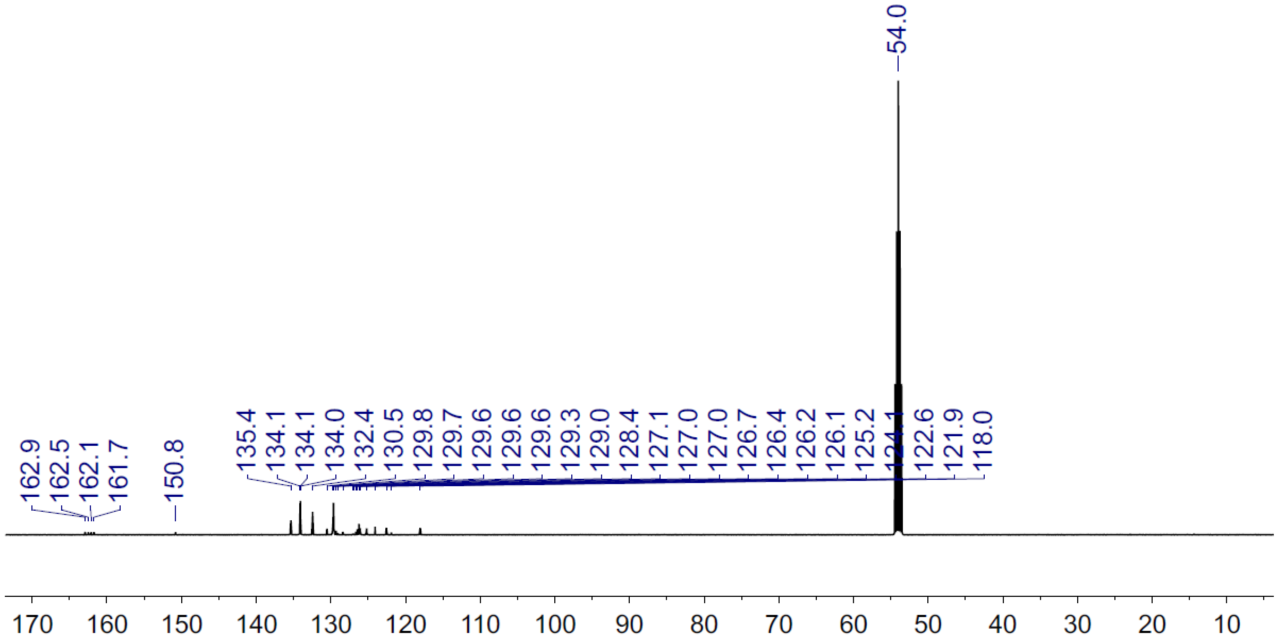

Figure S41: ^{13}C{^{1}H} NMR spectrum of **2a** (126 MHz, CD_2Cl_2)

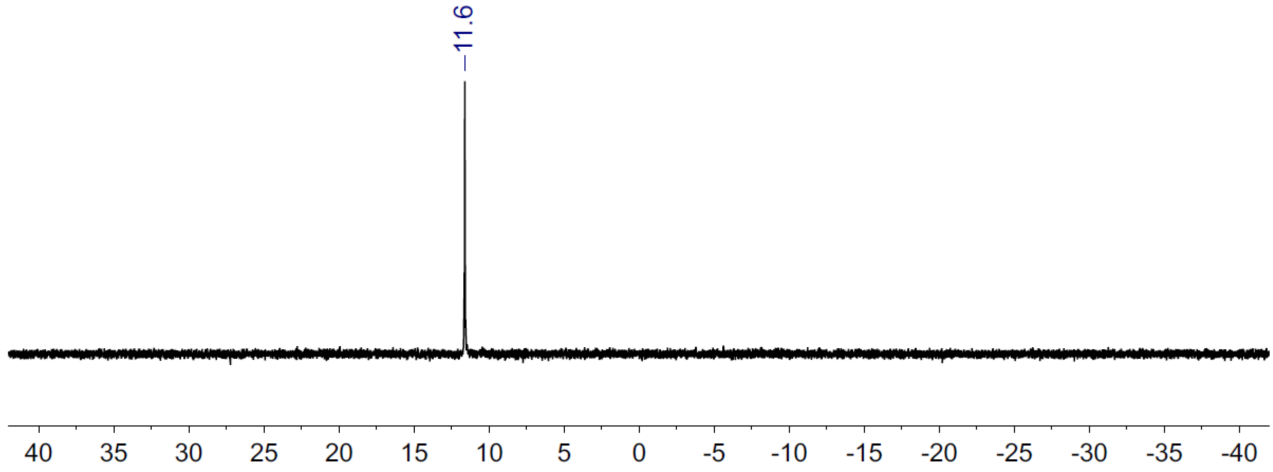

Figure S42: ^{31}P{^{1}H} NMR spectrum of **2a** (202 MHz, CD_2Cl_2)

1.15. *[Ir(2,2'-biphenyl)$(PCy_3)_2$][BAr^F_4]* ***2b***

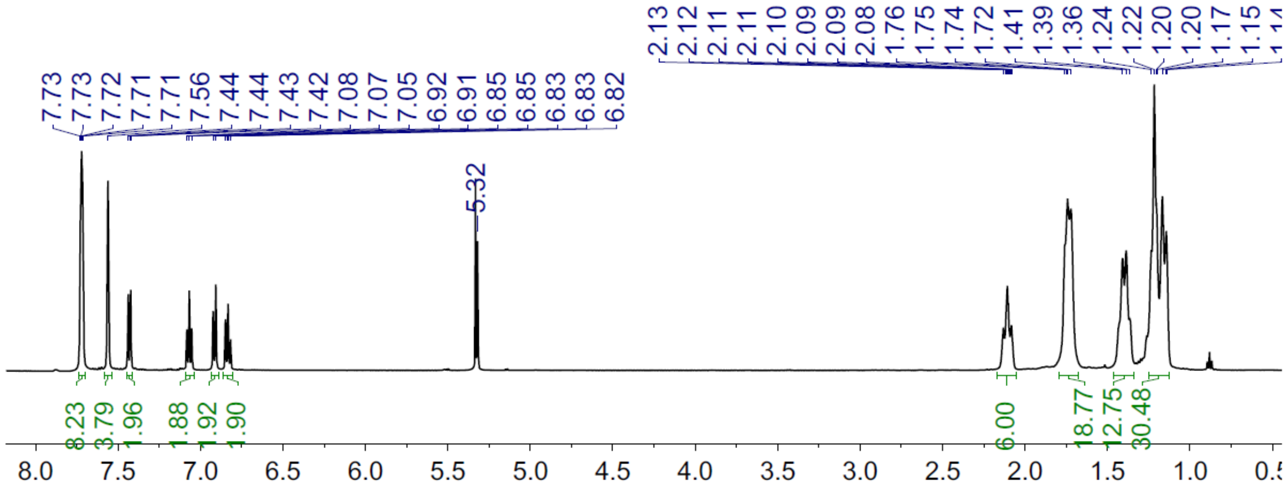

Figure S43: ^{1}H NMR spectrum of **2b** (500 MHz, CD_2Cl_2)

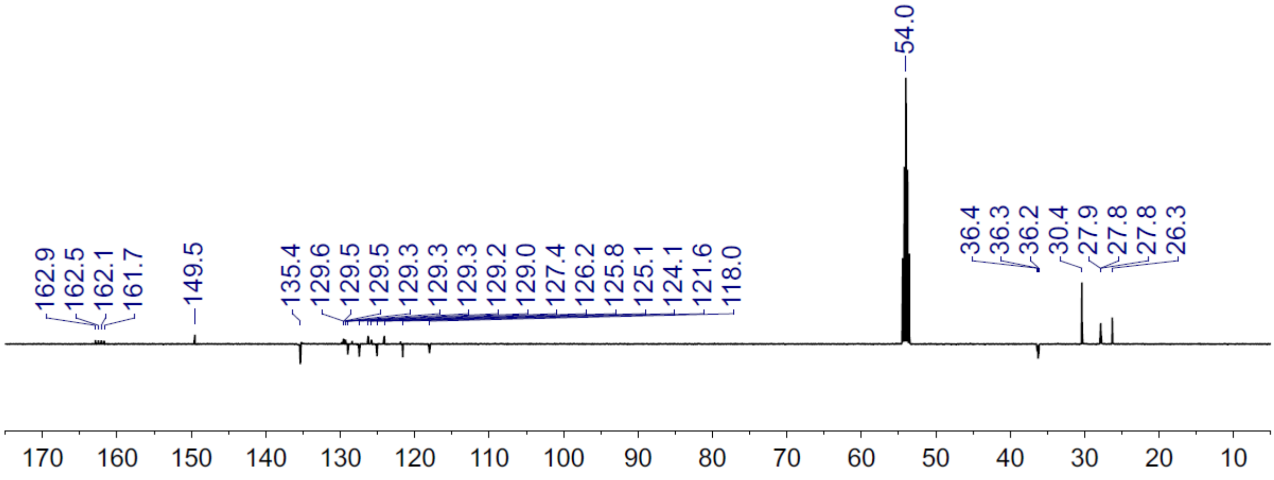

Figure S44: $^{13}C\{^{1}H\}$ APT NMR spectrum of **2b** (126 MHz, CD_2Cl_2)

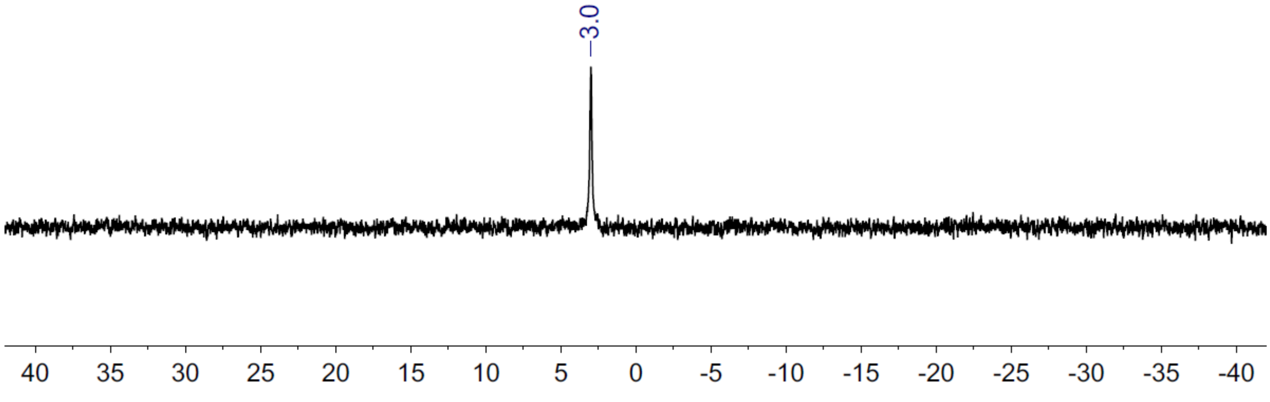

Figure S45: $^{31}P\{^{1}H\}$ NMR spectrum of **2b** (202 MHz, CD_2Cl_2)

1.16. [Ir(2,2'-biphenyl)(PiPr$_3$)$_2$][BAr$^F_4$] **2c**

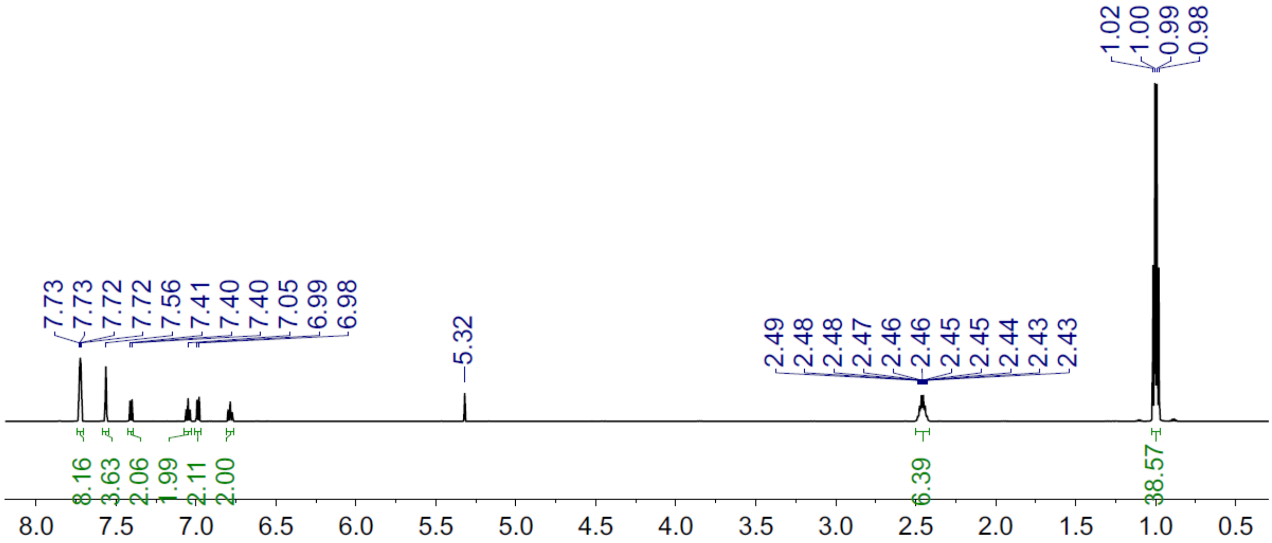

Figure S46: ^{1}H NMR spectrum of **2c** (600 MHz, CD_2Cl_2)

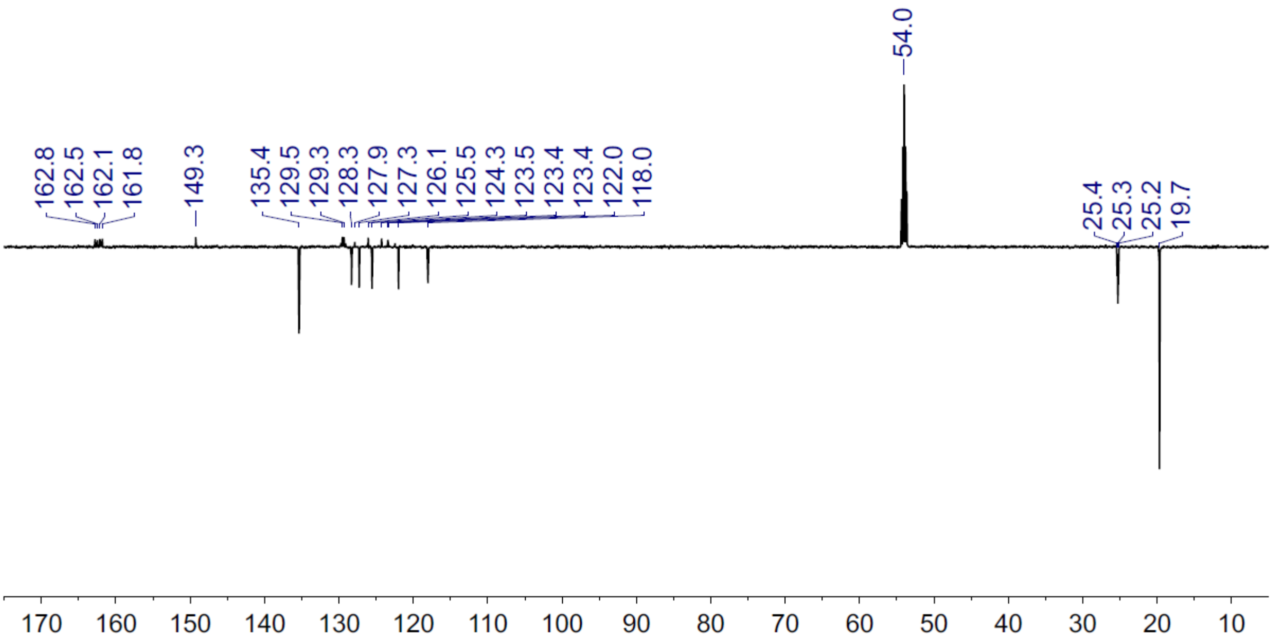

Figure S47: $^{13}C\{^1H\}$ APT NMR spectrum of **2c** (151 MHz, CD_2Cl_2)

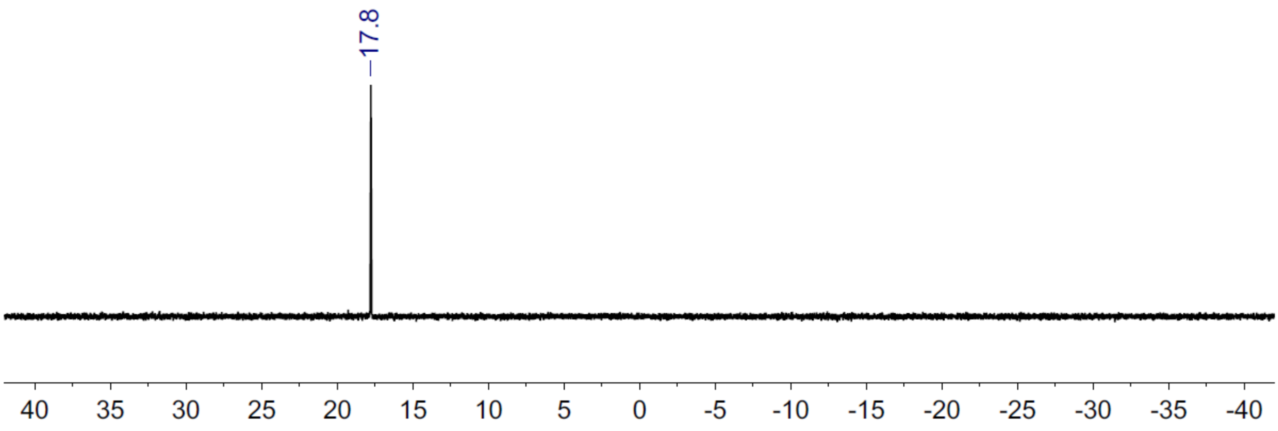

Figure S48: $^{31}P\{^1H\}$ NMR spectrum of **2c** (202 MHz, CD_2Cl_2)

1.17. [Ir(2,2′-biphenyl)(PiBu$_3$)$_2$][BAr$^F{}_4$] ***2d***

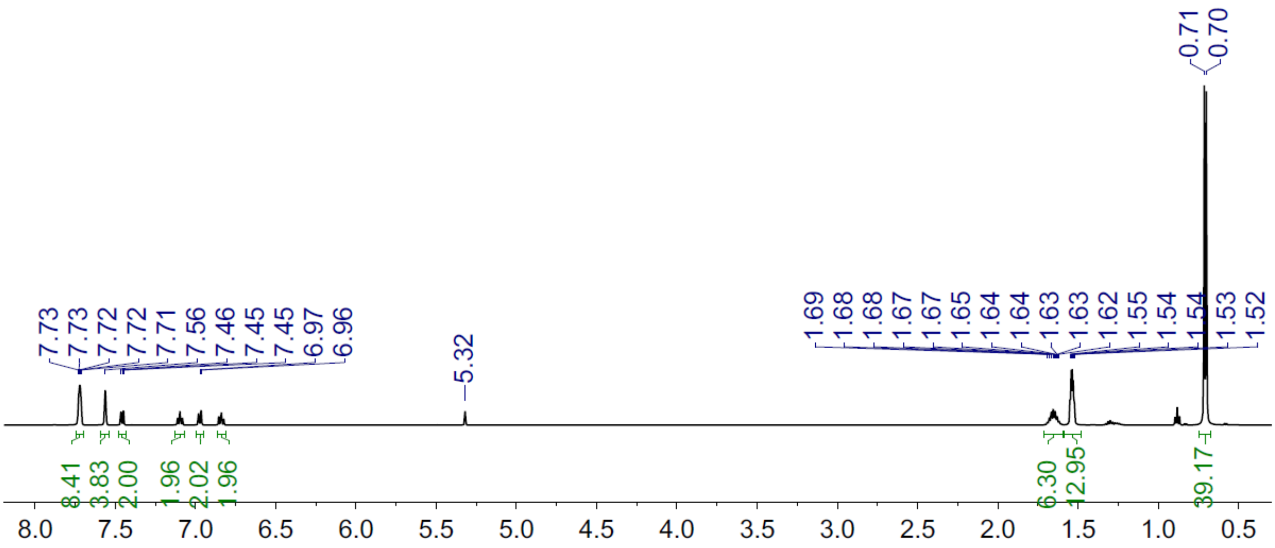

Figure S49: ^{1}H NMR spectrum of **2d** (500 MHz, CD_2Cl_2)

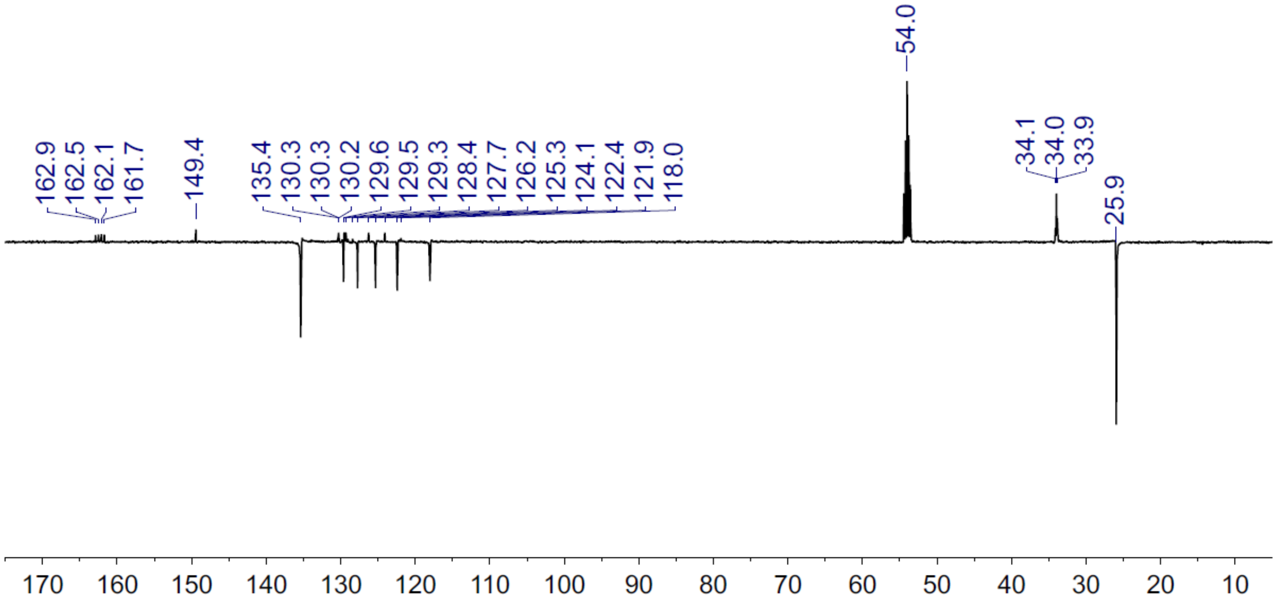

Figure S50: ^{13}C{^{1}H] APT NMR spectrum of **2d** (126 MHz, CD_2Cl_2)

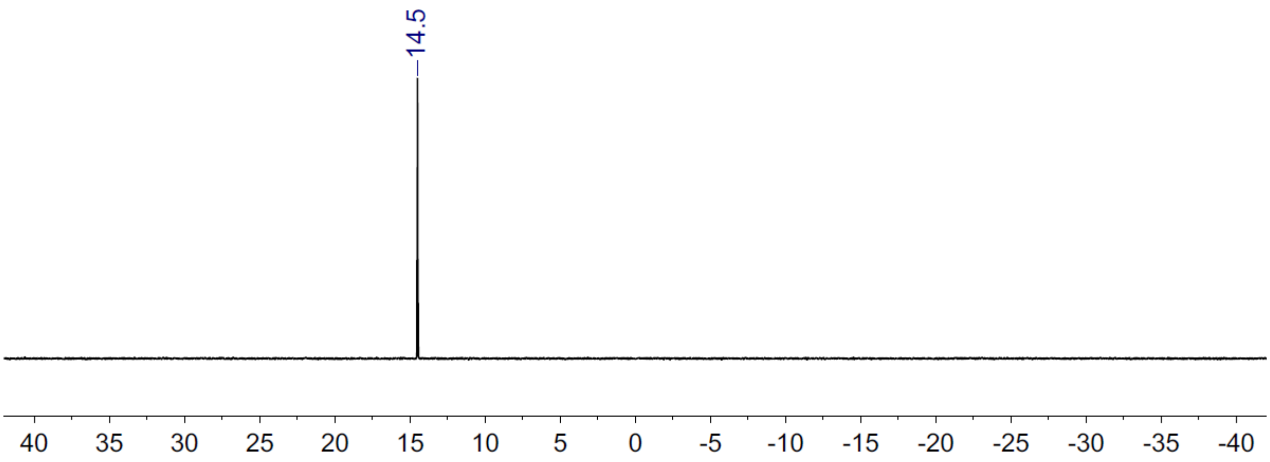

Figure S51: ^{31}P{^{1}H} NMR spectrum of **2d** (202 MHz, CD_2Cl_2)

1.18. [Ir(2,2'-biphenyl)(PiBu$_3$)$_2$][Al{OC(CF$_3$)}$_4$] ***2d****

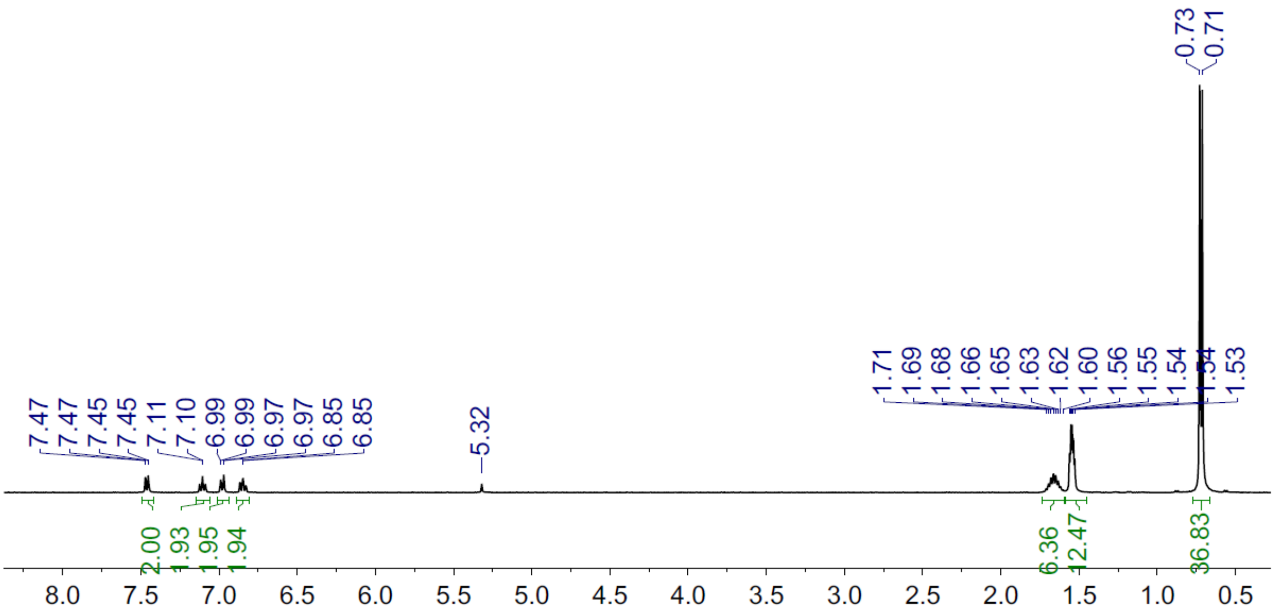

Figure S52: ^{1}H NMR spectrum of **2d*** (400 MHz, CD_2Cl_2)

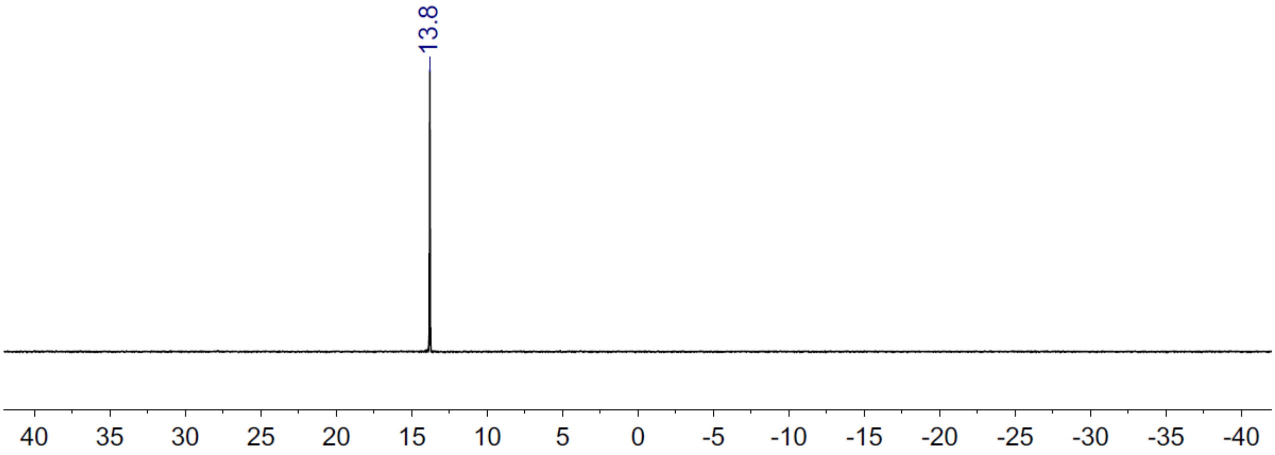

Figure S53: ^{31}P{^{1}H} NMR spectrum of **2d*** (162 MHz, CD_2Cl_2)

2. Variable temperature ^{1}H and $^{31}P\{^{1}H\}$ NMR spectra

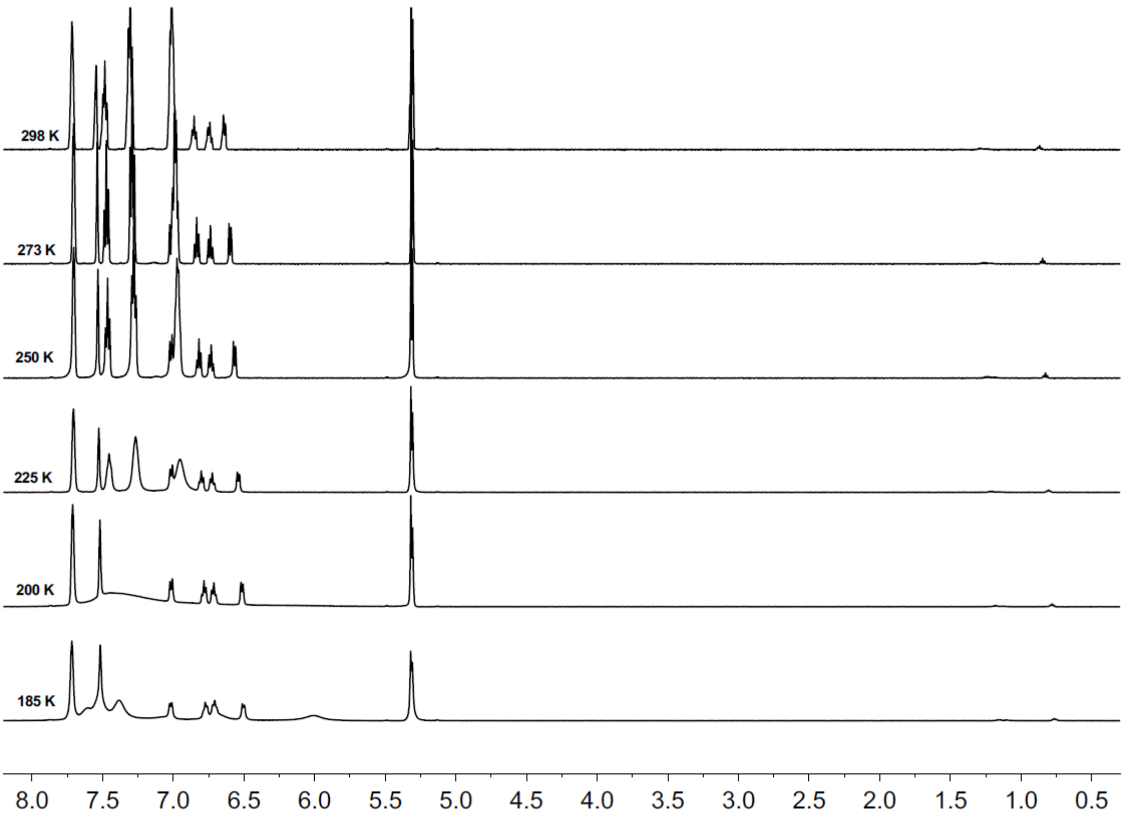

Figure S54: ^{1}H VT NMR spectra of **1a** (500 MHz, CD_2Cl_2)

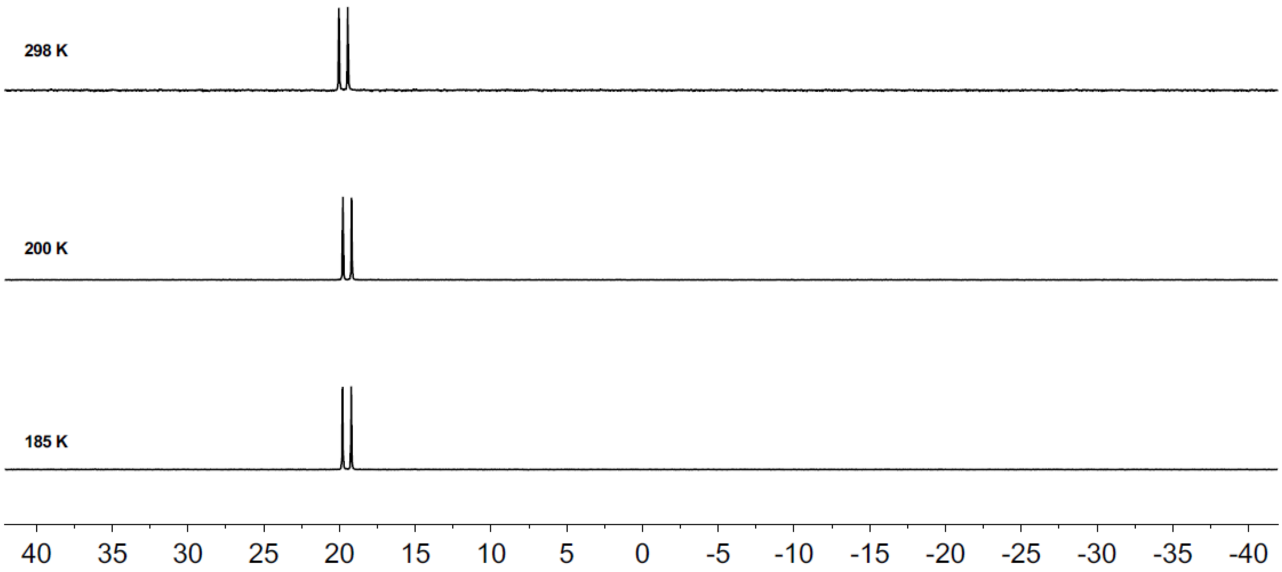

Figure S55: $^{31}P\{^{1}H\}$ VT NMR spectra of **1a** (202 MHz, CD_2Cl_2)

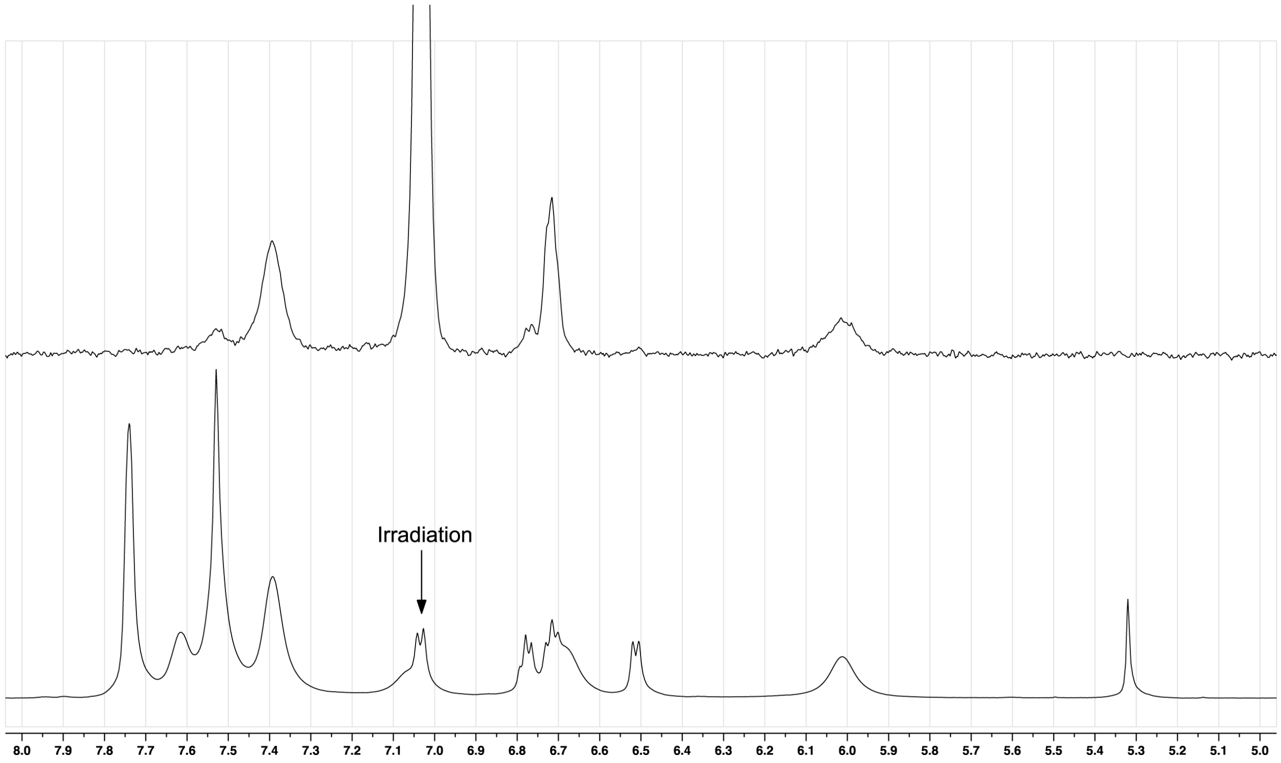

Figure S56: ^{1}H NMR spectra of **1a** at 185 K: top, with selective pre-irradiation of the δ 7.08 resonance (6/6'-biphenyl signal); bottom, reference (500 MHz, CD_2Cl_2)

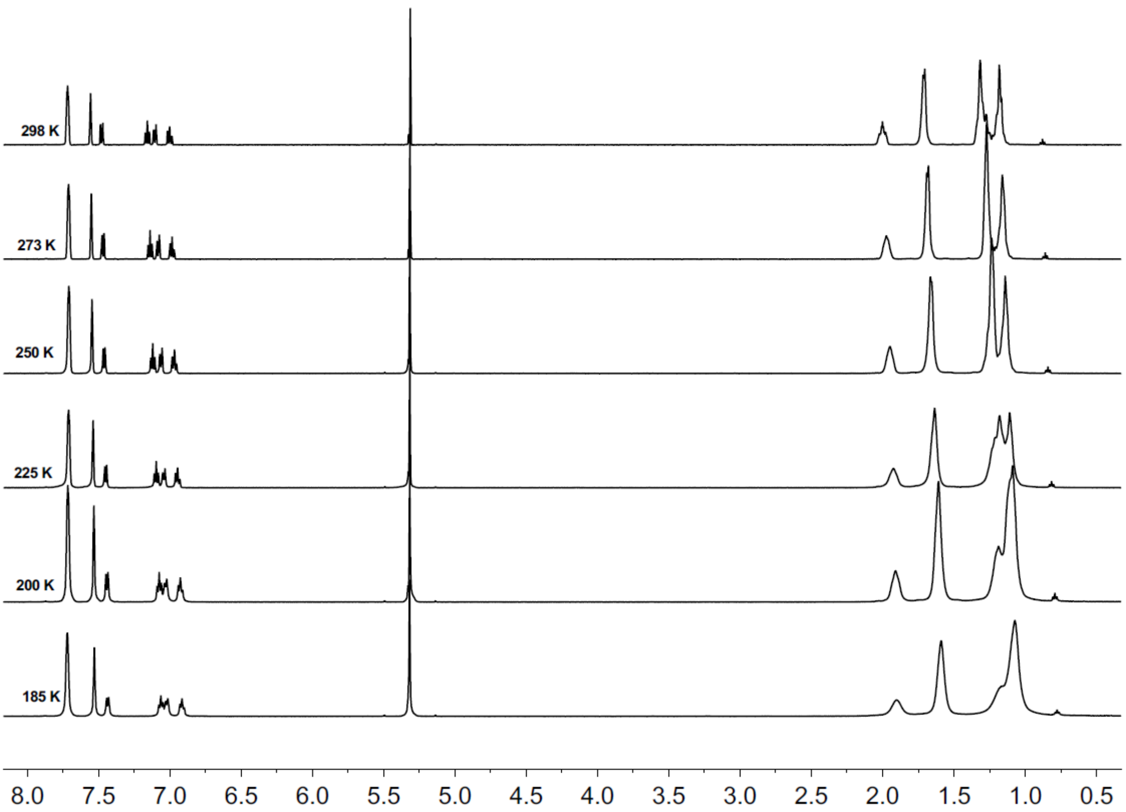

Figure S57: ^{1}H VT NMR spectra of **1b** (500 MHz, CD_2Cl_2)

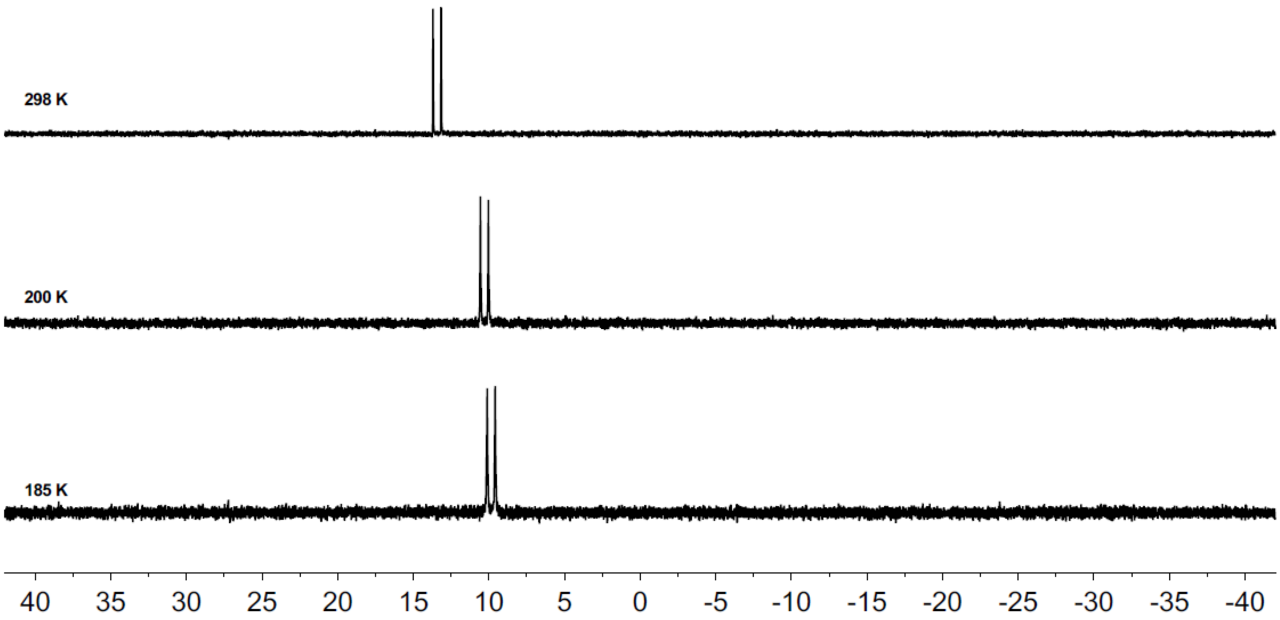

Figure S58: $^{31}P\{^{1}H\}$ VT NMR spectra of **1b** (202 MHz, CD_2Cl_2)

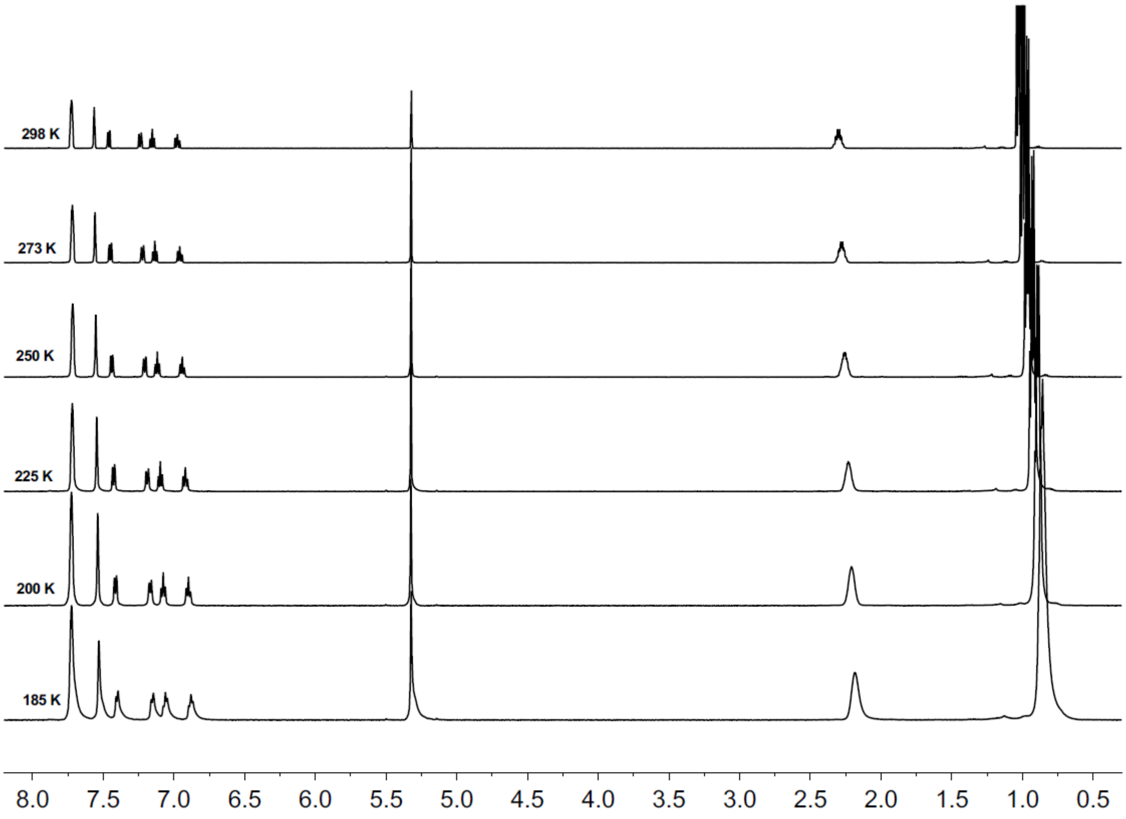

Figure S59: ^{1}H VT NMR spectra of **1c** (500 MHz, CD_2Cl_2)

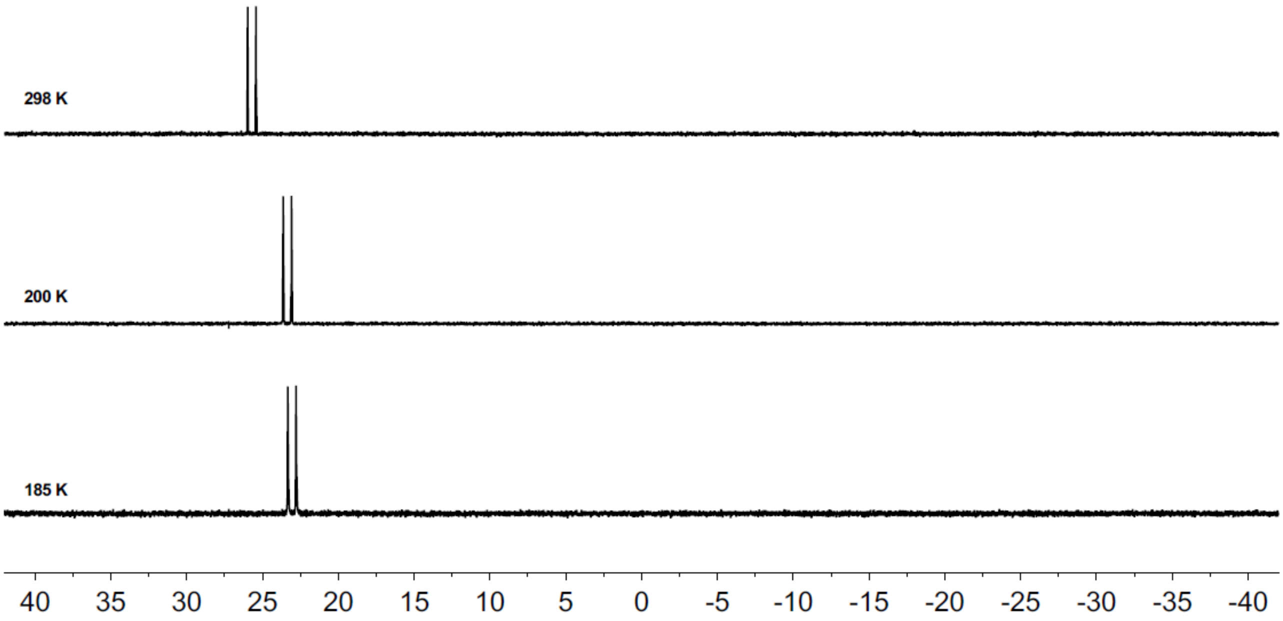

Figure S60: ^{31}P{^{1}H} VT NMR spectra of **1c** (202 MHz, CD_2Cl_2)

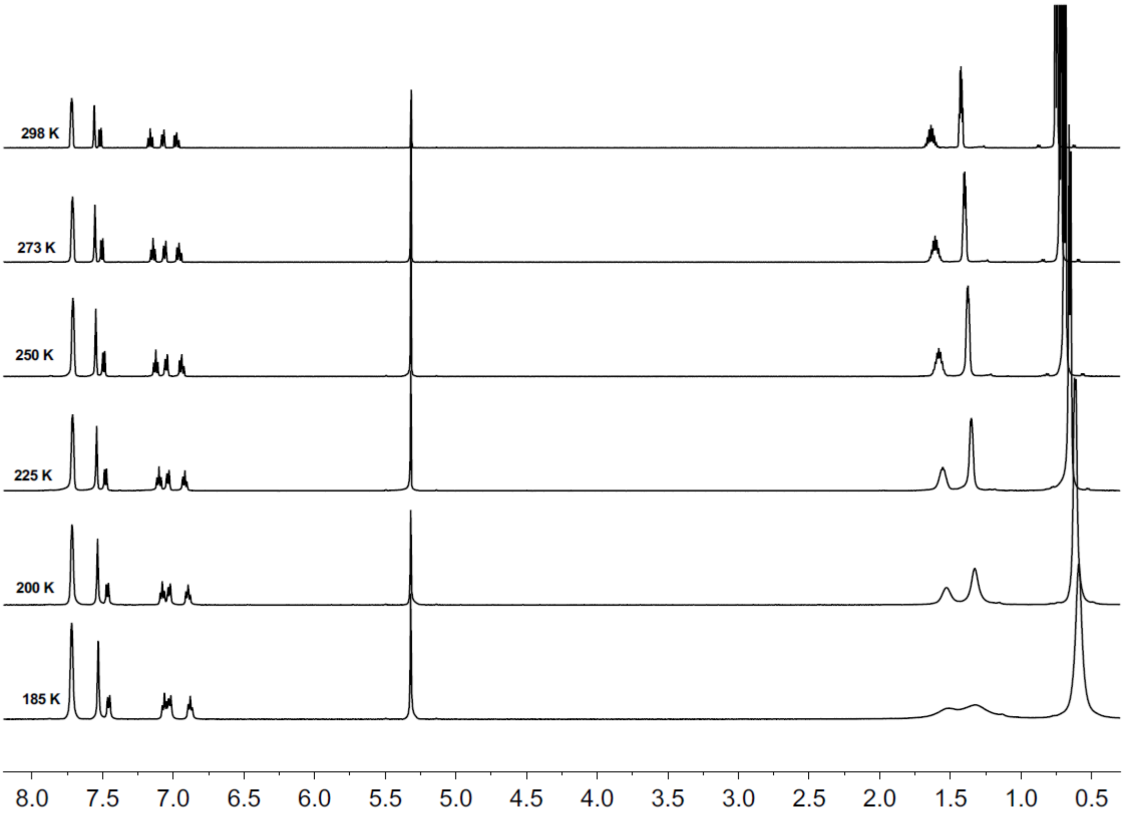

Figure S61: ^{1}H VT NMR spectra of **1d** (500 MHz, CD_2Cl_2)

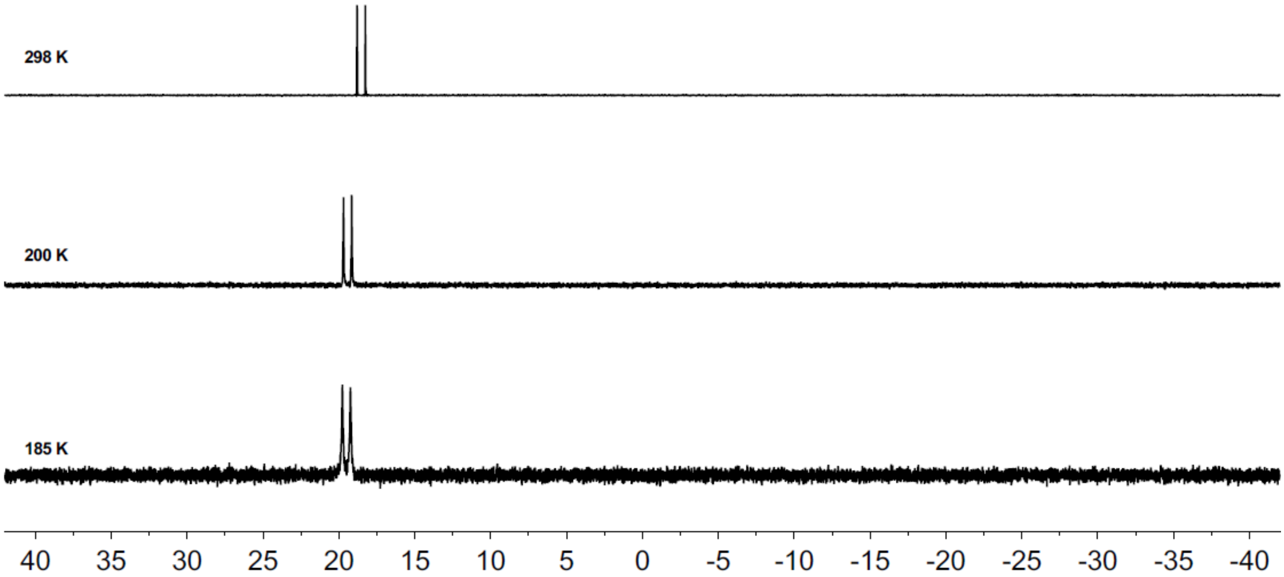

Figure S62: $^{31}P\{^{1}H\}$ VT NMR spectra of **1d** (202 MHz, CD_2Cl_2)

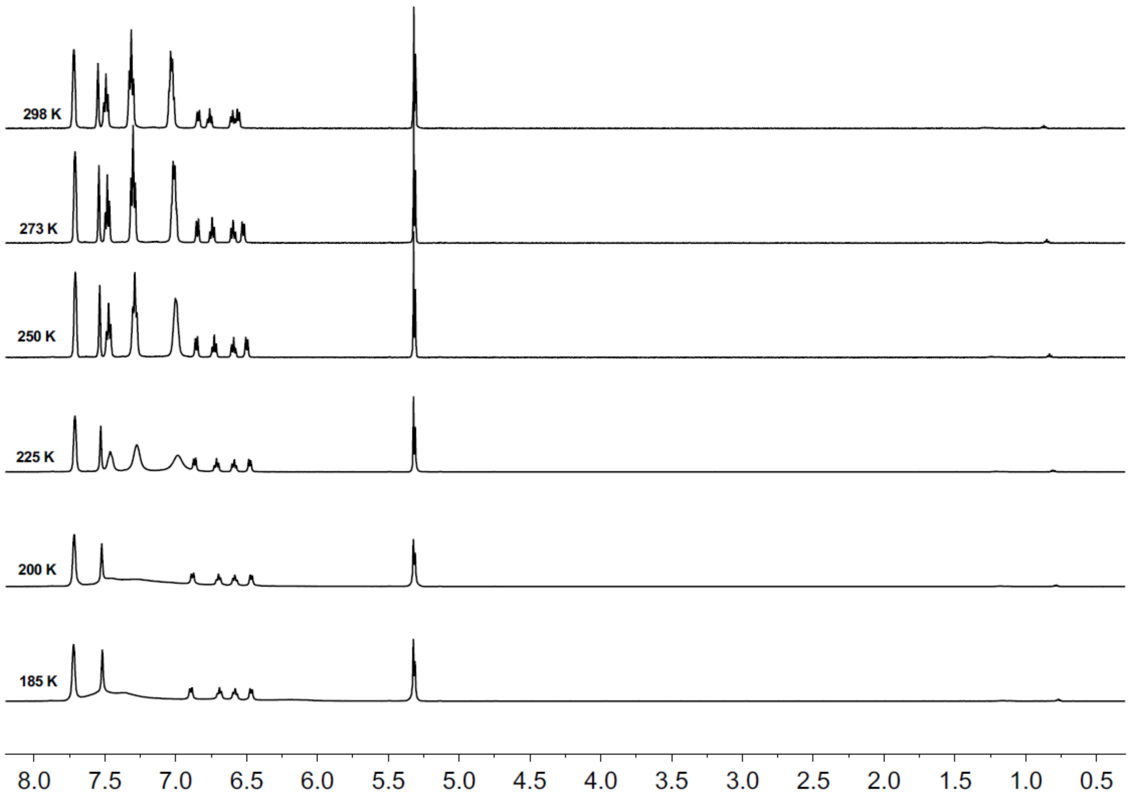

Figure S63: ^{1}H VT NMR spectra of **2a** (500 MHz, CD_2Cl_2)

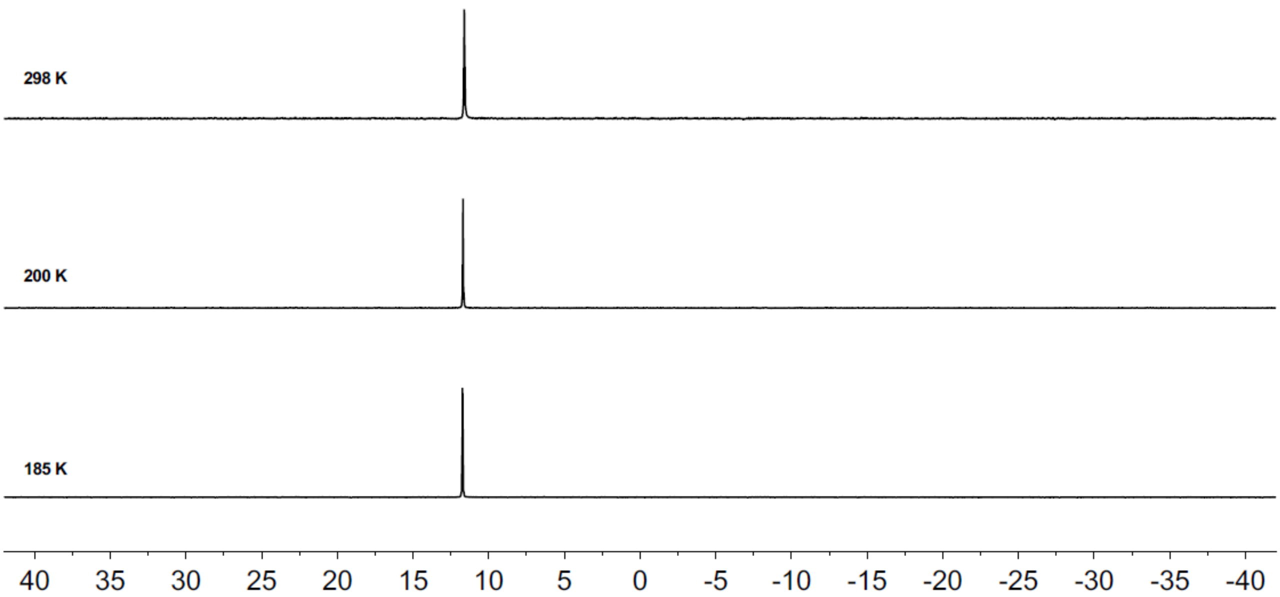

Figure S64: $^{31}P\{^{1}H\}$ VT NMR spectra of **2a** (202MHz, CD_2Cl_2)

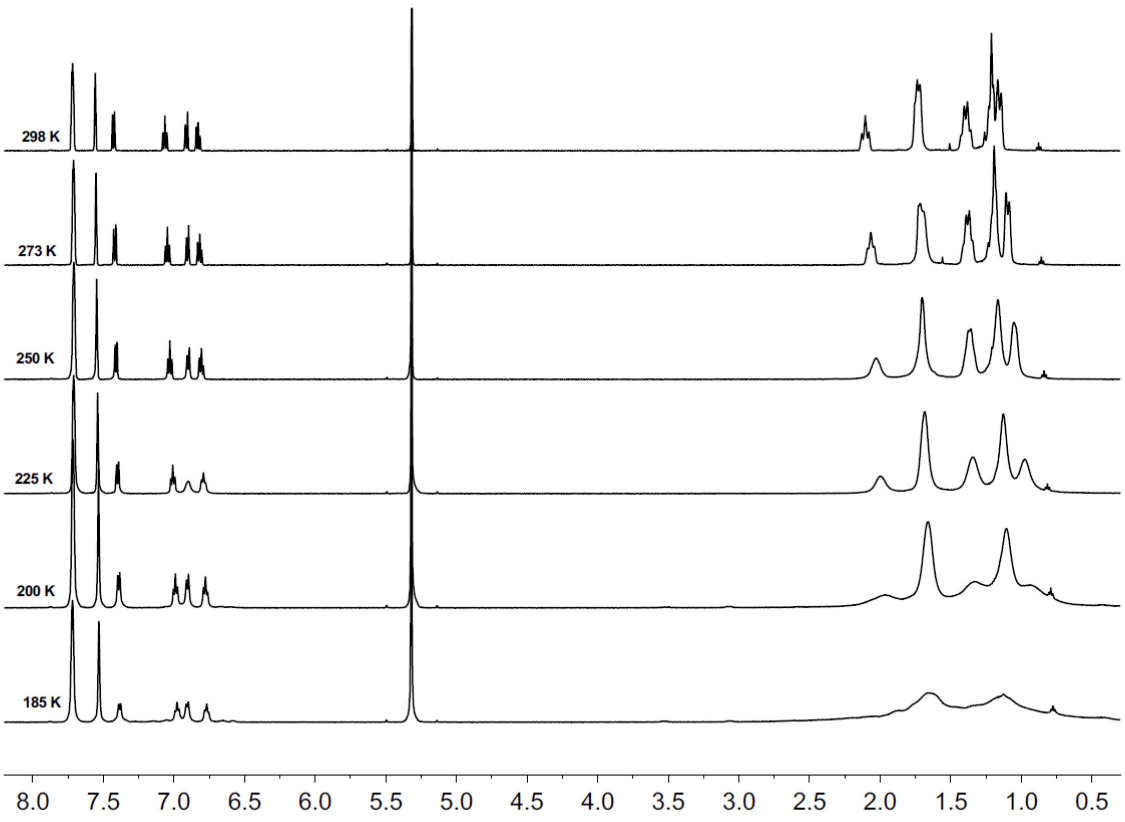

Figure S65: ^{1}H VT NMR spectra of **2b** (500 MHz, CD_2Cl_2)

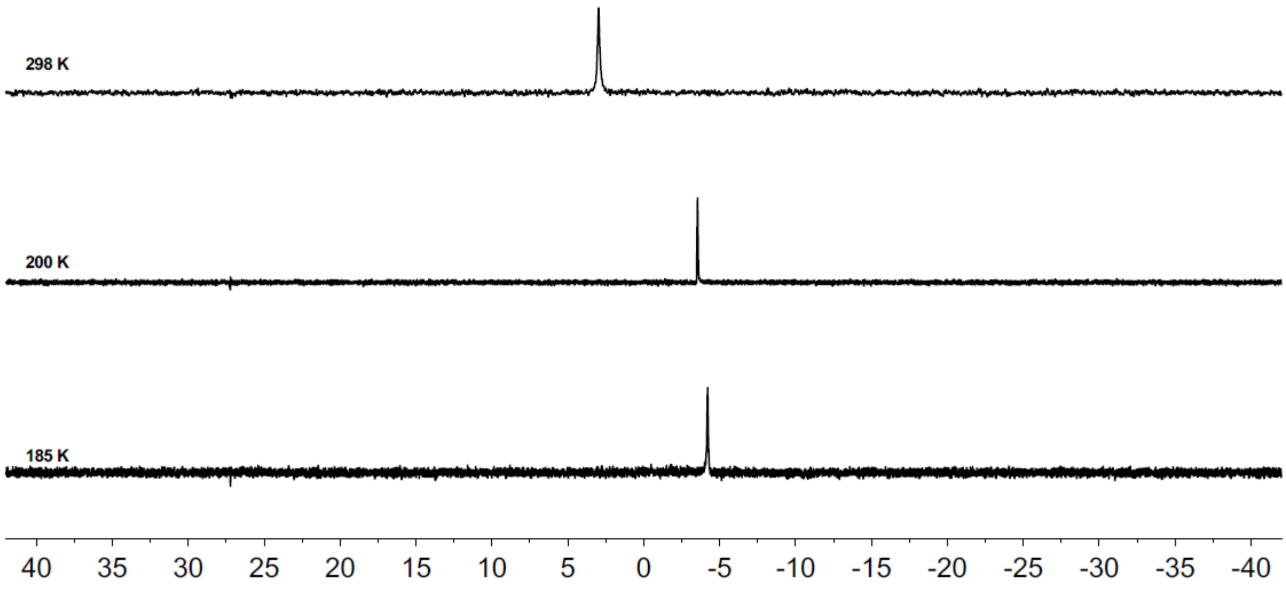

Figure S66: $^{31}P\{^1H\}$ VT NMR spectra of **2b** (202 MHz, CD_2Cl_2)

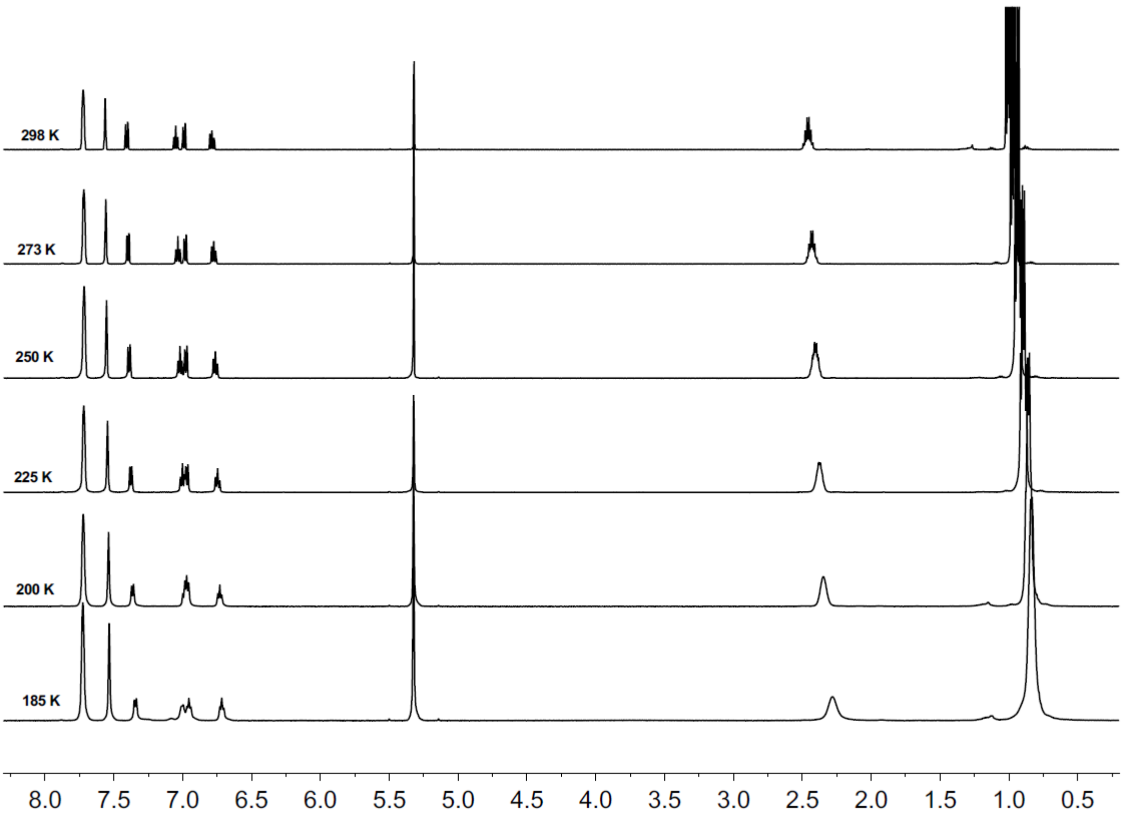

Figure S67: ^{1}H VT NMR spectra of **2c** (500 MHz, CD_2Cl_2)

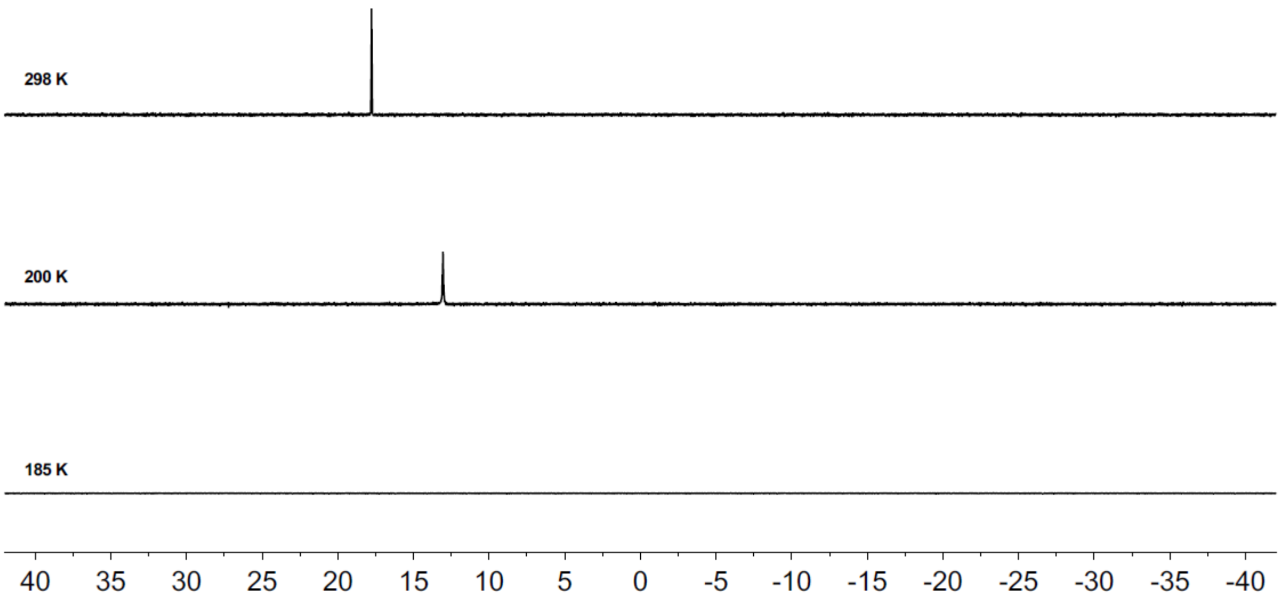

Figure S68: ^{31}P{^{1}H} VT NMR spectra of **2c** (202 MHz, CD_2Cl_2)

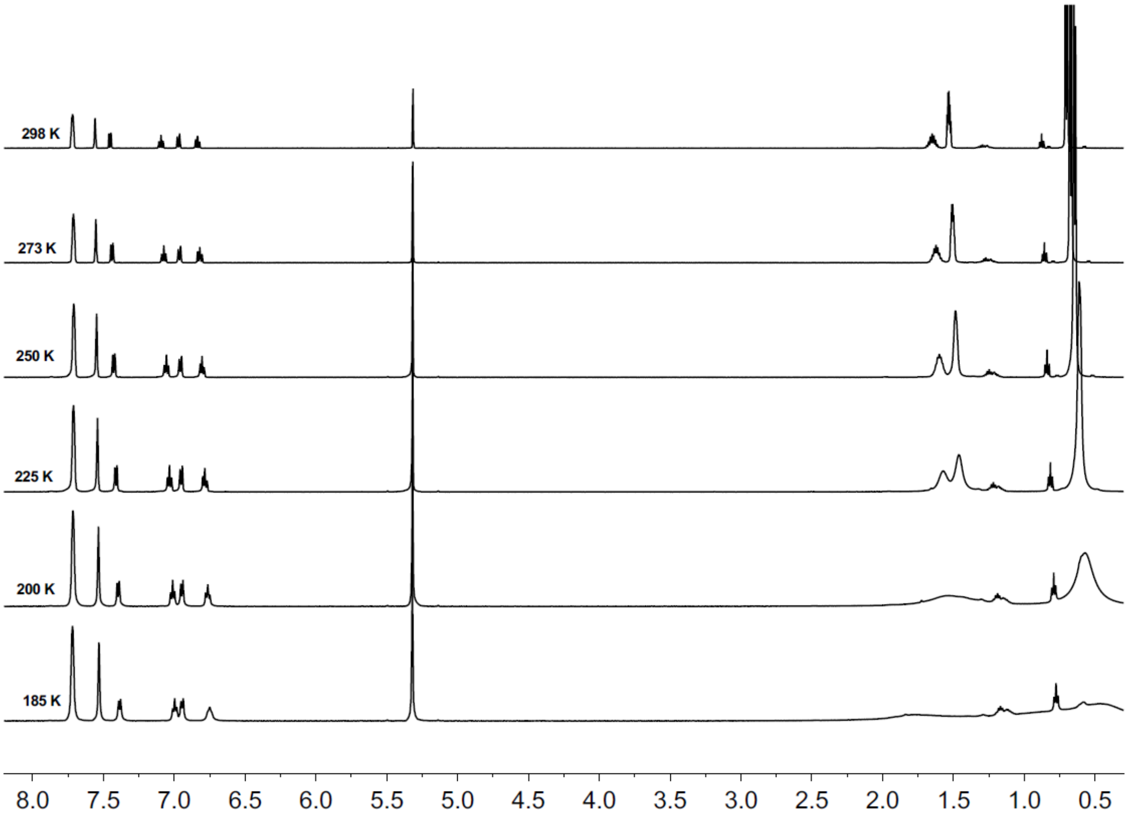

Figure S69: ^{1}H VT NMR spectra of **2d** (500 MHz, CD_2Cl_2)

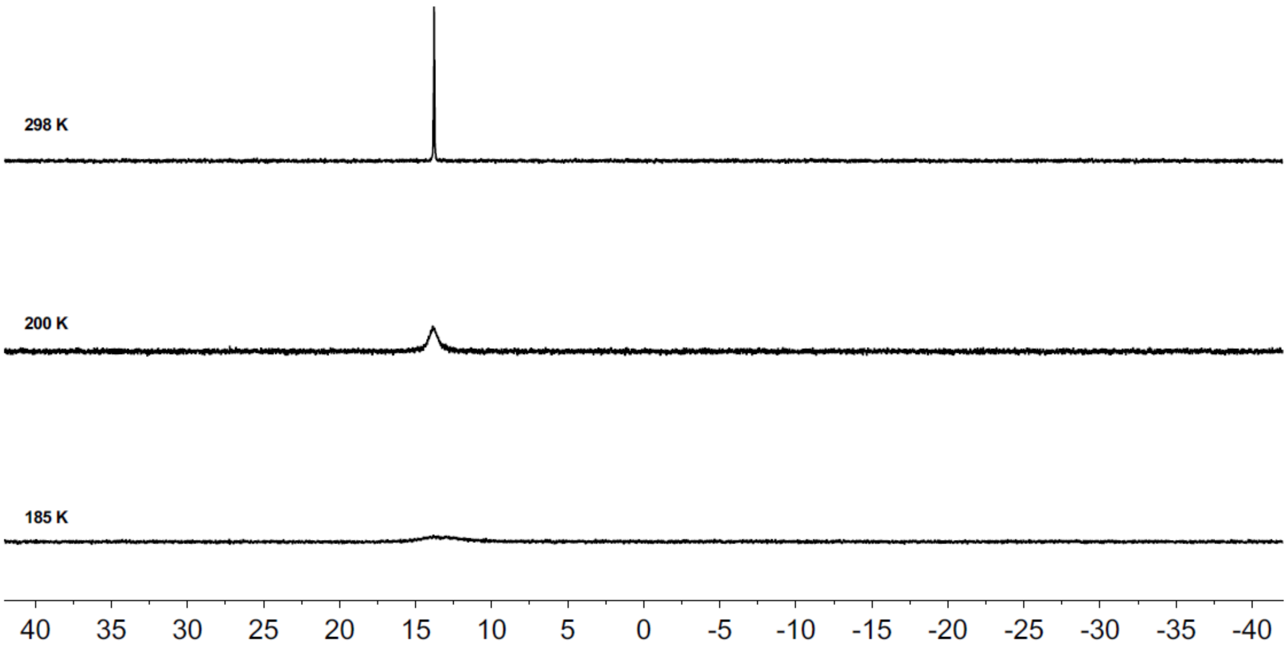

Figure S70: $^{31}P\{^{1}H\}$ VT NMR spectra of **2d** (202 MHz, CD_2Cl_2)

3. Selected HR ESI-MS

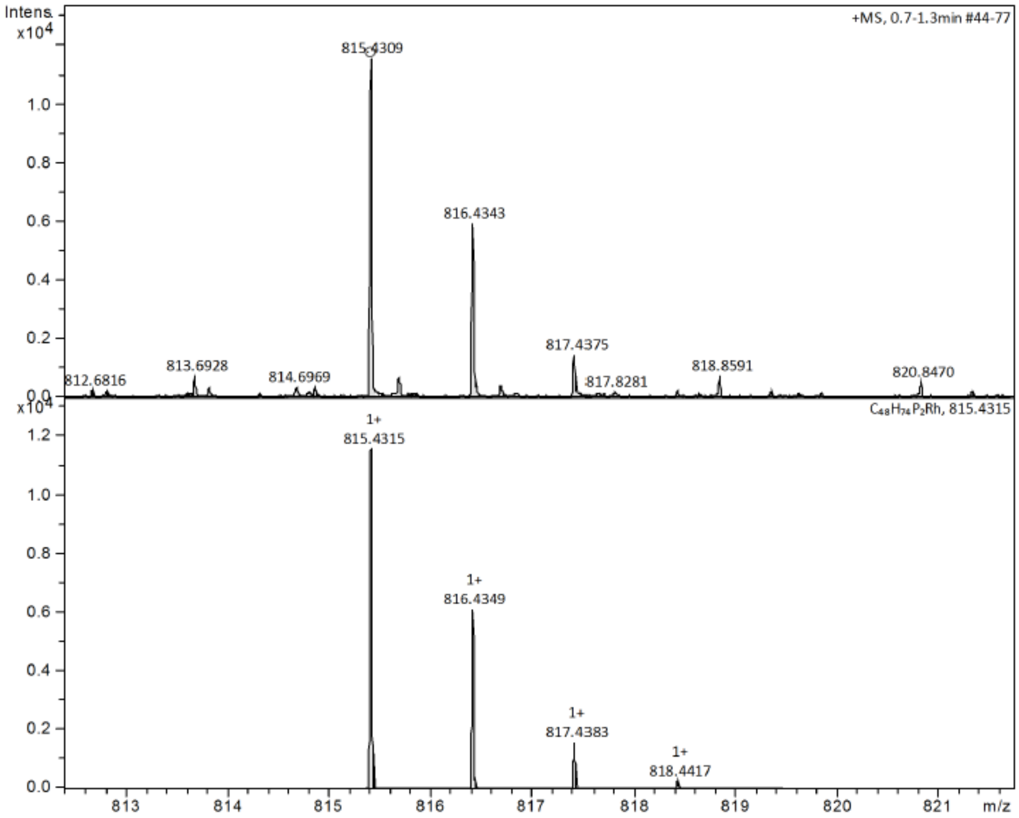

Figure S71: HR ESI-MS spectrum of **3b**

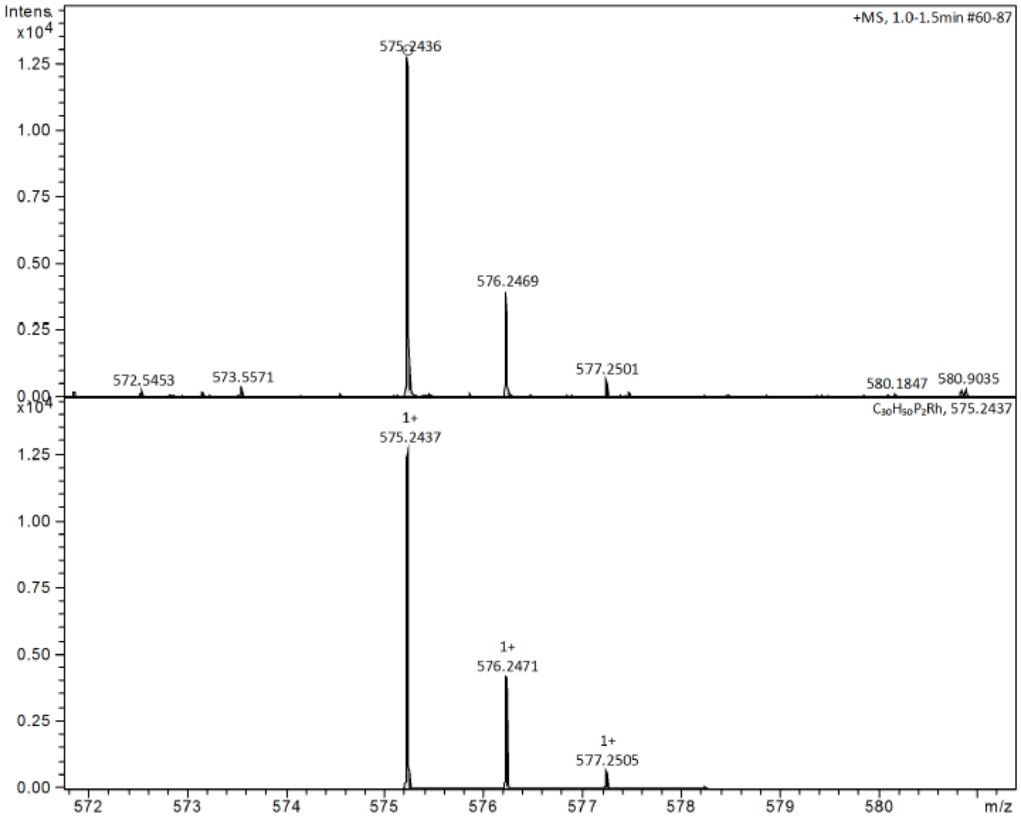

Figure S72: HR ESI-MS spectrum of **3c**

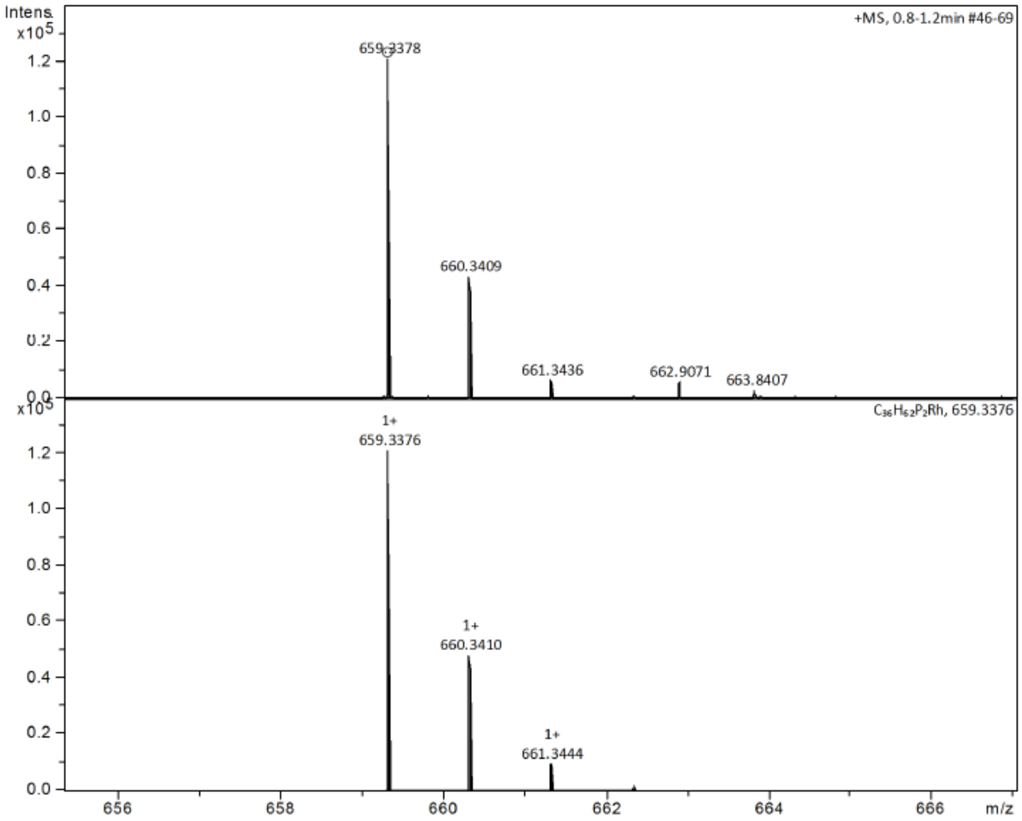

Figure S73: HR ESI-MS spectrum of **3d**

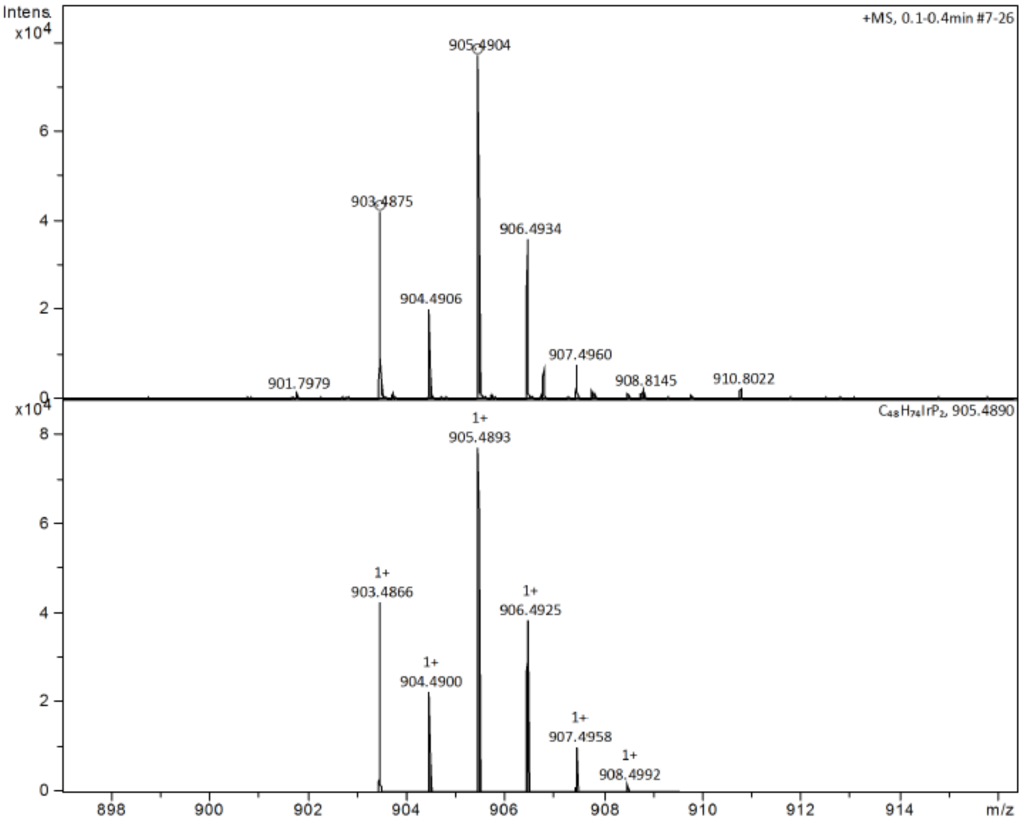

Figure S74: HR ESI-MS spectrum of **4b**

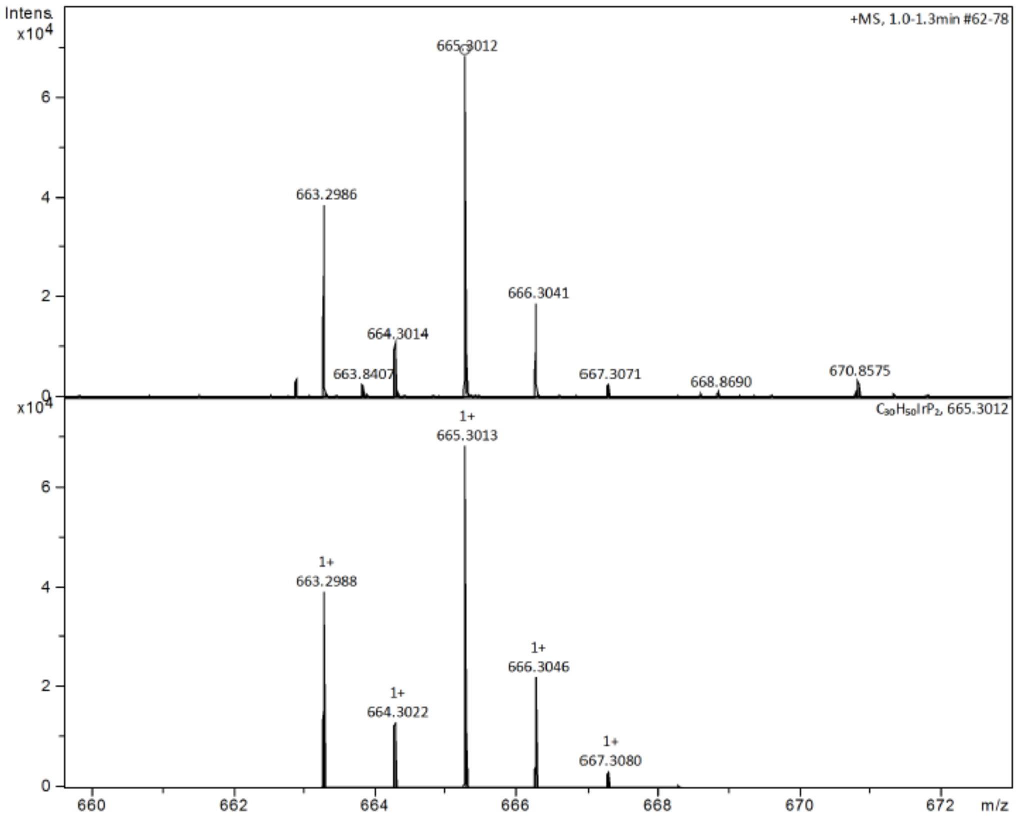

Figure S75: HR ESI-MS spectrum of **4c**

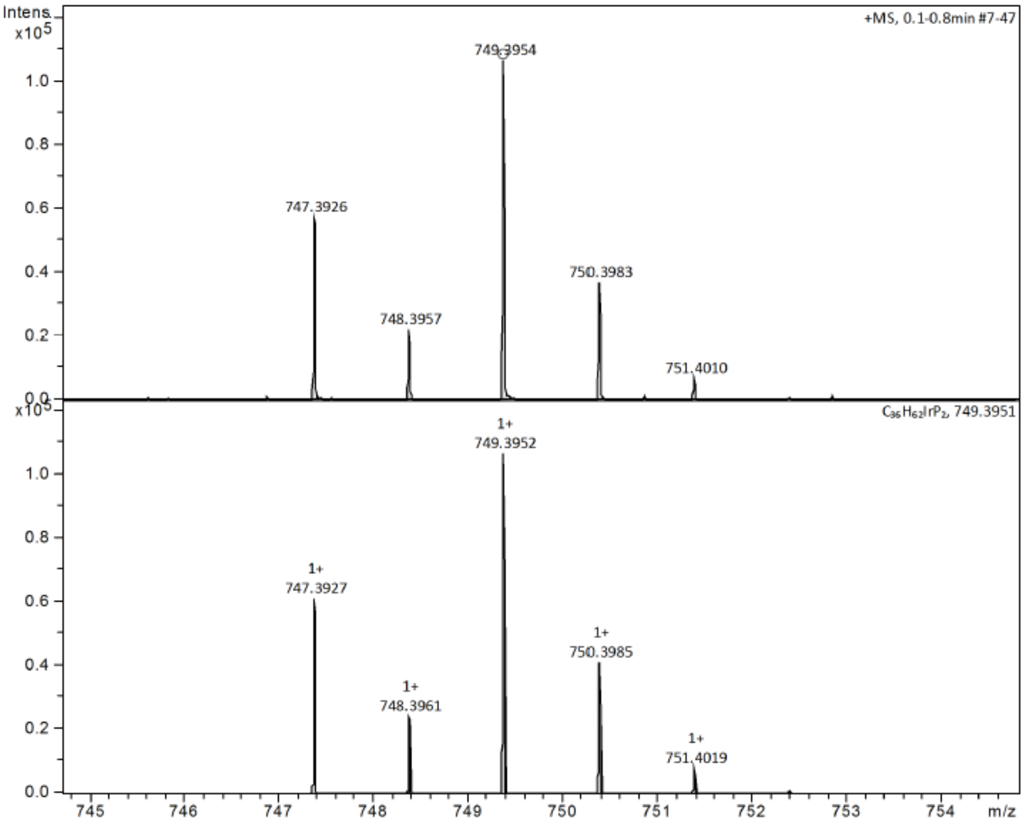

Figure S76: HR ESI-MS spectrum of **4d**

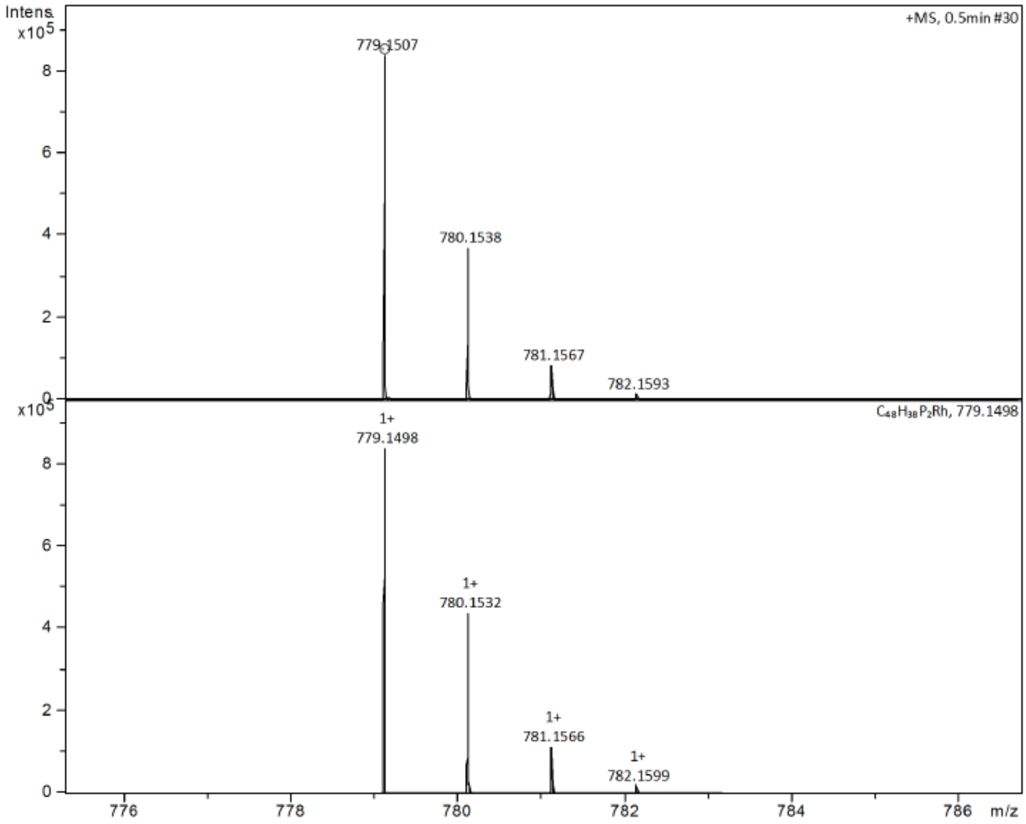

Figure S77: HR ESI-MS spectrum of **1a**

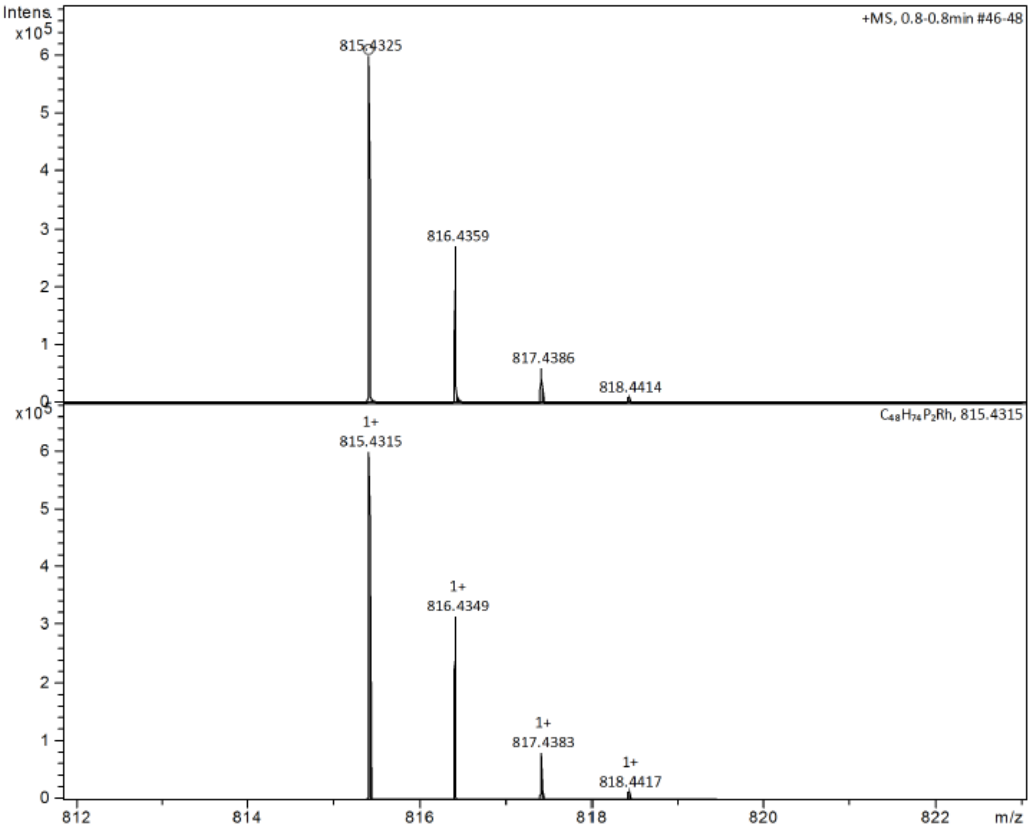

Figure S78: HR ESI-MS spectrum of **1b**

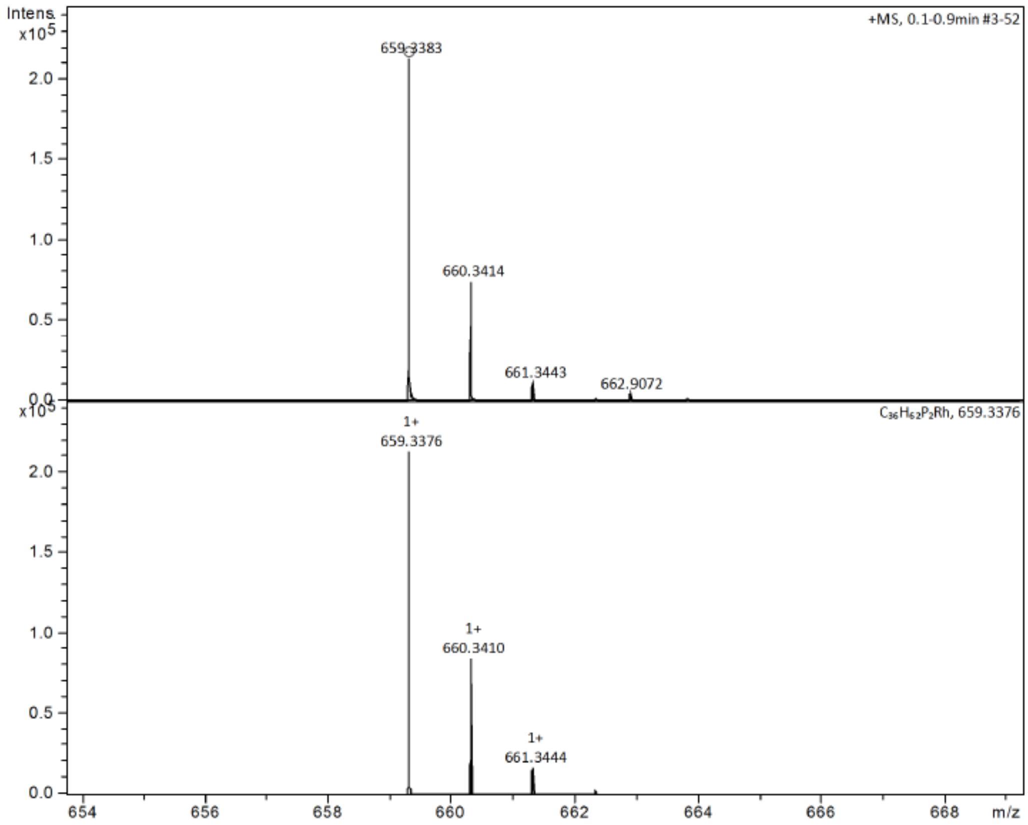

Figure S79: HR ESI-MS spectrum of **1d**

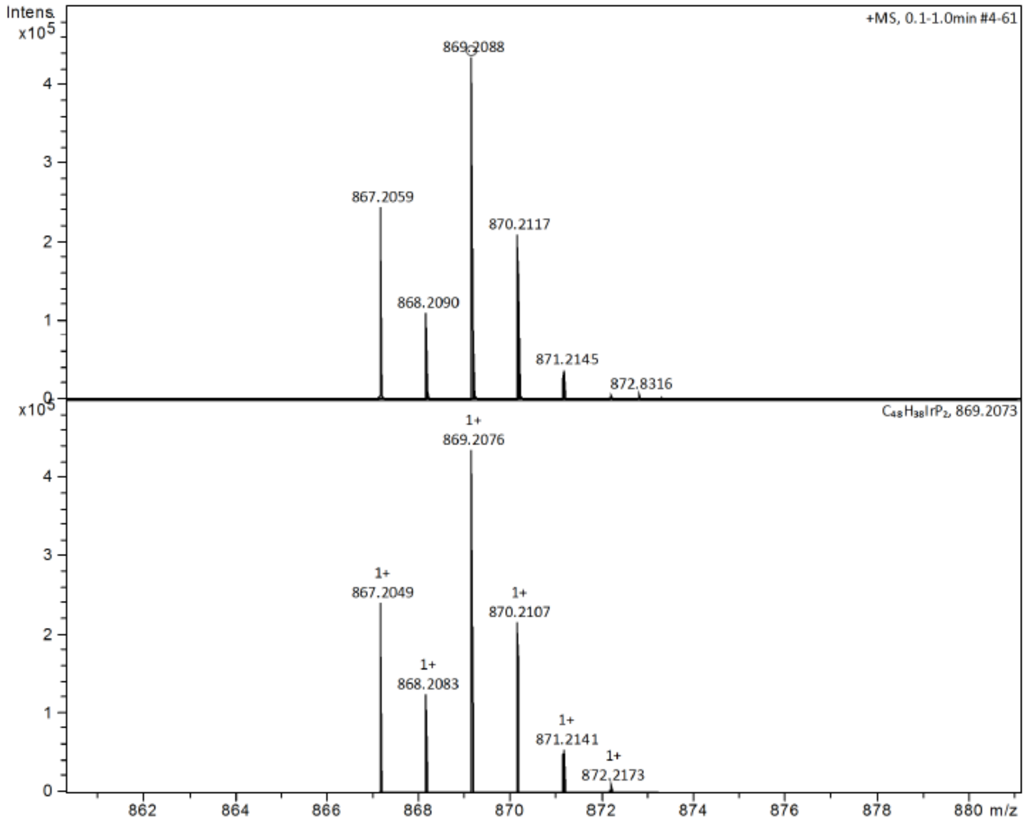

Figure S80: HR ESI-MS spectrum of **2a**

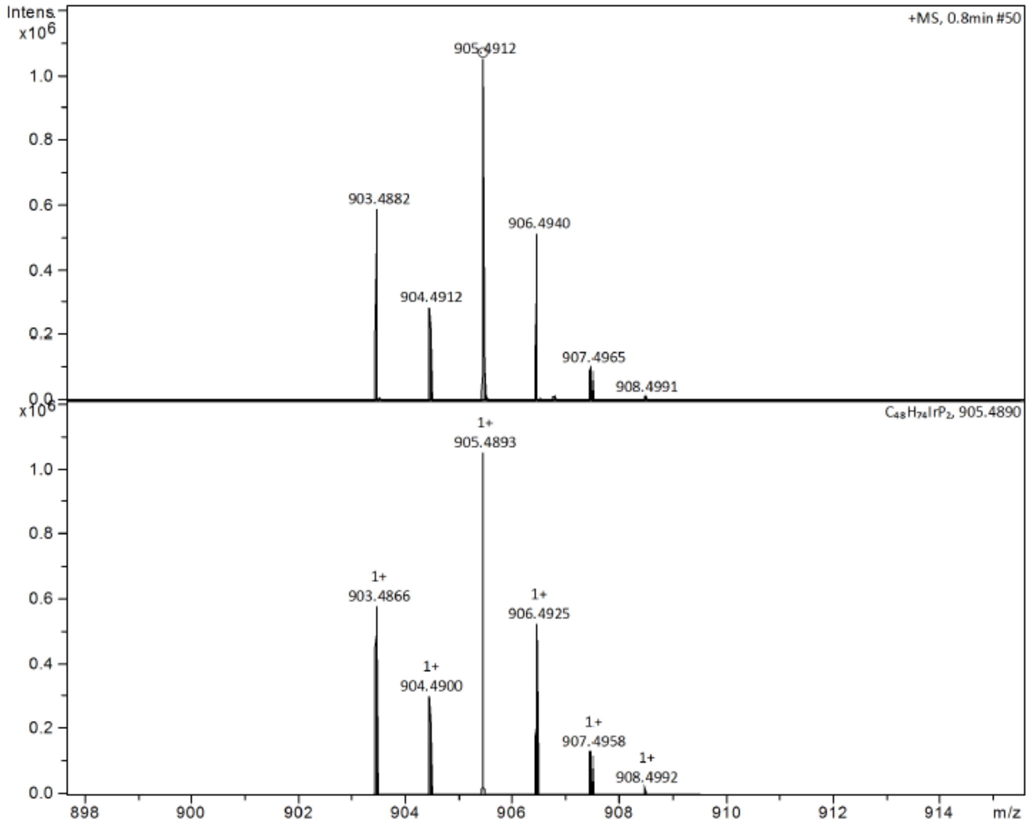

Figure S81: HR ESI-MS spectrum of **2b**

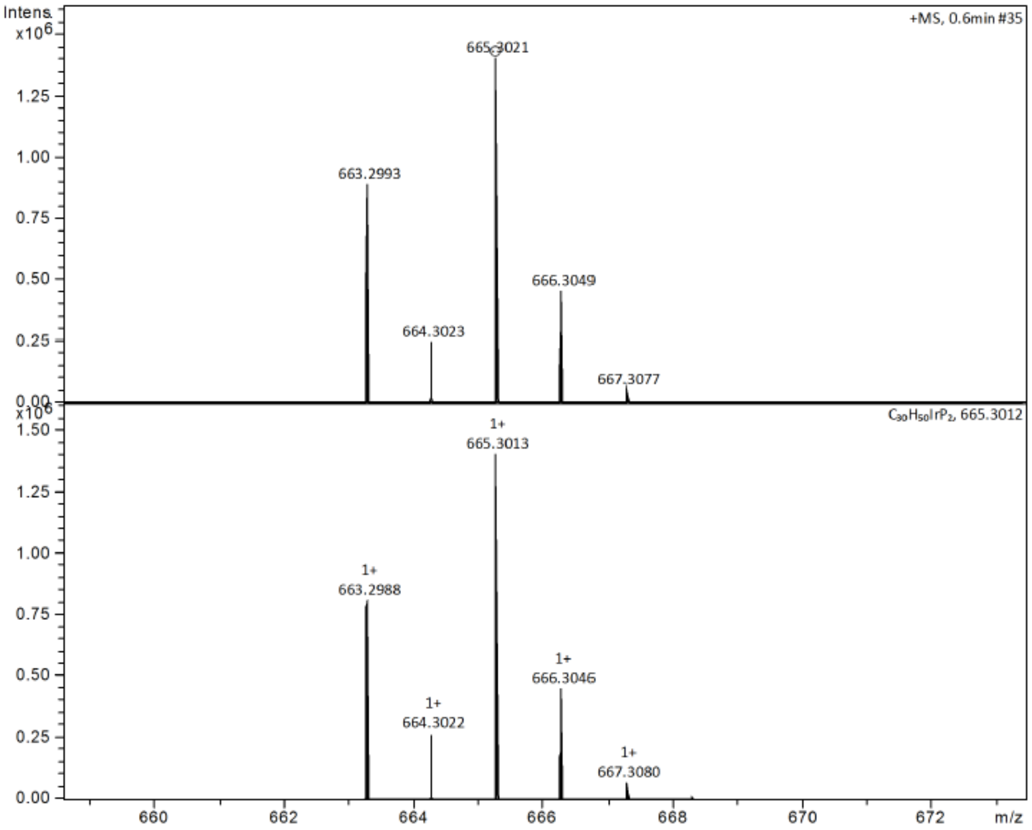

Figure S82: HR ESI-MS spectrum of **2c**

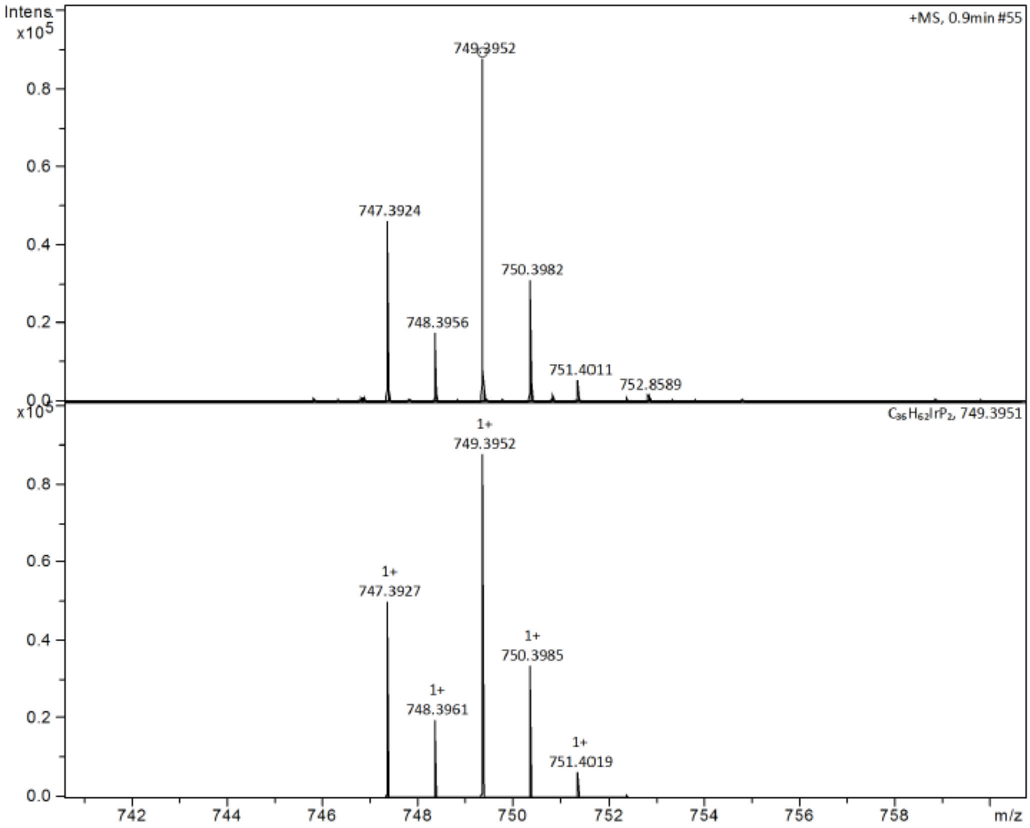

Figure S83: HR ESI-MS spectrum of **2d**

4. Crystallography

Full details about the collection, solution and refinement of crystallographic data for **1** and **2** are documented in CIFs, which have been deposited with the Cambridge Crystallographic Data Centre under CCDC 1590085-1590103 (Table S1). For ease of comparison, heavy atoms are consistently named as shown in Chart S1. Atom names of independent cations differ by 10 (Rh, Ir, P) or 100 (C). Minor disordered components of cations are indicated by the suffix A (*Z′* = 1) or names incremented by 100 (*Z′* = 2).

Table S1: CIF key

Compound	Solvent	Z′	*T* / K	CCDC number
**1a·CH_2Cl_2**	CH_2Cl_2	1	150	1590085
**1a·OH_2**	-	1	150	1590086
**1b**	CH_2Cl_2	1	25	1590087
**1b**	CH_2Cl_2	1	150	1590088
**1b**	DFB	1	25	1590089
**1b**	DFB	1	150	1590090
**1c**	-	2	150	1590091
**1d**	-	1	150	1590092
**1d***	-	1	150	1590093
**2a·CH_2Cl_2**	CH_2Cl_2	1	150	1590094
**2a·OH_2**	-	1	150	1590095
**2b**	CH_2Cl_2	1	25	1590096
**2b**	CH_2Cl_2	1	150	1590097
**2b**	DFB	1	25	1590098
**2b**	DFB	1	150	1590099
**2c**	-	2	150	1590100
**2d**	0.5 pentane	1	150	1590101
**2d***	-	1	150	1590102
**2d***	-	2	150	1590103

Chart S1: Heavy atom naming scheme for the cations of **1** and **2**.

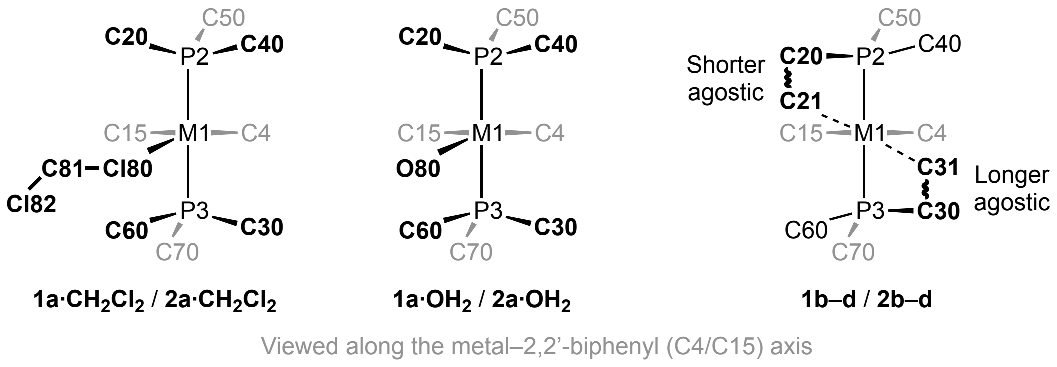

*4.1. Disorder in **1b** and **2b***

The structures of **1b**.CH_2Cl_2, **1b**.DFB, **2b**.CH_2Cl_2 and **2b**.DFB all feature a partially disordered phosphine ligand (P3) that was treated by modelling one of the substituents (C70 – C75) over two sites and restraining its geometry. There is no evidence for any meaningful disorder of the substituents directly interacting with the metal *via* agostic interactions. As a representative example, the structure of **1b**.CH_2Cl_2 determined at 150 K is depicted in Figure S84 along with a table listing the occupancy of the major disordered component in all these complexes.

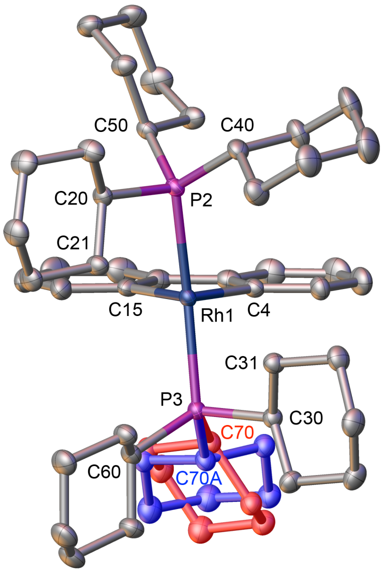

Occupancy of major disordered component (C70 – C75, red in left image)

**Compd.**	***T*** / K	**Occupancy**
**1b**.CH_2Cl_2	150	0.542(3)
**1b**.CH_2Cl_2	25	0.511(4)
**1b**.DFB	150	0.824(3)
**1b**.DFB	25	0.794(2)
**2b**.CH_2Cl_2	150	0.741(3)
**2b**.CH_2Cl_2	25	0.767(3)
**2b**.DFB	150	0.938(4)
**2b**.DFB	25	0.950(3)

Figure S84: Solid-state structure of **1b**.CH_2Cl_2 (150 K, left). Thermal ellipsoids at the 50% probability level; anion, CH_2Cl_2 solvent molecule, and H atoms omitted for clarity; major disordered component in red, minor in blue. Disorder parameters for **1** and **2** (right).

4.2. *Disorder in **1c** and **2c***

The structures of **1c** and **2c** feature two independent complexes (*Z′* = 2; Figure S85 and S86). One of the cations (Rh11/Ir11) in each solution contains two heavily disordered phosphine ligands that were treated by modelling all the substituents over two sites, with a common free variable for the occupancy (Rh11, 0.669(3); Ir11, 0.767(6)), and restraining their geometry. Whilst the presence of two agostic interactions of significantly different magnitude is evident in both components of the model, the extent of the disorder and employment of geometry restraints precludes any meaningful analysis of the associated metrics, particularly those associated with position of P12/P13. The nature of the disorder is similar in the two structures, differing only by the orientation of the C270 – C272 substituent.

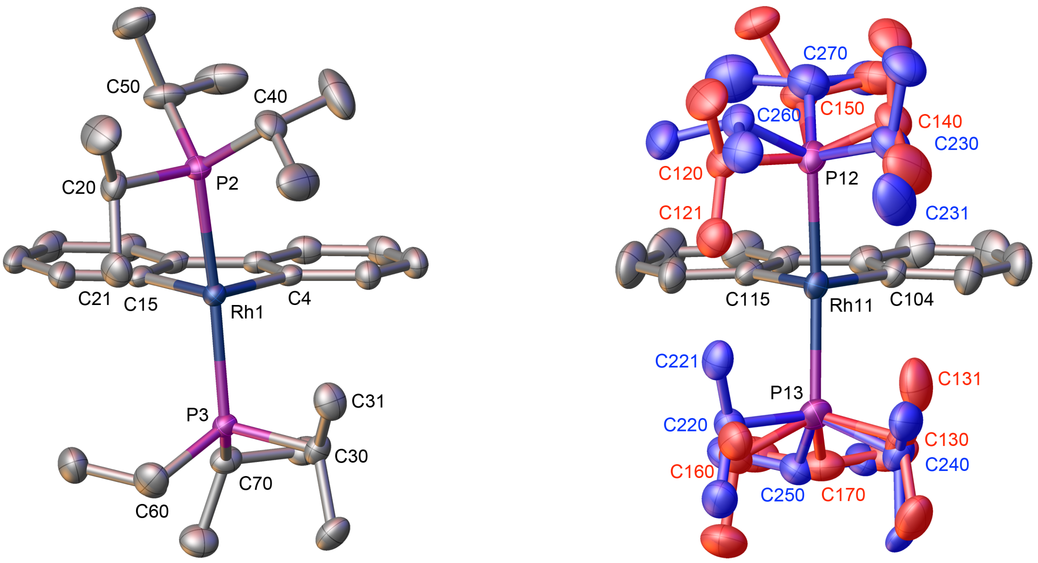

Figure S85: Two independent cations from the solid-state structure of **1c**. Thermal ellipsoids at the 50% probability level; anion and H atoms omitted for clarity; major disordered component in red, minor in blue.

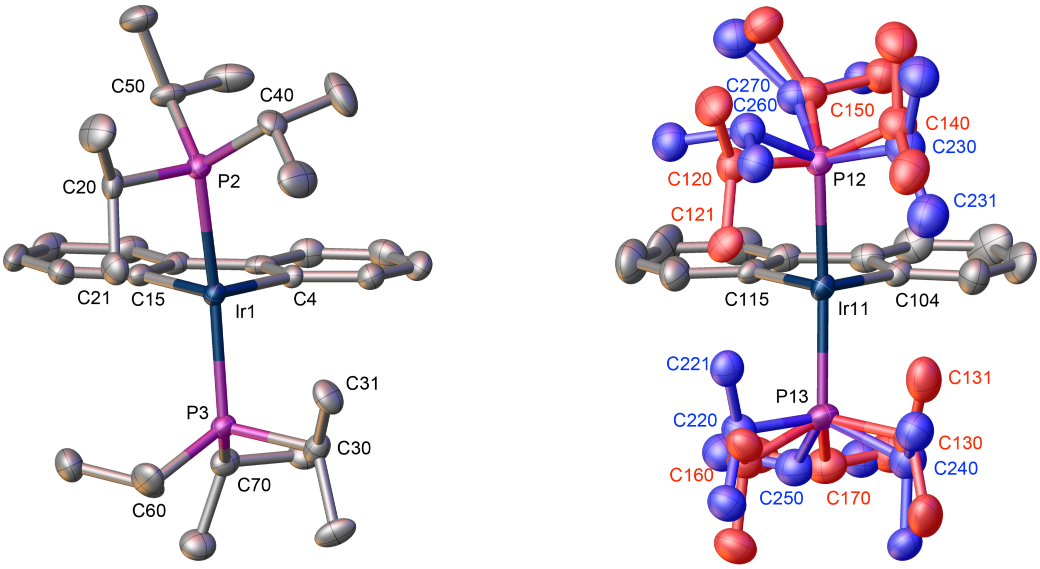

Figure S86: Two independent cations from the solid-state structure of **2c**. Thermal ellipsoids at the 50% probability level; anion and H atoms omitted for clarity; major disordered component in red, minor in blue.

*4.3. Disorder in **1d** and **2d**, and differences between the unit cells of **1d/2d** and **1d*/2d****

The structure of **1d** features a heavily disordered phosphine ligand (P2) that was treated by modelling all the substituents over two sites, with a common free variable for the occupancy (0.649(4)), and restraining their geometry; including the Rh1-C21 and Rh1-C21A contacts to equivalent length (2.954(3) Å) – see Figure S87. Given the nature of this disorder and the availability of data for non-disordered **1d***, analysis of agostic interactions in the solid-state has been focused on the metrics associated with **1d***. We do, however, note that there are some discrepancies between the metrics associated with the agostic interactions in these structures. For instance, **1d** appears to show slightly elongated values for the Rh1-C21/C21A and Rh1-C31 distances compared to **1d***: 2.954(3) *vs.* 2.863(5) Å and 3.015(3) *vs.* 2.979(4) Å. The nature of the disorder and employment of restraints in the disorder model, however, preclude a reliable conclusion to be drawn. It is also noteworthy that the substituents of the disordered phosphine ligand (P2) in **1d** show different conformations to that observed **1d***, despite the non-disordered phosphine ligand (P3) showing an essentially equivalent geometry.

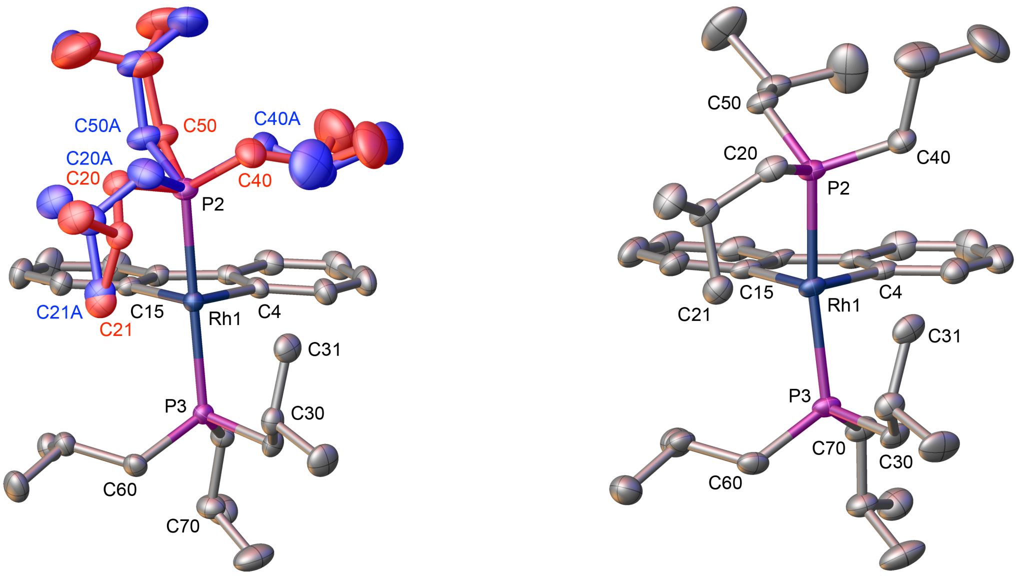

Figure S87: Solid-state structures of **1d** (left) and **1d*** (right). Thermal ellipsoids at the 50% probability level; anion and H atoms omitted for clarity; major disordered component in red, minor in blue for **1d**.

The structure of **2d** features two heavily disordered phosphine ligands that were treated by splitting either all or two of the substituents equally over two sites and restraining their geometry (Figure S88). The component with the shortest principle agostic interaction (Ir1-C21) was named as if it was the major component. Given the nature of this disorder and the availability of data for non-disordered **2d*** (Figure S89), analysis of agostic interactions in the solid-state has been focused on the metrics associated with **2d***. We do, however, note that there are some discrepancies between metrics associated with the agostic interactions in the disordered components of **2d**, and those observed in **2d***. For instance, the values for the Ir1-C21/C21A distance in **2d** (2.766(5), 2.881(6) Å) are skewed away from those determined in **2d*** (2.781(7), 2.790(7) Å). Ultimately, however, the nature and extent of this disorder (and employment of

geometry restraints) hinders reliable analysis of the associated metrics in **2d**. There are some broad similarities between the conformations of the phosphine ligands observed in disordered **2d** (e.g. viewed as in Figure S88 – right) compared to those observed in **2d*** (e.g. viewed as in Figure S89 – top left).

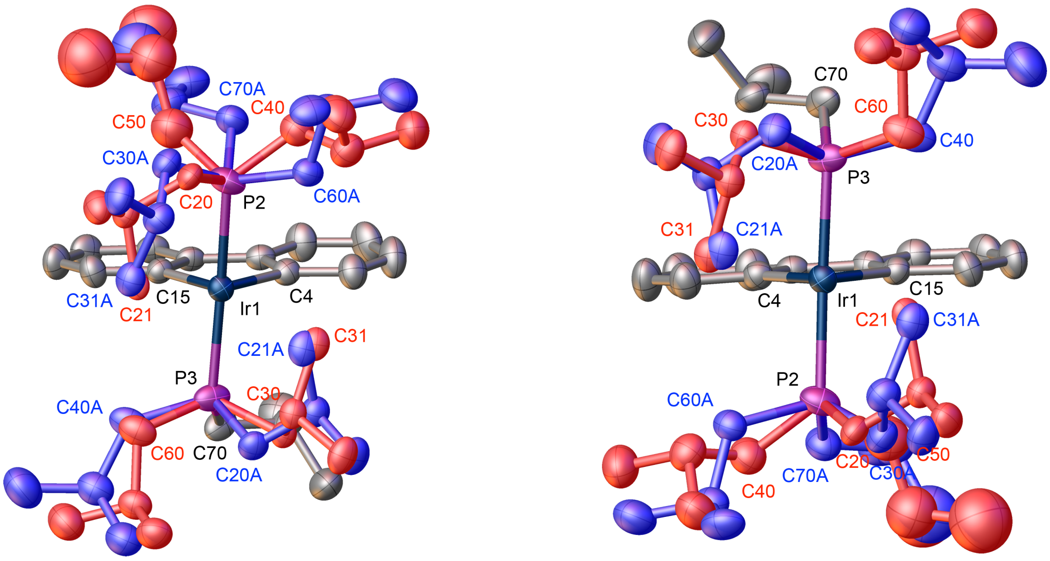

Figure S88: Solid-state structure of **2d** – viewed in two different orientations. Thermal ellipsoids at the 50% probability level; pentane solvent molecule, anion and H atoms omitted for clarity; shorter principle agostic disordered components in red, longer principle agostic disordered components in blue.

Curiously despite very similar experimental conditions, it appears **1d** crystallises without solvent, whilst **2d** crystallises with half a molecule of pentane solvent per complex (only a limited range of single crystals samples for each were screened). Moreover, crystallographic differences are also observed between **1d*** and **2d***, with the latter associated with unit cell of approximately double volume (6601.6(2) *vs.* 3287.91(17) Å^3) and two independent cations (Figure S88). The data for **2d*** shows pseudo translation and the structure can be alternatively solved with a smaller unit cell, very similar to that of **1d*** (P-1, a = 10.8847(3) Å, b = 17.6478(3) Å, c = 17.9164(3) Å, α = 89.353(2)°, β = 80.948(2)°, γ = 76.535(2)°; *cf.* P-1, a = 10.8075(3) Å, b = 17.4528(5) Å, c = 18.0496(4) Å, α = 89.372(2)°, β = 81.615(2)°, γ = 77.528(3)°), but bearing a heavily disordered cation that was partially treated by splitting two of the phosphine substituents (C40 – C43 and C50 – C53) equally over two sites and restraining their geometry (Figure S89). The component with the same conformation as the Ir1 cation of the larger unit cell (and **1d***) was named as if it was the major component. Combined these observations point to subtle divergences in crystal packing between the rhodium and iridium complexes of P*i*Bu$_3$, for both $[BAr^{F}_{4}]^{-}$ and $[Al\{OC(CF_3)_3\}_4]^{-}$ counter anions, with the heavier congener showing greater propensity for positional disorder of the phosphine substituents. Given the similarly of the crystallography associated with **1a** – **1c** / **2a** – **2c**, the differences seen between **1d**/**2d** and **1d***/**2d*** are presumably only possible due to the flexible nature of the *i*Bu substituents.

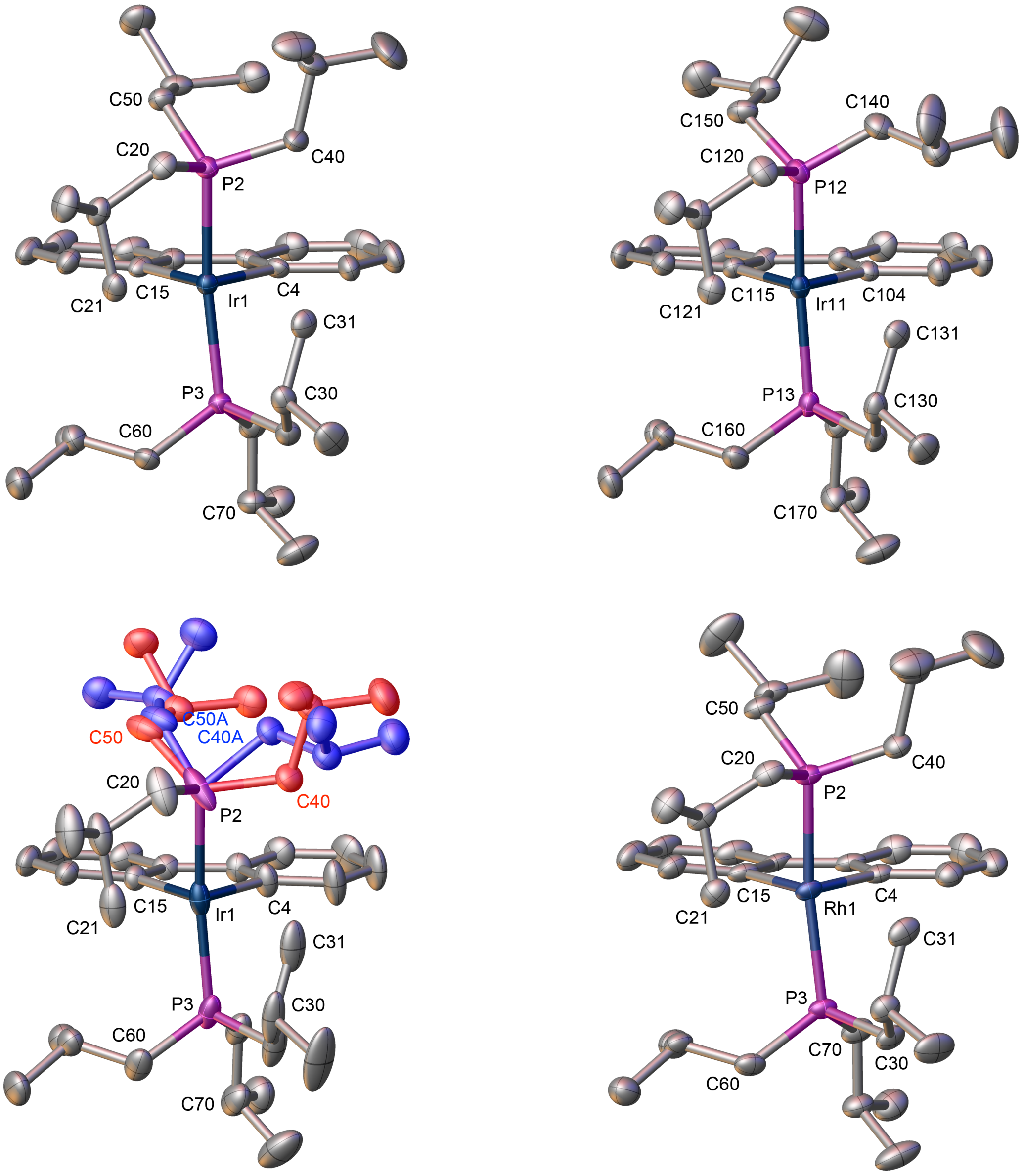

Figure S89: Solid-state structures of **2d*** (top, two independent cations; bottom left, obtained using a smaller unit cell) and **1d*** (bottom right). Thermal ellipsoids at the 50% probability level; anion and H atoms omitted for clarity; disordered component with closet conformational agreement to the Ir1 cation of the larger unit cell (and **1d***) in red, disordered component with closet conformational agreement to the Ir11 cation of the larger unit cell in blue.

Despite good agreement of the metrics associated with the adoption of agostic interactions between the two independent cations in **2d*** (see Table S2 and S3, below), for simplicity and consistency the discussion is focused on the cation bearing closest conformational similarly to that of **1d*** (*viz.* Ir1, Figure S89).

4.4. *More extensive metrics associated with the solid-state structures of **1** and **2**.*

Table S2: Selected bond lengths in solid-state structures of **1** and **2** (Å).

Compd.	#[a]	*T* / K	M1-P2	M1-P3	M1-C4	M1-C15	M1-C21	M1-C31
**1a·CH_2Cl_2**	-	150	2.3648(7)	2.3437(7)	2.007(3)	2.000(3)	-	3.271(4)
**2a·CH_2Cl_2**	-	150	2.3513(9)	2.3421(9)	2.020(4)	2.012(4)	-	3.349(5)
**1b**.CH_2Cl_2	-	150	2.3755(7)	2.3636(7)	1.996(3)	1.994(3)	2.877(3)	2.899(3)
**1b**.CH_2Cl_2	-	25	2.3754(9)	2.3618(9)	1.999(4)	1.999(3)	2.854(4)	2.891(3)
**1b**.DFB	-	150	2.3758(6)	2.3617(5)	1.999(2)	1.993(2)	2.864(2)	2.877(2)
**1b**.DFB	-	25	2.3765(5)	2.3607(5)	2.0006(18)	1.9990(18)	2.8605(18)	2.8729(18)
**2b**.CH_2Cl_2	-	150	2.3614(7)	2.3608(7)	2.016(3)	2.010(3)	2.857(3)	2.875(3)
**2b**.CH_2Cl_2	-	25	2.3602(7)	2.3579(7)	2.014(3)	2.012(3)	2.837(3)	2.869(3)
**2b**.DFB	-	150	2.3651(6)	2.3581(6)	2.010(2)	2.016(2)	2.842(3)	2.856(2)
**2b**.DFB	-	25	2.3665(7)	2.3580(6)	2.015(3)	2.025(3)	2.844(3)	2.859(3)
**1c** (Rh1)	-	150	2.3593(7)	2.3542(7)	1.989(2)	1.995(2)	2.836(3)	3.185(3)
**1c** (Rh11)	M	150	2.3615(8)	2.3525(8)	1.983(3)	1.977(3)	2.895(5)	3.160(8)
**1c** (Rh11)	m	150	-	-	-	-	2.937(10)	3.490(18)
**2c** (Ir1)	-	150	2.352(2)	2.347(2)	2.021(7)	2.015(8)	2.810(8)	3.115(9)
**2c** (Ir11)	M	150	2.355(2)	2.344(2)	2.005(8)	1.996(8)	2.848(11)	2.994(14)
**2c** (Ir11)	m	150	-	-	-	-	2.96(4)	3.25(5)
**1d**	M	150	2.3426(7)	2.3441(6)	2.005(2)	1.997(2)	2.954(3)[b]	3.015(3)
**1d**	m	150	-	-	-	-	2.954(3)[b]	-
**1d***	-	150	2.3301(10)	2.3545(10)	1.992(4)	2.003(4)	2.863(5)	2.979(4)
**2d**.C_5H_{12}	S	150	2.3279(9)	2.3285(9)	2.018(3)	2.022(3)	2.766(5)	2.948(6)
**2d**. C_5H_{12}	L	150	-	-	-	-	2.881(6)	2.959(6)
**2d*** (Ir1)[c]	-	150	2.3301(15)	2.3501(15)	2.017(6)	2.024(6)	2.781(7)	2.956(6)
**2d*** (Ir11)	-	150	2.3476(16)	2.3564(17)	2.014(6)	2.018(6)	2.790(7)	2.975(6)

[a] Disordered component: M = major, m = minor; S = featuring shorter principle agostic bond, L = featuring longer principle agostic bond. [b] Restrained to equivalent length. [c] Equivalent conformation to that of **1d***.

Table S3: Selected bond angles in the solid-state structures of **1** and **2** (°).

Compd.	#[a]	*T* / K	P2-M1-P3	C4-M1-C15	P2<pln[b]	C4-M1-P2	C15-M1-P2	M1-P2-C20	M1-P2-C40	M1-P2-C50	P3<pln[b]	C15-M1-P3	C4-M1-P3	M1-P3-C30	M1-P3-C60	M1-P2-C70
**1a·CH_2Cl_2**	-	150	172.05(2)	81.75(12)	3.86(6)	86.27(8)	90.32(8)	114.58(9)	111.13(10)	115.94(9)	4.24(7)	91.49(8)	86.32(8)	104.26(10)	114.77(9)	119.53(9)
**2a·CH_2Cl_2**	-	150	172.63(3)	81.34(16)	3.27(9)	87.23(10)	90.73(10)	115.19(12)	109.78(13)	115.60(12)	4.48(10)	92.45(10)	86.68(10)	106.40(13)	115.04(12)	117.89(12)
**1b**.CH_2Cl_2	M	150	170.82(2)	82.49(11)	8.04(6)	97.89(8)	90.06(8)	97.05(9)	117.33(9)	120.50(9)	5.97(6)	95.89(8)	89.83(8)	96.77(8)	113.42(9)	115.68(17)
**1b**.CH_2Cl_2	M	25	170.80(3)	82.60(15)	8.07(8)	97.81(10)	90.04(10)	96.82(11)	117.48(11)	120.70(11)	6.00(8)	95.91(10)	89.93(10)	96.61(11)	113.34(12)	115.5(2)
**1b**.DFB	M	150	170.68(2)	81.96(9)	9.74(5)	98.98(6)	91.17(6)	96.00(7)	117.74(7)	120.76(7)	4.53(6)	94.82(6)	88.94(6)	96.33(7)	113.91(7)	117.67(10)
**1b**.DFB	M	25	170.419(17)	81.97(8)	10.31(4)	99.42(5)	91.43(5)	95.94(6)	118.01(6)	120.90(6)	4.41(5)	94.61(5)	88.80(5)	96.23(6)	113.85(6)	117.79(8)
**2b**.CH_2Cl_2	M	150	169.91(2)	82.29(11)	8.88(6)	98.57(8)	90.22(8)	97.22(9)	117.91(10)	119.80(9)	6.41(7)	96.59(8)	89.74(8)	96.66(9)	114.40(9)	115.53(13)
**2b**.CH_2Cl_2	M	25	169.92(2)	82.35(11)	8.88(6)	98.46(8)	90.17(8)	96.99(8)	117.96(9)	120.01(9)	6.37(6)	96.64(8)	89.84(8)	96.64(9)	114.34(9)	115.64(12)
**2b**.DFB	M	150	169.87(2)	81.64(10)	10.55(5)	99.58(7)	91.27(7)	96.15(8)	118.13(8)	120.00(8)	4.89(6)	95.33(7)	89.00(7)	96.23(8)	114.53(8)	117.47(19)
**2b**.DFB	M	25	169.71(3)	81.67(11)	10.93(5)	99.81(7)	91.69(7)	96.37(8)	118.33(8)	119.92(9)	4.52(7)	94.86(7)	89.01(7)	96.12(8)	114.28(8)	117.66(10)
**1c** (Rh1)	-	150	172.64(2)	82.34(11)	9.00(6)	98.06(7)	89.38(7)	96.35(9)	118.17(10)	119.23(10)	4.16(7)	94.06(7)	88.86(7)	102.71(9)	114.42(9)	119.72(9)
**1c** (Rh11)	M	150	172.90(3)	82.78(12)	2.20(6)	92.90(8)	91.10(8)	94.63(16)	114.7(2)	115.68(19)	5.11(5)	92.71(8)	93.52(8)	100.9(2)	113.55(17)	124.5(3)
**1c** (Rh11)	m	150	-	-	-	-	-	93.6(3)	110.2(4)	127.0(5)	-	-	-	102.8(4)	118.9(3)	126.5(5)
**2c** (Ir1)	-	150	171.10(7)	81.9(3)	10.35(17)	99.2(2)	90.0(2)	96.3(3)	119.1(3)	117.7(3)	4.4(2)	94.5(2)	89.0(2)	101.1(3)	115.0(3)	119.3(3)
**2c** (Ir11)	M	150	171.58(9)	82.4(3)	3.00(18)	93.5(2)	90.8(2)	95.6(4)	116.2(4)	117.4(6)	5.96(15)	94.2(2)	93.9(2)	98.5(4)	114.0(4)	123.5(5)
**2c** (Ir11)	m	150	-	-	-	-	-	94.8(10)	112.5(11)	124.6(14)	-	-	-	102.9(12)	116.8(11)	116(2)
**1d**	M	150	179.29(3)	82.06(10)	4.63(6)	93.49(7)	87.03(7)	104.53(15)	116.73(17)	116.1(4)	5.32(6)	93.51(7)	86.13(7)	106.65(9)	115.50(9)	116.75(8)
**1d**	m	150	-	-	-	-	-	112.2(3)	115.2(3)	116.9(7)	-	-	-	-	-	-
**1d***	-	150	171.77(4)	82.03(18)	5.28(7)	92.69(11)	92.49(11)	105.58(15)	108.99(14)	123.91(15)	6.61(11)	95.57(11)	86.79(11)	104.93(14)	116.66(15)	116.11(15)
**2d**.C_5H_{12}	S	150	175.31(3)	81.80(13)	4.54(8)	89.57(9)	93.82(10)	105.48(18)	127.4(2)	112.99(16)	5.26(9)	88.76(9)	94.67(9)	104.66(18)	111.84(16)	118.82(12)
**2d**. C_5H_{12}	L	150	-	-	-	-	-	111.32(18)	108.14(16)	-	-	-	-	109.80(19)	104.96(15)	119.06(19)
**2d*** (Ir1)[c]	-	150	170.94(6)	81.7(3)	6.71(10)	93.47(17)	93.05(16)	105.4(2)	109.0(2)	123.6(2)	6.28(16)	96.00(16)	87.55(17)	106.3(2)	117.4(2)	115.0(2)
**2d*** (Ir11)	-	150	174.28(6)	81.7(2)	5.50(11)	93.69(17)	91.20(17)	105.3(2)	117.3(2)	119.2(2)	5.08(16)	94.51(17)	87.43(17)	106.9(2)	117.2(2)	115.0(2)

[a] Disordered component: M = major, m = minor; S = featuring shorter principle agostic bond, L = featuring longer principle agostic bond. [b] Angle between the M1-P2/3 vector and the normal vector of the M1-C4-C9-C10-C15 (metallacycle) least squares plane. [c] Equivalent conformation to that of **1d***.

4.5. *Structures of **1a·OH₂** and **2a·OH₂***

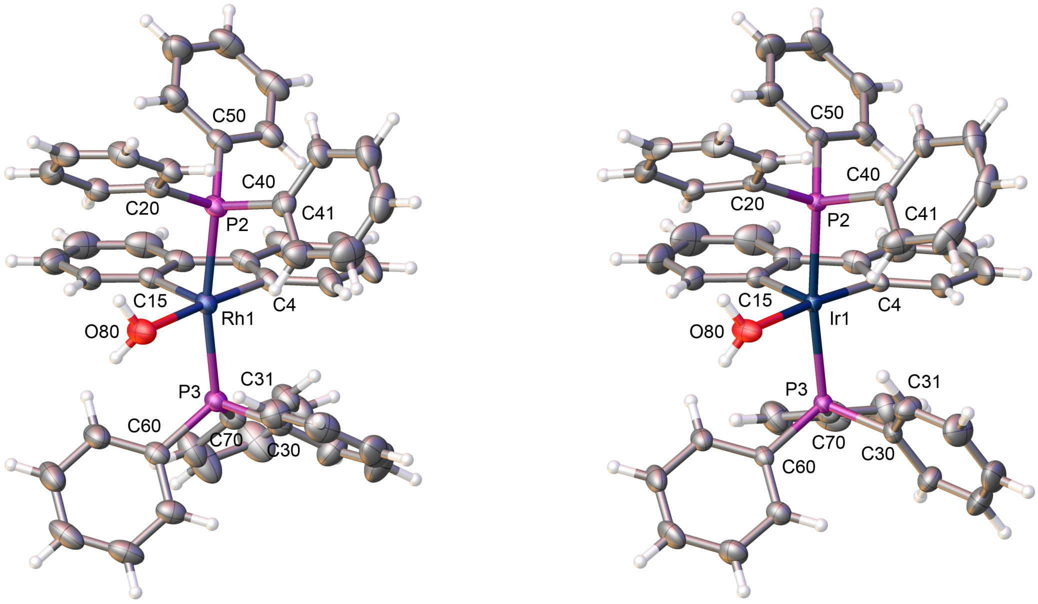

Figure S90: Solid-state structures of **1a·OH$_2$** (left) and **2a·OH$_2$** (right). Thermal ellipsoids at the 50% probability level; anion omitted for clarity. Selected bond lengths (Å) and angles (deg): **1a·OH$_2$**: Rh1-P2, 2.3400(5); Rh1-P3, 2.3338(5); Rh1-O80, 2.2441(16); Rh1-C4, 2.000(2); Rh1-C15, 1.991(2); Rh1-C31, 3.304(3); Rh1-C41, 3.505(3); O80-H31, 2.641(2); O80-H41, 3.250(2); P2-Rh1-P3, 169.34(2); C4-Rh1-C15, 81.93(10); P2<npln, 5.83(6); C15-Rh1-P2, 94.01(6); Rh1-P2-C40, 107.86(8); P3<npln, 5.33(6); C15-Rh1-P3, 93.75(6); Rh1-P3-C30, 98.85(7); O80-H31-C31, 150.2(2); O80-H41-C41, 140.4(2); **2a·OH$_2$**: Ir1-P2, 2.3329(7); Ir1-P3, 2.3225(7); Ir1-O80, 2.232(2); Ir1-C4, 2.010(3); Ir1-C15, 2.006(3); Ir1-C31, 3.330(3); Ir1-C41, 3.576(4); O80-H31, 3.108(2); O80-H41, 3.192(2); P2-Ir1-P3, 172.25(2); C4-Ir1-C15, 81.05(12); P2<npln, 2.51(6); C15-Ir1-P2, 92.53(8); Ir1-P2-C40, 108.41(10); P3<npln, 6.47(8); C15-Ir1-P3, 93.60(8); Ir1-P3-C30, 105.81(10); O80-H31-C31, 139.2(2); O80-H41-C41, 141.5(2).

5. Computational analysis

5.1. Selected NBO orbital overlaps associated with perturbation analysis

Table S4: Selected NBO orbital overlaps (isovalue 0.04) for **1a'**

**Agostic**	$\sigma_{CH} \rightarrow \sigma^*_{MC}$		$\sigma_{MC} \rightarrow \sigma^*_{CH}$	
	trans	*cis*	*trans*	*cis*
major	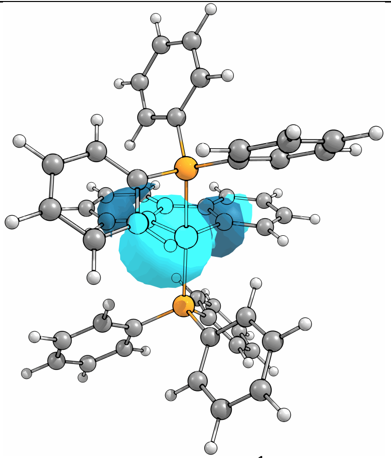<br>0.84 kcal·mol^{-1}	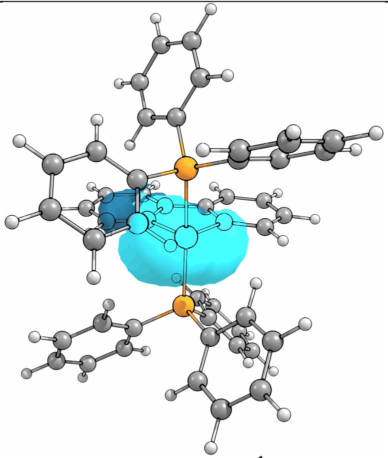<br>0.23 kcal·mol^{-1}	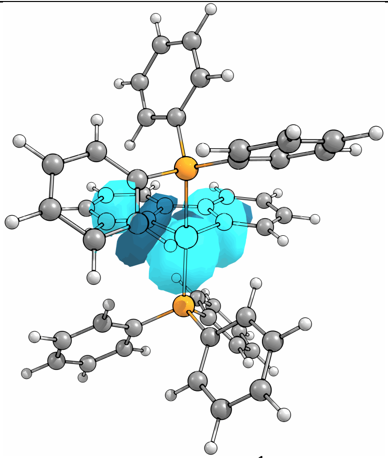<br>0.25 kcal·mol^{-1}	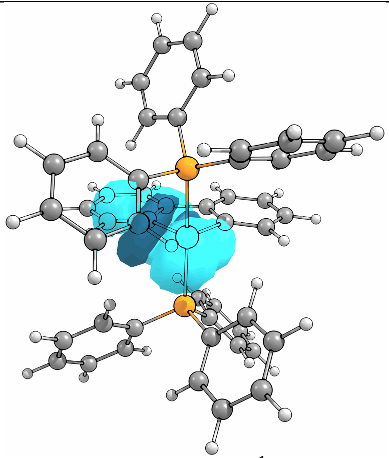<br>0.25 kcal·mol^{-1}
minor	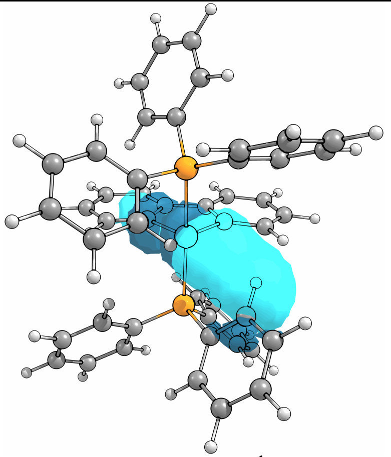<br>1.16 kcal·mol^{-1}	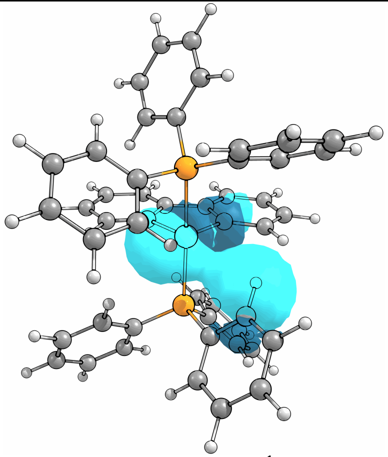<br>< 0.05 kcal·mol^{-1}	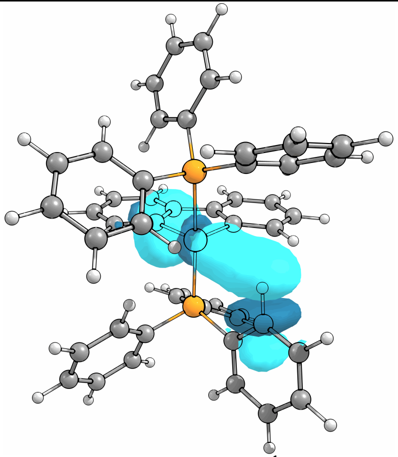<br>< 0.05 kcal·mol^{-1}	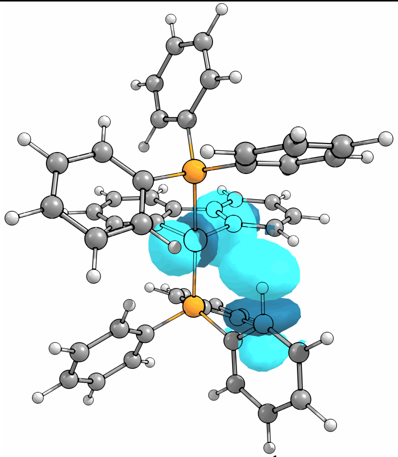<br>< 0.05 kcal·mol^{-1}

Table S5: Selected NBO orbital overlaps (isovalue 0.04) for **2a'**

**Agostic**	$\sigma_{CH} \rightarrow \sigma^*_{MC}$		$\sigma_{MC} \rightarrow \sigma^*_{CH}$	
	trans	*cis*	*trans*	*cis*
major	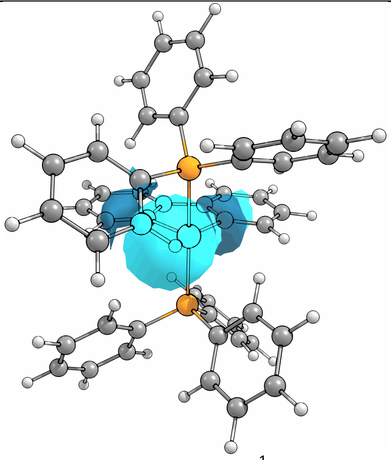 1.20 kcal·mol^{-1}	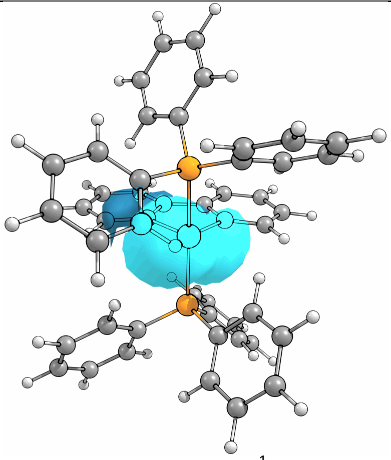 0.45 kcal·mol^{-1}	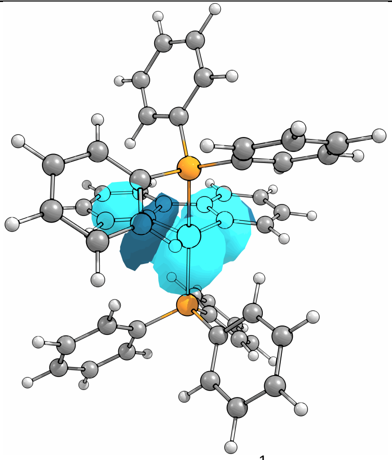 0.33 kcal·mol^{-1}	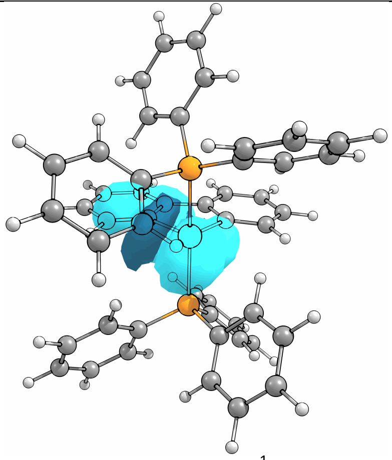 0.31 kcal·mol^{-1}
minor	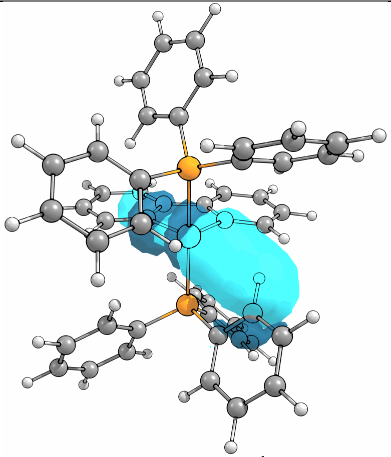 1.64 kcal·mol^{-1}	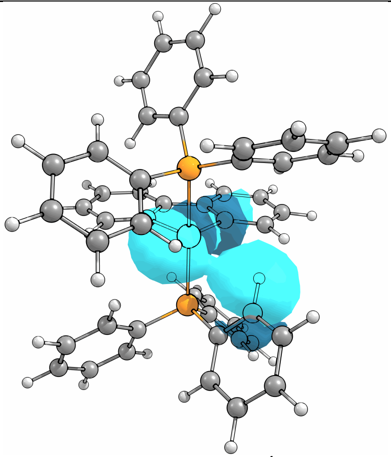 < 0.05 kcal·mol^{-1}	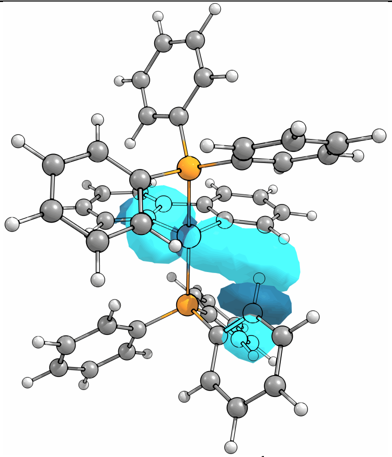 0.71 kcal·mol^{-1}	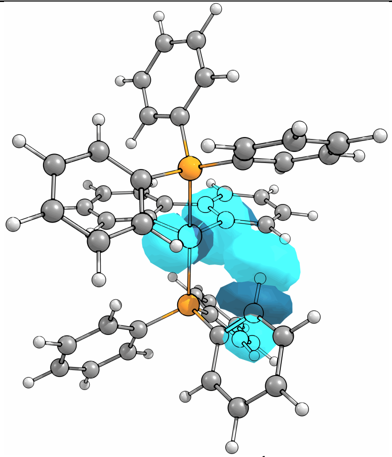 1.01 kcal·mol^{-1}

Table S6: Selected NBO orbital overlaps (isovalue 0.04) for **1b’**

**Agostic**	$\sigma_{CH} \rightarrow \sigma^*_{MC}$ *trans*	*cis*	$\sigma_{MC} \rightarrow \sigma^*_{CH}$ *trans*	*cis*
major	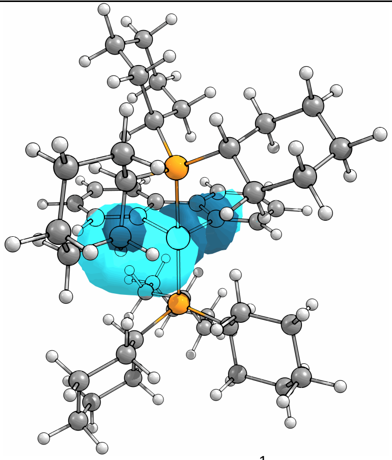 8.10 kcal·mol^{-1}	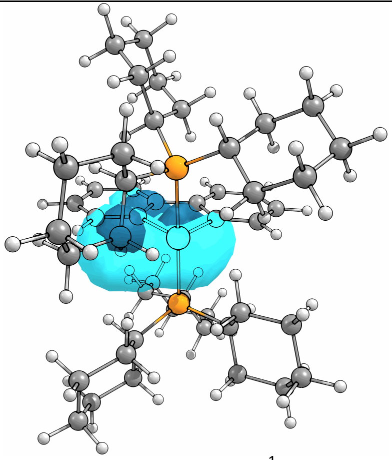 < 0.05 kcal·mol^{-1}	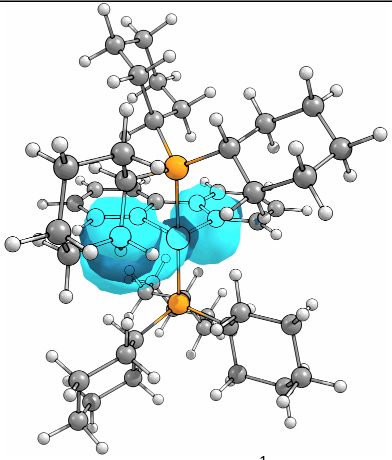 1.53 kcal·mol^{-1}	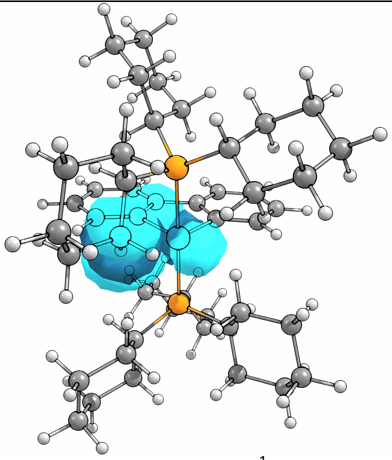 2.08 kcal·mol^{-1}
minor	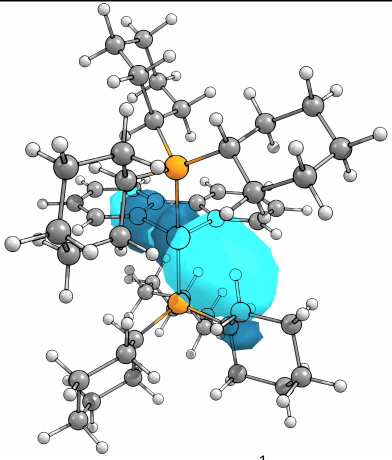 6.12 kcal·mol^{-1}	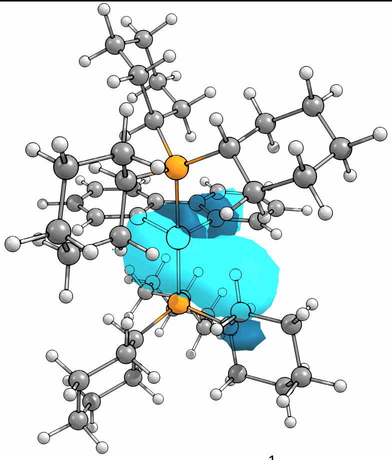 < 0.05 kcal·mol^{-1}	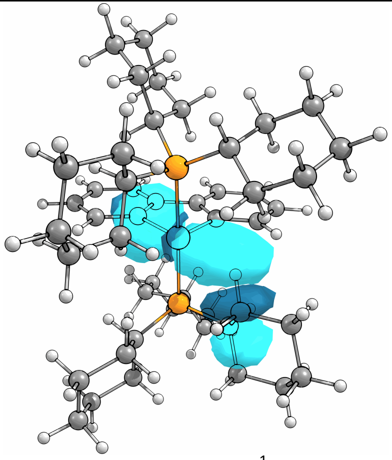 1.65 kcal·mol^{-1}	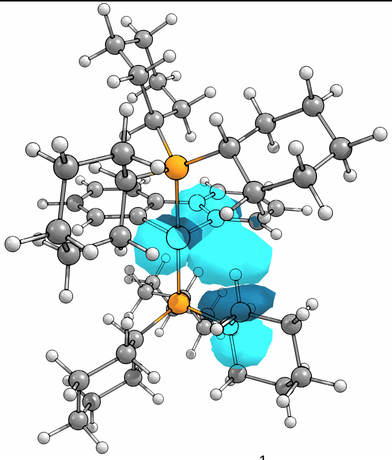 2.33 kcal·mol^{-1}

Table S7: Selected NBO orbital overlaps (isovalue 0.04) for **2b’**

**Agostic**	**$\sigma_{CH} \rightarrow \sigma^*_{MC}$**		**$\sigma_{MC} \rightarrow \sigma^*_{CH}$**	
	trans	*cis*	*trans*	*cis*
major	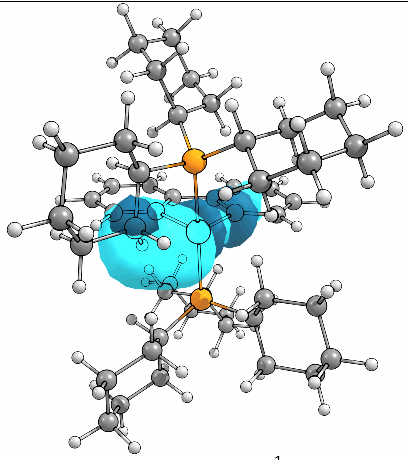 10.65 kcal·mol^{-1}	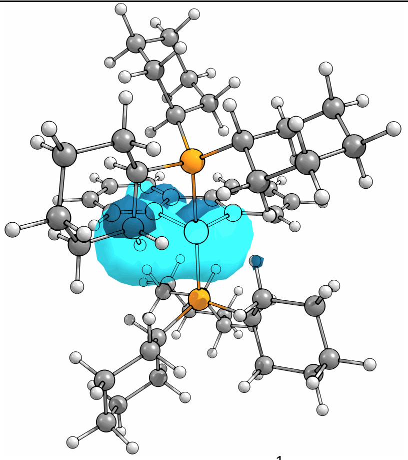 < 0.05 kcal·mol^{-1}	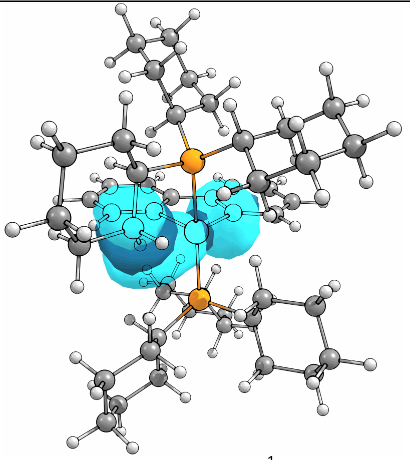 2.82 kcal·mol^{-1}	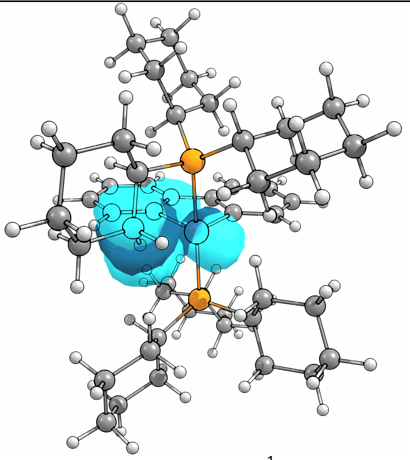 3.27 kcal·mol^{-1}
minor	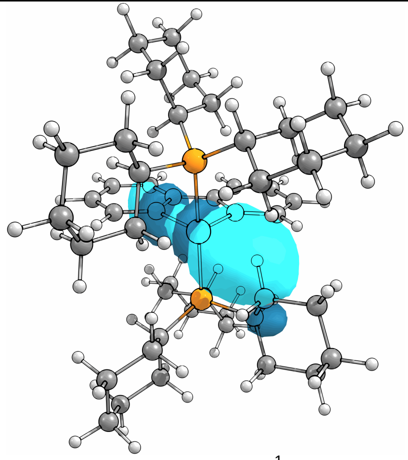 10.05 kcal·mol^{-1}	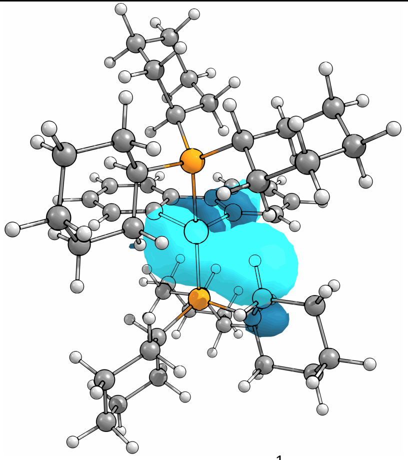 < 0.05 kcal·mol^{-1}	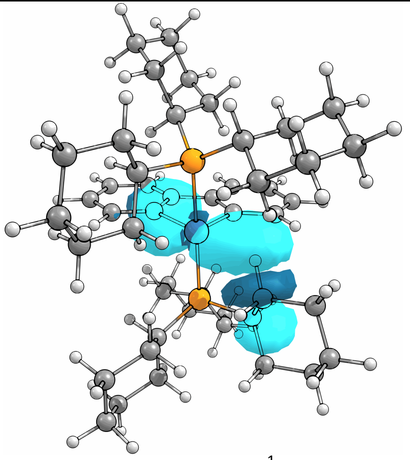 3.12 kcal·mol^{-1}	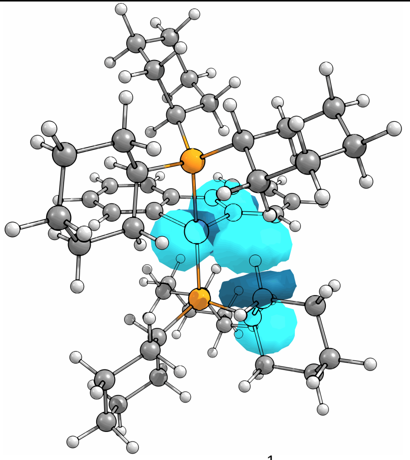 3.66 kcal·mol^{-1}

Table S8: Selected NBO orbital overlaps (isovalue 0.04) for **1c'**

**Agostic**	**$\sigma_{CH}\rightarrow\sigma^*_{MC}$**		**$\sigma_{MC}\rightarrow\sigma^*_{CH}$**	
	trans	*cis*	*trans*	*cis*
major	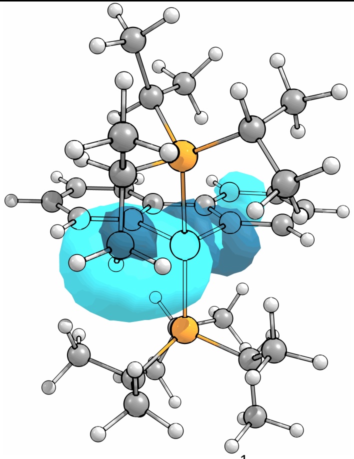<br>8.89 kcal·mol^{-1}	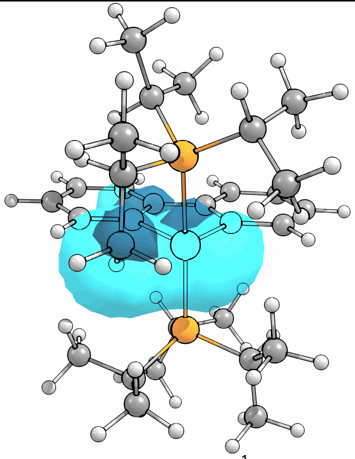<br>0.05 kcal·mol^{-1}	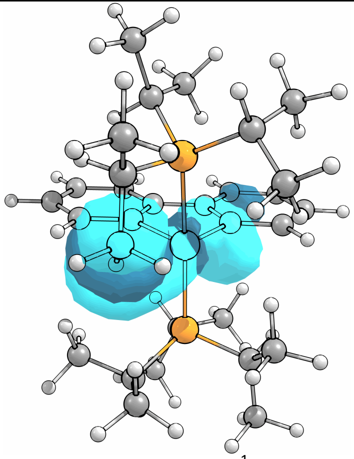<br>1.56 kcal·mol^{-1}	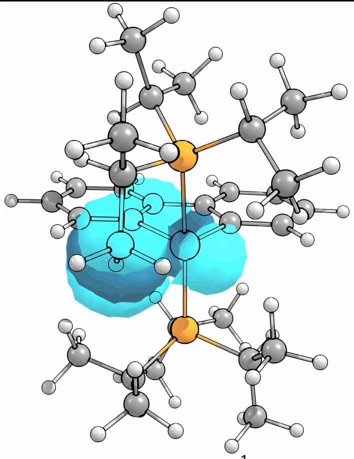<br>1.37 kcal·mol^{-1}
minor	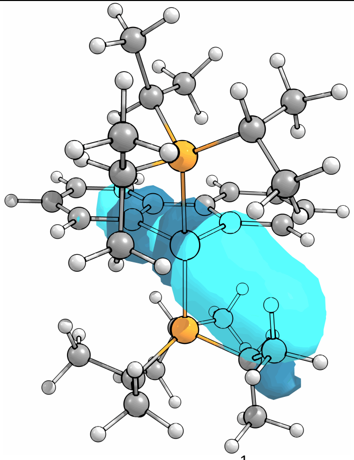<br>1.95 kcal·mol^{-1}	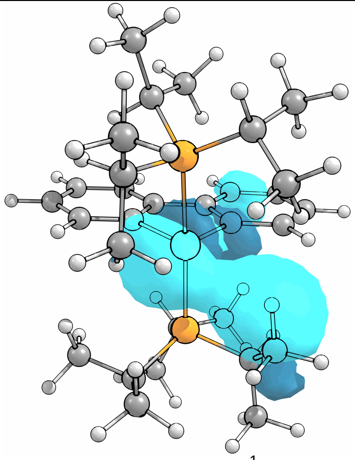<br>< 0.05 kcal·mol^{-1}	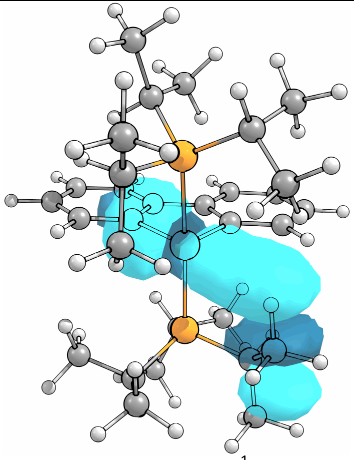<br>0.69 kcal·mol^{-1}	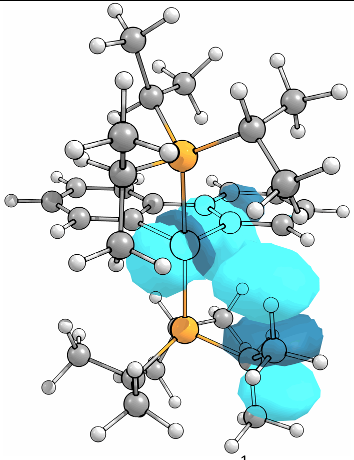<br>1.21 kcal·mol^{-1}

Table S9: Selected NBO orbital overlaps (isovalue 0.04) for **2c’**

**Agostic**	$\sigma_{CH}\rightarrow\sigma^*_{MC}$ *trans*	*cis*	$\sigma_{MC}\rightarrow\sigma^*_{CH}$ *trans*	*cis*
major	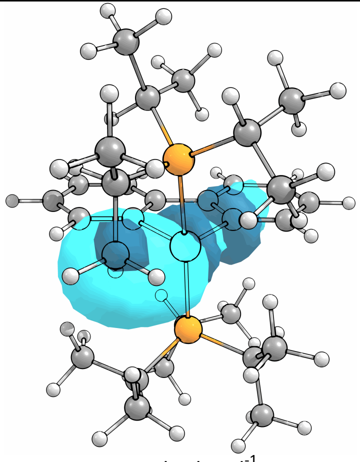 11.75 kcal·mol^{-1}	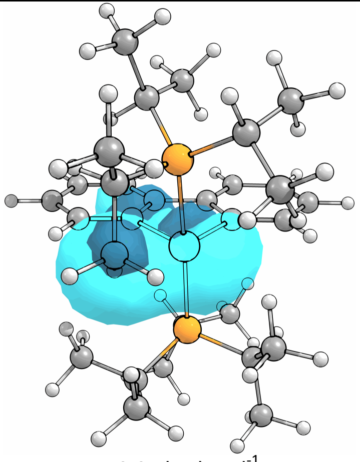 < 0.05 kcal·mol^{-1}	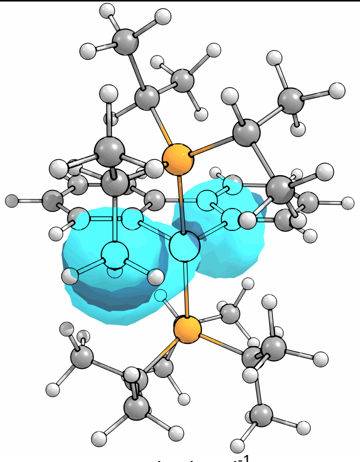 1.44 kcal·mol^{-1}	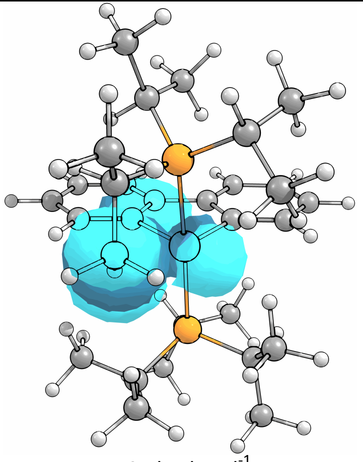 1.94 kcal·mol^{-1}
minor	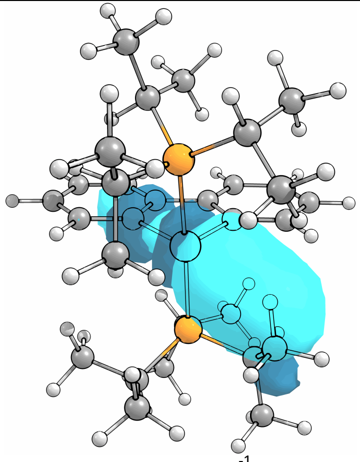 3.24 kcal·mol^{-1}	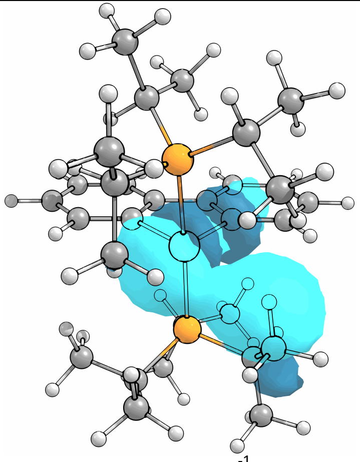 0.06 kcal·mol^{-1}	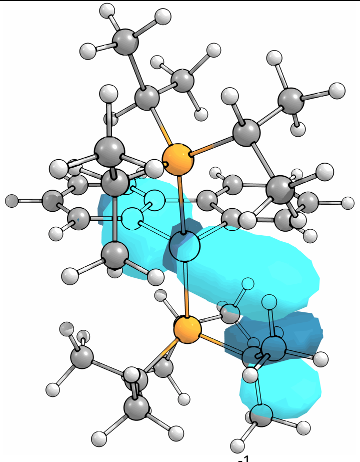 1.59 kcal·mol^{-1}	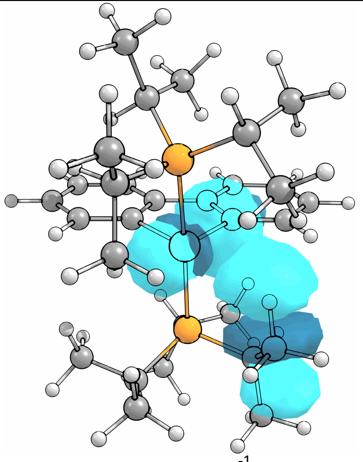 1.97 kcal·mol^{-1}

Table S10: Selected NBO orbital overlaps (isovalue 0.04) for **1d’**

**Agostic**	$\sigma_{CH} \rightarrow \sigma^*_{MC}$ *trans*	$\sigma_{CH} \rightarrow \sigma^*_{MC}$ *cis*	$\sigma_{MC} \rightarrow \sigma^*_{CH}$ *trans*	$\sigma_{MC} \rightarrow \sigma^*_{CH}$ *cis*
major	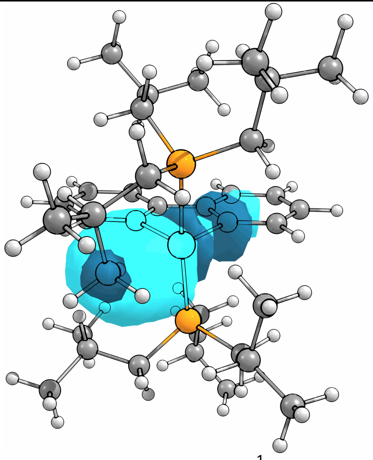 14.91 kcal·mol^{-1}	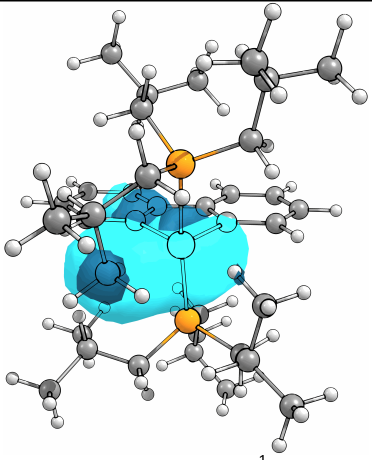 < 0.05 kcal·mol^{-1}	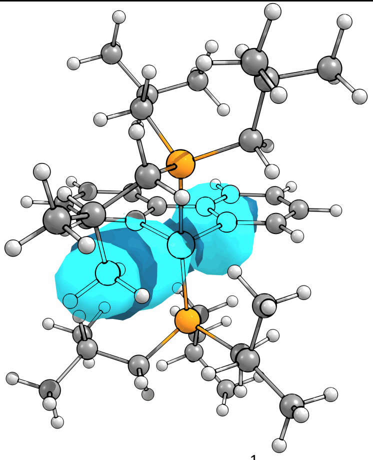 3.33 kcal·mol^{-1}	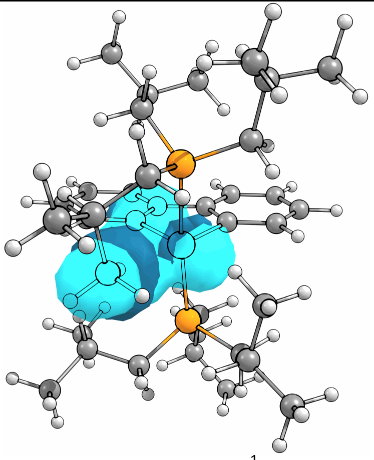 3.85 kcal·mol^{-1}
minor	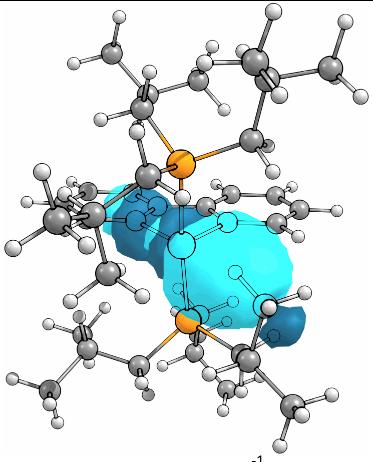 11.57 kcal·mol^{-1}	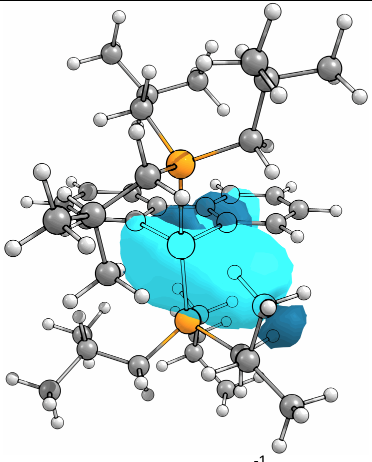 < 0.05 kcal·mol^{-1}	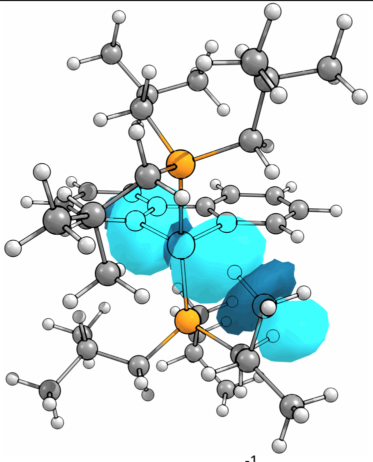 5.02 kcal·mol^{-1}	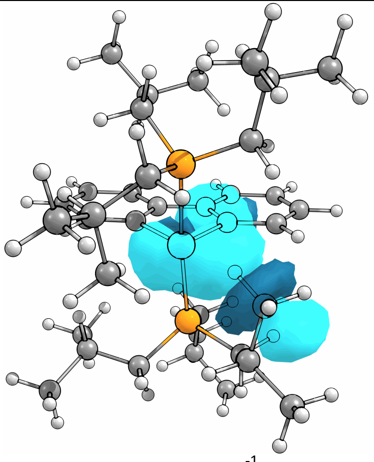 4.45 kcal·mol^{-1}

Table S11: Selected NBO orbital overlaps (isovalue 0.04) for **2d'**

**Agostic**	$\sigma_{CH}\rightarrow\sigma^*_{MC}$ *trans*	$\sigma_{CH}\rightarrow\sigma^*_{MC}$ *cis*	$\sigma_{MC}\rightarrow\sigma^*_{CH}$ *trans*	$\sigma_{MC}\rightarrow\sigma^*_{CH}$ *cis*
major	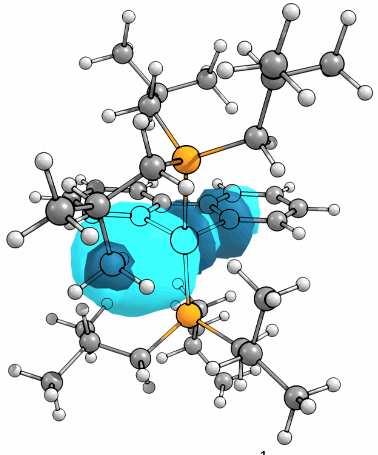 19.19 kcal·mol^{-1}	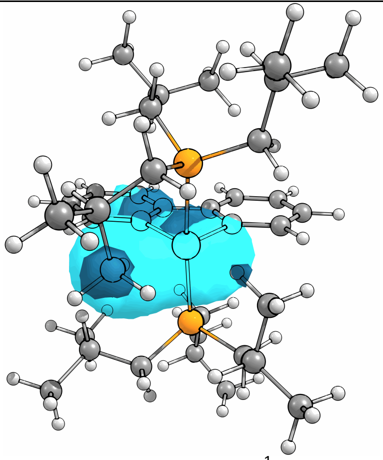 < 0.05 kcal·mol^{-1}	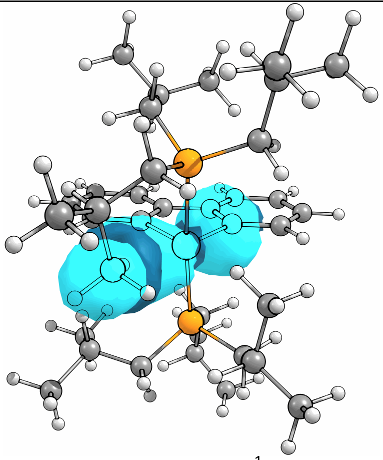 5.31 kcal·mol^{-1}	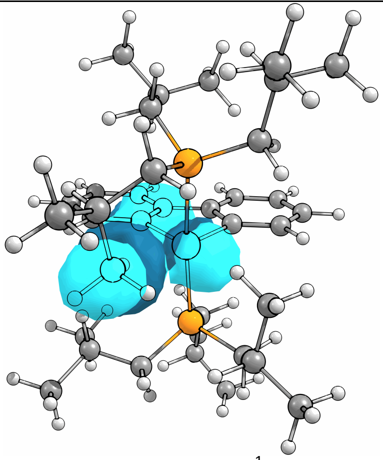 5.50 kcal·mol^{-1}
minor	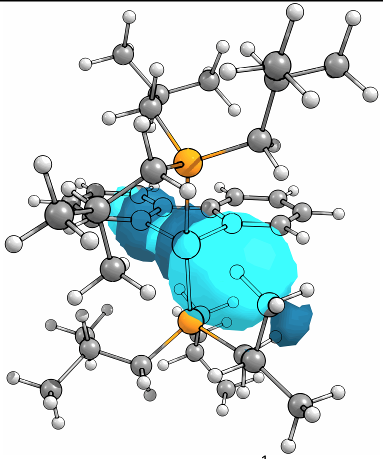 16.38 kcal·mol^{-1}	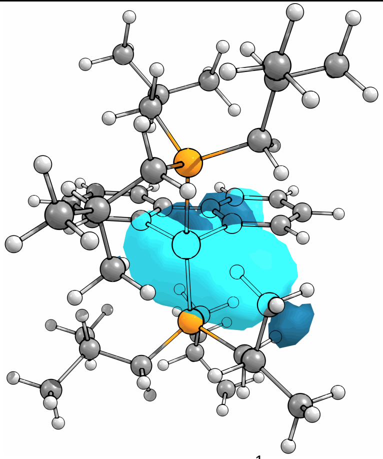 0.56 kcal·mol^{-1}	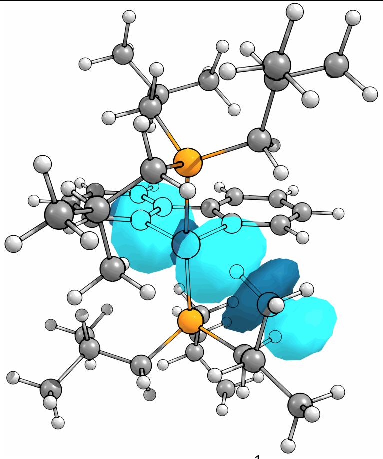 9.35 kcal·mol^{-1}	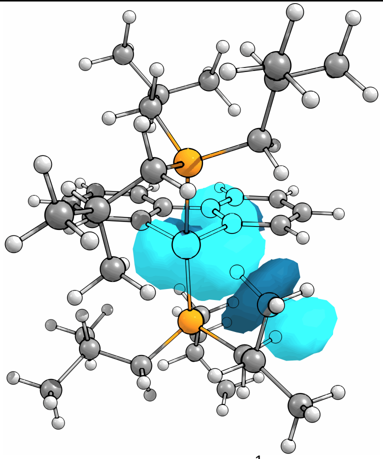 7.35 kcal·mol^{-1}

5.2. *QTAIM molecular graphs*

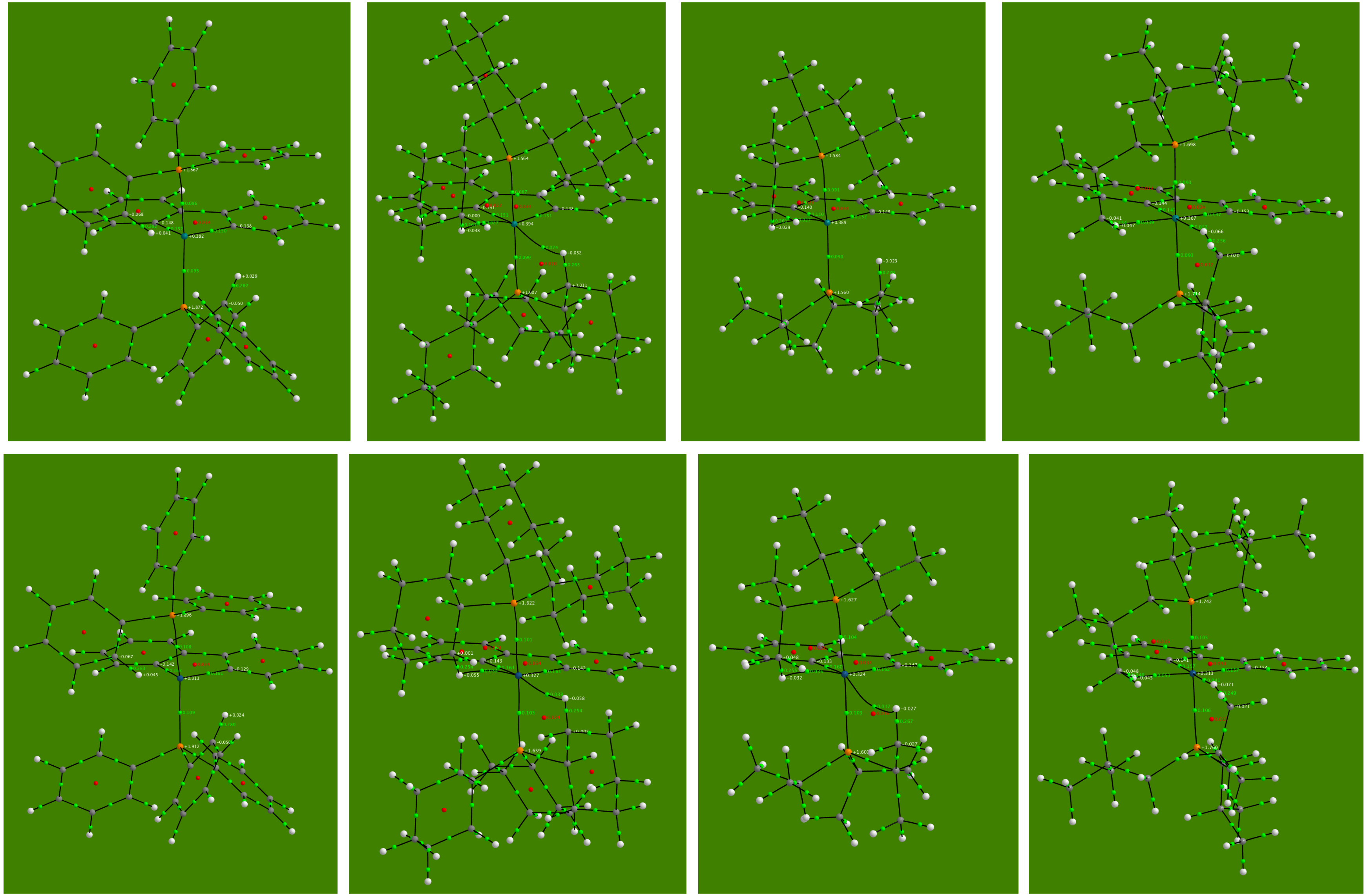

Figure S91: QTAIM molecular graphs of **1a–d'** (top, left to right) and **2a–d'** (bottom, left to right); bond critical points and selected electron densities in green, ring critical points and selected electron densities in red, selected atomic charges in white.
